# Extracellular Vesicle‐Packaged circTAX1BP1 from Cancer‐Associated Fibroblasts Regulates RNA m6A Modification through Lactylation of VIRMA in Colorectal Cancer Cells

**DOI:** 10.1002/advs.202514008

**Published:** 2025-09-29

**Authors:** Jia‐nan Tan, Jin‐hao Yu, Dong Hou, Ye‐quan Xie, Dong‐ming Lai, Fang Zheng, Bin Yang, Jin‐tao Zeng, Yang Chen, Shu‐hong Lu, Guang‐yu Zhong, Fang‐hai Han, Sheng‐ning Zhou

**Affiliations:** ^1^ Department of Gastrointestinal Surgery The Affiliated Guangdong Second Provincial General Hospital of Jinan University Guangzhou 510317 China; ^2^ Department of Gastrointestinal Surgery Sun Yat‐Sen Memorial Hospital Sun Yat‐Sen University Guangzhou 510120 China; ^3^ Laboratory for Precision Diagnosis and Treatment of Gastrointestinal Tumors The Affiliated Guangdong Second Provincial General Hospital of Jinan University Guangzhou 510317 China; ^4^ Medical Research Center Sun Yat‐Sen Memorial Hospital Sun Yat‐Sen University Guangzhou Guangzhou 510120 China

**Keywords:** cancer‐associated fibroblasts, circRNAs, colorectal cancer, extracellular vesicles, tumor microenvironment

## Abstract

The underlying molecular mechanism of patients with colorectal liver metastasis (CRLM) remains unclear. In this study, it is found that cancer‐associated fibroblasts (CAFs)‐derived extracellular vesicles (EVs) are significantly enriched in circTAX1BP1 in CRLM, which associate with poor prognosis. The disruption of EV‐packaged circTAX1BP1 significantly inhibits CRLM in vivo and in vitro. Mechanistically, CAF‐derived EV‐packaged circTAX1BP1 is delivered to colorectal cancer (CRC) cells, where it binds to VIRMA and promotes its lactylation at lysine residue 1713 by recruiting AARS2. Lactylated VIRMA enhances m^6^A‐based modification and stability of SP1 mRNA. SP1 mediates the transcription of TGF‐β, enhancing epithelial–mesenchymal transition and paracrine TGF‐β of CRC cells. Notably, this study identifies an important subgroup ITGA11^+^ myCAFs through single‐cell RNA sequencing data. Paracrine TGF‐β of CRC cells specifically targets ITGA11^+^ myCAFs, activating the TGF‐β signalling pathway, which contributes to extracellular matrix remodeling and increases delivery of EV‐packaged circTAX1BP1, forming a positive feedback loop to promote CRLM. Finally, the combined blockade of EV‐packaged circTAX1BP1 and TGF‐β can effectively disrupt this feedback loop and significantly inhibit tumor progression in a PDX model. Overall, this study provides an in‐depth understanding of tumor cell‐CAFs crosstalk and new insights into therapeutic targets for CRLM.

## Graphical abstract

This study reveals that CAFs‐derived EVs enriched with circTAX1BP1 drive CRLM by promoting VIRMA lactylation and SP1/TGF‐β signaling, creating a positive feedback loop via ITGA11^+^ myCAFs. Targeting EV‐circTAX1BP1 and TGF‐β disrupts this loop, offering a novel therapeutic strategy for CRLM.

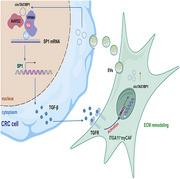

## 1 Introduction

Colorectal cancer (CRC) is the second leading cause of cancer‐related deaths worldwide, and metastasis is the main cause of CRC‐related death.^[^ ^1^ ^]^ Despite the considerable advances in surgical and drug treatments, liver metastasis still occurs in ≈50% of patients with CRC.^[^ ^2^ ^]^ Radical resection and chemotherapy remain the standard treatment options for patients with CRC liver metastasis (CRLM); however, the 5‐year survival rate is only 30%.^[^ ^3^ ^]^ Alarmingly, more than half of patients would be beyond the window for curative treatment at the time of diagnosis.^[^ ^4^ ^]^ Therefore, more studies are urgently needed to identify new biomarkers and targeted therapies to improve the survival of patients with CRLM.

As a crucial part of the tumor microenvironment (TME), cancer‐associated fibroblasts (CAFs) exert multiple functions,^[^ ^5^ ^]^ including extracellular matrix (ECM) deposition and remodeling,^[^ ^6^ ^]^ lymphangiogenesis induction,^[^ ^7^ ^]^ antigen presenting,^[^ ^8^ ^]^ and complex signalling interactions with tumors.^[^ ^9^, ^10^ ^]^ CAFs are potential combined targets for optimizing single anti‐tumor cell strategies. The mechanisms of CAFs vary in different TMEs. O¨hlund et al. used single‐cell RNA sequencing (scRNA‐seq) to identify myofibroblastic CAFs (myCAFs) and inflammatory CAFs (iCAFs) based on gene expression and functional classification,^[^ ^11^ ^]^ highlighting the potential of single‐cell technology in analyzing the TME. However, more studies are needed to characterize further precise subtypes of CAFs and elucidate the relationships between CAF subtypes and other components.

The exchange of molecules and transmission of signals between cells involves extracellular vesicles (EVs).^[^ ^12^ ^]^ Depending on the source cells, EVs contain various cellular components, including DNA, RNA, lipids, metabolites, and cytoplasmic or cell surface proteins. Accumulating evidence indicates that CAFs communicate with other cells through EVs in the TME.^[^ ^13^ ^]^ Notably, the non‐coding RNAs contained in EVs are characterized by targeted and functional regulation.^[^ ^14^ ^]^ For instance, Yao et al. found that EV‐packed lncRNA RP11‐161H23.5 derived from CAFs promotes immune escape in pancreatic ductal adenocarcinoma (PDAC) by downregulating the key antigen‐presenting molecule HLA‐A in pancreatic cancer cells.^[^ ^15^ ^]^ Kang et al. discovered that CAF‐derived EVs promote cisplatin resistance in oral squamous cell carcinoma via miR‐876‐3p.^[^ ^16^ ^]^ Zheng et al. found that CAF‐derived EVs deliver circBIRC6 and enhance the SUMOylation modification of XRCC4 at lysine 115 and promote its nuclear translocation, thereby inducing oxaliplatin resistance in PDAC.^[^ ^17^ ^]^ These findings indicate that CAFs play a key role in TME remodelling through EVs.

RNA chemical modification is a widespread biological process in eukaryotic cells, especially N6‐methyladenosine (m^6^A), the most common RNA modification. A large body of evidence indicates that the dysregulation of m^6^A modification and its target proteins promotes cancer development.^[^ ^18^ ^]^ Some recent studies have found that m^6^A methyltransferases can undergo common post‐translational modifications (PTMs), including acetylation, SUMOylation, and ubiquitination, which significantly affect m^6^A methyltransferase in RNA modification.^[^ ^19^, ^20^, ^21^ ^]^ The discovery of lactylation, a novel PTM, has provided new insights into the mechanisms promoting tumor initiation and progression.^[^ ^22^, ^23^, ^24^ ^]^ Lactylation was first discovered in histones, and it regulates gene transcription; however, subsequent studies have shown that it occurs on non‐histone proteins. Notably, limited research has demonstrated that m^6^A methyltransferases undergo lactylation. In CRC, lactylation modification of METTL3 in tumor‐infiltrating myeloid cells promotes m^6^A‐dependent JAK–STAT3 axis activation and inhibits tumor immunity.^[^ ^25^ ^]^ In gastric cancer, lactylation modification of METTL16 promotes FDX1 m^6^A‐dependent mRNA stability and promotes cuproptosis.^[^ ^26^ ^]^ VIRMA is a methyltransferase that acts as an m^6^A writer and promotes tumorigenesis in various cancers in an m^6^A‐dependent manner;^[^ ^27^ ^]^ however, its underlying mechanism in CRLM remains unclear.

In the present study, circTAX1BP1 was significantly enriched in CAF‐derived EVs and associated with patient prognosis in CRLM. Furthermore, CAF‐derived EV‐packaged circTAX1BP1 was transferred to CRC cells, contributing to CRC progression and activating ITGA11^+^ myCAFs through paracrine TGF‐β. Mechanistically, CAF‐derived EV‐packaged circTAX1BP1 binds to VIRMA and recruits the lactate transferase alanyl‐tRNA synthetase 2 (AARS2) to promote lactylation modification at lysine residue 1713 of VIRMA. Lactylated VIRMA enhances the stability of SP1 mRNA in an m^6^A pathway‐dependent manner, thereby promoting TGF‐β transcription and enhancing CRC cell proliferation and metastasis. Notably, paracrine TGF‐β from CRC cells specifically activated the TGF‐β signalling pathway of ITGA11^+^ myCAFs, enhancing the secretion of circTAX1BP1 and ECM remodelling. These results suggest that combined intervention with EV‐packaged circTAX1BP1 and TGF‐β can serve as a therapeutic target for CRLM.

## 2 Results

### 2.1 Elevated Expression of circTAX1BP1 in CAFs in CRC Stroma is Associated with Liver Metastasis

**Figure 1:**
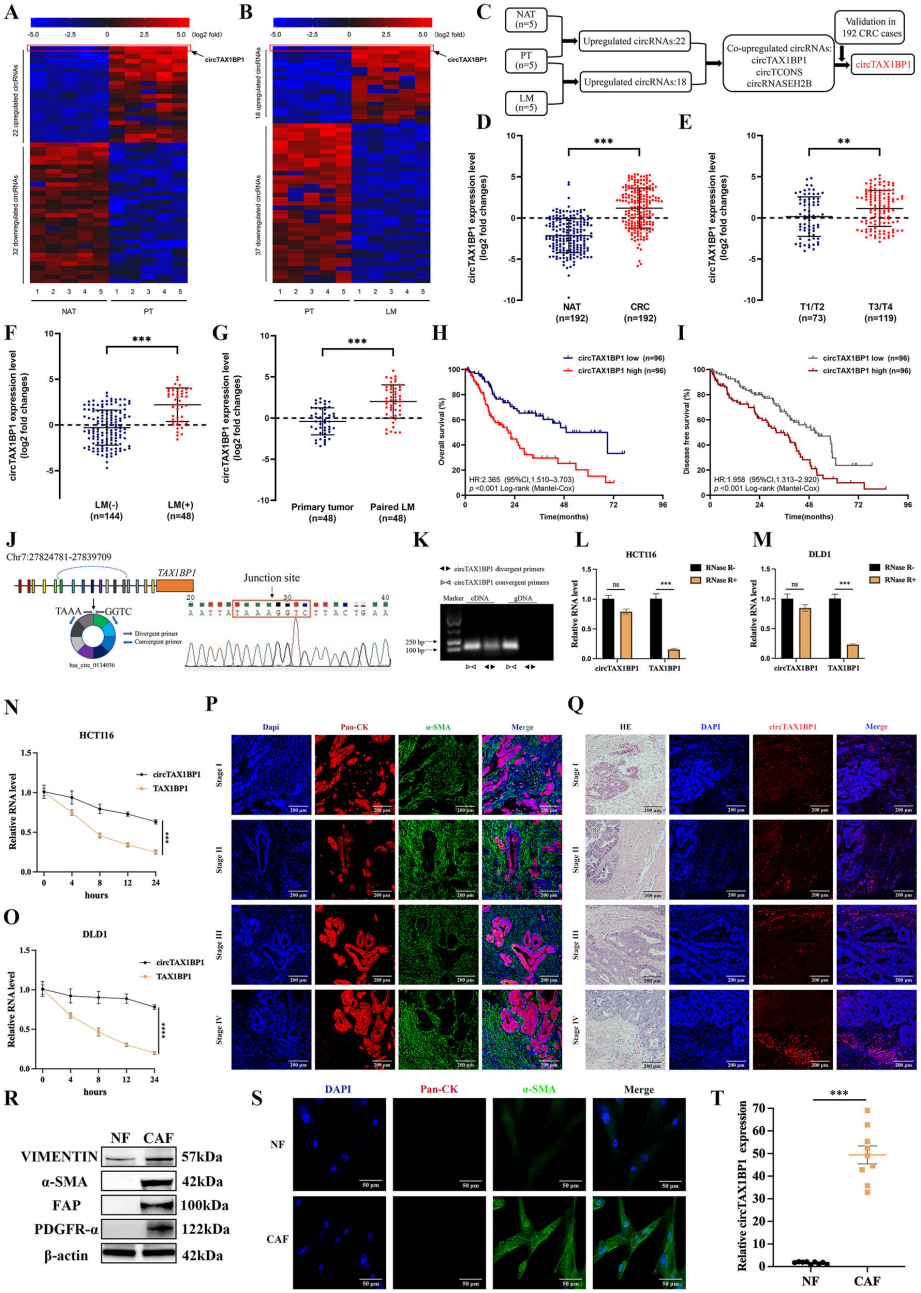
Elevated circTAX1BP1 expression in CAFs within the colorectal cancer stroma is associated with liver metastasis. A,B) Unsupervised hierarchical clustering of normalized counts from high‐throughput sequencing of normal adjacent tissues (NAT), primary tumors (PT), and liver metastasis (LM). The pseudocolor represents the intensity scale of PT versus NAT or the LM versus PT, generated by a log2 transformation. C) Schematic representation of circTAX1BP1 upregulation in colorectal cancer (CRC) tissues and LM‐positive tissues. D) qRT‐PCR analysis of circTAX1BP1 expression in a 192‐case cohort of freshly collected human CRC samples and NATs. E,F) Correlation of circTAX1BP1 expression in CRC tissues (*n* = 192) assessed using qRT‐PCR with pathological grade (E) and LM status (F). G) Comparison of circTAX1BP1 expression in primary human CRC samples and paired metastatic LMs. H,I) Kaplan–Meier curves for OS (H) and DFS (I) of patients with CRC with low vs. high expression of circTAX1BP1. The median expression level of circTAX1BP1 was taken as the cutoff value. *p*‐values were calculated by the log‐rank (Mantel–Cox) test. J) Schematic of circTAX1BP1 formation. The circTAX1BP1 back‐splicing junction was identified through Sanger sequencing. K) circTAX1BP1 was present in HCT116, as determined via qRT‐PCR using convergent and divergent primers. L,M) qRT‐PCR confirmation of circTAX1BP1 stability after RNase R treatment (*n* = 3). N,O) Following treatment with actinomycin D, the half‐lives of circTAX1BP1 and linear TAX1BP1 were measured (*n* = 3). P) CRC tissues were stained with pan‐cytokeratins (cancer nest) and α‐SMA (stroma) through immunofluorescence assay. Scale bar, 200 µm. Q) In situ analysis with Cy3‐labelled RNA using a circTAX1BP1 FISH probe in CRC tissues. Scale bar, 200 µm. R) Protein levels of vimentin, α‐SMA, FAP and PDGFR‐α were detected using western blotting in isolated CAFs and NFs. S) Immunofluorescence staining showed the subcellular location and the expression of pan‐cytokeratins and α‐SMA in isolated CAFs and NFs. Scale bar, 50 µm. T) qRT‐PCR was used to analyze circTAX1BP1 level in isolated CAFs and NFs (*n* = 9). The statistical difference was assessed through the nonparametric Mann–Whitney *U*‐test in (D–G); and 2‐tailed Student's *t*‐test in (L–O,T). All data are presented as mean ± SD of experimental triplicates. ns, *P* > 0.05; **, *P* < 0.01; ***, *P *< 0.001.

To identify critical circular RNAs (circRNAs) implicated in CRC progression, we performed transcriptome sequencing on five pairs of normal adjacent tissues (NAT), primary colorectal tumor (PT) tissues, and liver metastatic (LM) tissues. As illustrated in **Figure** **1**A,B, PT tissues exhibited 22 significantly upregulated and 32 downregulated circRNAs (>fivefold change) compared to NAT tissues. In contrast, 18 circRNAs were upregulated and 37 downregulated in LM tissues versus PT tissues (>fivefold change). Notably, three circRNAs—circTAX1BP1, circTCONS, and circRNASEH2B—demonstrated consistent upregulation in PT and LM tissues (Figure 1C). To further validate these findings, we analyzed 192 CRC tissues and their paired NATs using quantitative reverse transcription PCR (qRT‐PCR). Among the candidates, only circTAX1BP1 exhibited significant overexpression in CRC tissues compared to NATs (Figure 1D; Figure S1A,B, Supporting Information). Furthermore, higher circTAX1BP1 expression was observed in advanced local invasion stages (T3/T4) compared to early stages (T1/T2), suggesting its involvement in promoting tumor aggressiveness within the primary site (Figure 1E). Statistical analysis revealed a strong correlation between circTAX1BP1 expression and liver metastasis status (Figure 1F, Table S1, Supporting Information). qRT‐PCR analysis further confirmed that circTAX1BP1 was markedly overexpressed in LM tumor cells compared to paired primary tumors (Figure 1G), suggesting its potential role as a key component of metastatic cells. Notably, high circTAX1BP1 expression was associated with poorer overall survival (OS) and disease‐free survival (DFS) in patients with CRC (Figure 1H,I). Univariate and multivariate Cox proportional hazards analyses demonstrated that circTAX1BP1 expression was an independent prognostic factor for OS (Table S2, Supporting Information) and DFS (Table S3, Supporting Information) in patients with CRC. In addition, circTAX1BP1 was significantly overexpressed in various human cancers, including bladder, renal, breast, gastric, and liver cancers (Figure S1C–G, Supporting Information). Meanwhile, analysis of plasma exosomal sequencing data revealed that circTAX1BP1 is highly expressed in colorectal cancer (Figure S1H,I, Supporting Information). This finding further suggests that circTAX1BP1 may play an oncogenic role in the progression and development of several human cancer types.

circTAX1BP1, annotated as hsa_circ_0134036 (https://circinteractome.nia.nih.gov/), is derived from the TAX1BP1 gene (Gene ID: 8887; genomic locus: chr7:27,778,992–27,869,386; https://www.ncbi.nlm.nih.gov/gene/8887). The linear TAX1BP1 transcript spans 14 928 bp (chr7:27,824,781–27,839,709). Furthermore, circTAX1BP1 is generated via head‐to‐tail splicing of exons 6–13, resulting in a circular transcript of 1152 bp. Sanger sequencing confirmed the presence of the back‐splice junction of circTAX1BP1 (Figure 1J). To distinguish circTAX1BP1 from its linear counterpart, we designed convergent and divergent primers to amplify TAX1BP1 mRNA and circTAX1BP1 from cDNA and genomic DNA (gDNA) of HCT116 cells (Figure 1K). The results revealed that circTAX1BP1 was detectable in cDNA but absent in gDNA, confirming its circular nature. Furthermore, circTAX1BP1 exhibited resistance to RNase R treatment, whereas linear TAX1BP1 mRNA levels were significantly reduced (Figure 1L,M). Following actinomycin D treatment, circTAX1BP1 had a longer half‐life compared to linear TAX1BP1 mRNA (Figure 1N,O). These findings collectively indicate that circTAX1BP1 is a stable circRNA resistant to RNAse degradation.

To investigate the origin and role of circTAX1BP1 in CRC tissues, we utilized immunofluorescence (IF) staining and confirmed the presence of abundant stromal components in tissues from patients with CRC at various stages (Figure 1P). Notably, circTAX1BP1 exhibited significantly higher expression in the tumor stroma compared to the cancer nests (Figure 1Q). Moreover, as the disease stage advanced, the expression level of circTAX1BP1 in the stroma increased accordingly (Figure S1J, Supporting Information). To further confirm circTAX1BP1 expression in the tumor stroma, we isolated CAFs and NFs from CRC tissues and NATs, respectively. Fibroblast markers were assessed to validate the purity and phenotype of the isolated NFs and CAFs. In CAFs, the expressions of genes characteristic of fibroblast activation, including α‐smooth muscle actin (ACTA2), fibroblast activation protein (FAP), fibroblast‐specific protein 1 (FSP1), and CD90, were significantly elevated compared to their NF counterparts (Figure S1K, Supporting Information). Both NFs and CAFs expressed vimentin, a mesenchymal marker, whereas CAFs markers—α‐SMA, FAP, and platelet‐derived growth factor receptor α (PDGFR‐α)—were specifically expressed in CAFs (Figure 1R). IF staining confirmed the cellular morphology and marker expression in the isolated fibroblast populations (NFs and CAFs) (Figure 1S). Notably, we observed significantly higher expression of circTAX1BP1 in CAFs than in NFs (Figure 1T). These findings indicate that circTAX1BP1 is highly expressed in CAFs, a key component of the tumor stroma in CRC.

### 2.2 Exosomal circTAX1BP1 Derived from CAFs Enhances Proliferation, Migration, and Invasion of CRC Cells In Vitro and In Vivo

**Figure 2:**
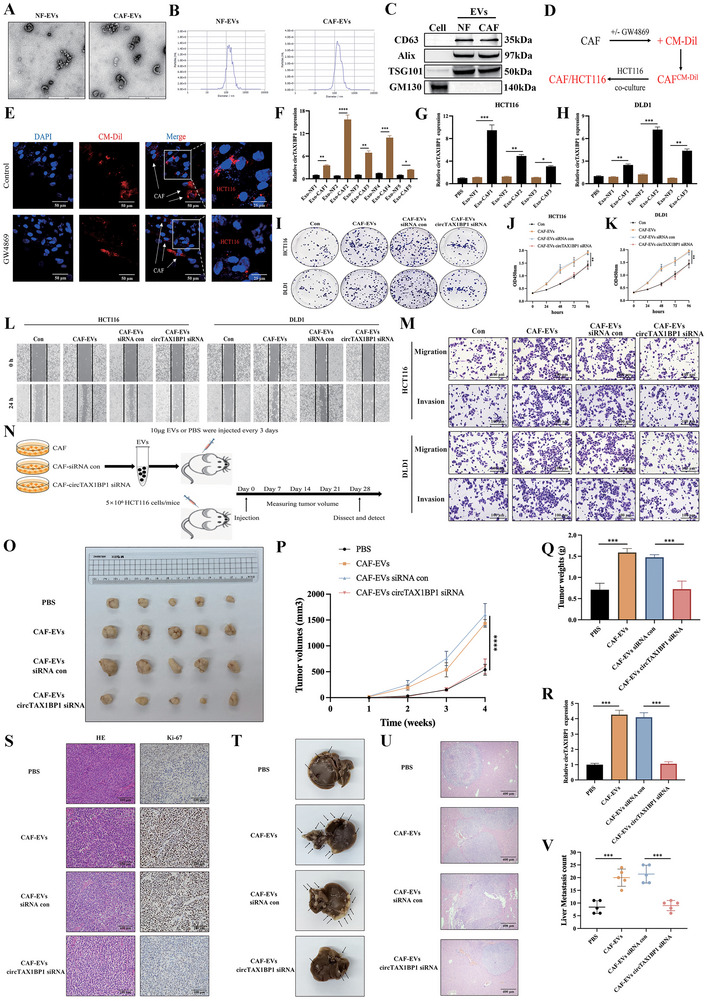
CAF‐derived exosomal circTAX1BP1 enhances the proliferation, migration, and invasion of colorectal cancer (CRC) cells. A) Representative images of CAF‐ and NF‐derived exosomes analyzed via transmission electron microscopy. Scale bar, 600 nm. B) Size distribution of the isolated exosomes analyzed via nanoparticle tracking. C) Western blotting analysis for exosomal markers CD63, ALIX, TSG101, and GM130 of HCT116 cells and CAF‐ or NF‐derived exosomes. D,E) CAFs were pre‐treated with or without GW4869. CAFs were labelled with CM‐Dil (red) and co‐cultured with HCT116 for 18 h. Immunofluorescence analysis of CM‐Dil distribution in cells. Scale bar, 50 µm. Scale bars insets, 25 µm. F) qRT‐PCR was used to analyze circTAX1BP1 level in the isolated CAF‐ and NF‐derived exosomes of five patients (*n* = 3). G,H) HCT116 and DLD1 cells were incubated with indicated exosomes or PBS for 48 h, and circTAX1BP1 level was analyzed using qRT‐PCR (*n* = 3). I) Colony formation assays were performed for HCT116 and DLD1 cells. J,K) Proliferation of HCT116 and DLD1 cells was measured using CCK‐8 assays (*n* = 3). L) Representative images of wound healing assay using HCT116 and DLD1 cells showing cell motility. M) Representative images of Transwell assays, and migration and invasion were determined for HCT116 and DLD1 cells. Scale bar, 100 µm. N) Schematic of the xenograft nude mice model. O) Images of xenograft tumors harvested from nude mice. P) Tumor volume summary for mice, measured every 7 days (*n* = 5). Q) Nude mice were examined for tumor weights (*n* = 5). R) circTAX1BP1 levels were assessed using qRT‐PCR in xenograft tumors harvested from nude mice (*n* = 5). S) IHC staining for Ki67 was measured (*n* = 5) in xenograft tumors harvested from nude mice. Scale bar, 100 µm. T) Representative livers were acquired from the nude mice (*n* = 5). U,V) Representative images of metastatic nodules, and measurement of metastatic nodules of mouse livers were examined using haematoxylin–eosin staining (*n* = 5). Scale bar, 400 µm. The statistical difference was assessed by a 2‐tailed Student's *t*‐test in (F–H,Q,R,V); and one‐way ANOVA followed by Dunnett tests in (J,K,P). All data are presented as mean ± SD of experimental triplicates. *, *P* < 0.05; **, *P* < 0.01; ***, *P* < 0.001; ****, *P *< 0.0001.

To further elucidate the role of CAF‐derived exosomes, we purified exosomes from conditioned media (CM) of CAFs and NFs. Both isolated exosome populations exhibited typical exosomal characteristics, including a lipid bilayer membrane, cup‐shaped morphology, and consistent size and quantity (**Figure** **2**A,B). Immunoblotting confirmed the presence of exosomal marker proteins (CD63, TSG101, and ALIX) in the isolated exosomes (Figure 2C). To assess exosome uptake, we pre‐treated CAFs with GW4869 or its solvent control, labelled the exosomes with CM‐Dil, and co‐cultured them with HCT116 cells for 18 h. IF analysis revealed significantly higher CM‐Dil‐positive HCT116 cells when co‐cultured with solvent‐treated CAF‐derived exosomes compared to GW4869‐treated CAFs, which secreted fewer exosomes (Figure 2D,E). These findings indicate that HCT116 cells efficiently internalize CAF‐derived exosomes. Afterward, we quantified the circTAX1BP1 levels in the isolated exosomes. Notably, exosomes derived from CAFs of patients with CRC exhibited significantly higher circTAX1BP1 levels compared to those from matched NFs (Figure 2F). Co‐incubation of HCT116 and DLD1 cells with CAF‐derived exosomes resulted in a marked increase in intracellular circTAX1BP1 levels. In contrast, exosomes from NFs did not elevate circTAX1BP1 levels in these CRC cell lines (Figure 2G,H). Collectively, these results demonstrate that circTAX1BP1 is enriched in CAF‐derived exosomes and can be transferred to CRC cells.

To evaluate the effects of exosomes on CRC cell proliferation, migration, and invasion, we co‐incubated CRC cells with exosomes derived from CAFs or NFs. Compared with NF‐derived exosomes, CAF‐derived exosomes significantly enhanced the proliferative capacity of CRC cells (Figure S2A–D, Supporting Information). Furthermore, CAF‐derived exosomes promoted the migration and invasion abilities of HCT116 and DLD1 cells (Figure S2E–I, Supporting Information). To investigate the role of circTAX1BP1 in CAF‐derived exosomes, we transfected CAFs with circTAX1BP1‐specific small interfering RNAs (siRNAs) to deplete circTAX1BP1 in EVs. The siRNA with the highest knockdown efficiency was selected for subsequent experiments (Figure S2J, Supporting Information). Notably, EVs from circTAX1BP1‐silenced CAFs reduced the colony‐forming ability of HCT116 and DLD1 cells compared to EVs from control CAFs (Figure 2I; Figure S3A, Supporting Information). CCK‐8 assays revealed that downregulation of circTAX1BP1 in CAF‐derived EVs reversed their proliferative enhancement in HCT116 and DLD1 cells (Figure 2J,K). Moreover, treatment of HCT116 and DLD1 cells with EVs from circTAX1BP1‐silenced CAFs significantly impaired their migration and invasion capabilities (Figure 2L,M; Figure S3B–D, Supporting Information). Collectively, these findings demonstrate that CAFs secrete EVs enriched with circTAX1BP1, which facilitate the malignant progression of CRC cells by enhancing proliferation, migration, and invasion.

To investigate the in vivo role of circTAX1BP1 in CAF‐derived EVs on CRC progression, we established subcutaneous tumor xenografts in Balb/c nude mice using CRC cells (5 × 10^6^) combined with EVs derived from untreated, control siRNA‐transfected, or circTAX1BP1 siRNA‐transfected CAFs. Phosphate‐buffered saline (PBS) was used as a vehicle control. Mice were injected subcutaneously with EVs (10 µg) or PBS every 3 days to assess the effects of circTAX1BP1 on EV‐mediated tumor growth (Figure 2N). Compared to the PBS group, tumors in mice receiving EVs from untreated or control siRNA‐transfected CAFs exhibited significantly increased tumor volume and weight. In contrast, EVs from circTAX1BP1 siRNA‐transfected CAFs reversed this effect, resulting in smaller tumors (Figure 2O–Q). Consistently, qRT‐PCR analysis revealed lower circTAX1BP1 expression in tumors from the CAFs‐circTAX1BP1 siRNA group than from the CAF‐EVs group (Figure 2R). In addition, Ki67 staining was elevated in tumors from the CAF‐EVs groups but restored to basal levels in the CAFs‐circTAX1BP1 siRNA group, indicating reduced proliferation (Figure 2S; Figure S3E, Supporting Information). To further evaluate the metastatic potential of CAF‐derived EVs, we administered EVs via tail vein injection. As shown in Figure S3F (Supporting Information), CAF‐derived EVs significantly promoted liver metastasis of CRC cells, whereas EVs from circTAX1BP1 siRNA‐transfected CAFs reduced metastatic burden. Notably, the CAFs‐circTAX1BP1 siRNA group effectively suppressed the development of EV‐induced liver metastasis compared to the CAF‐EVs groups (Figure 2T–V). Collectively, these findings demonstrate that circTAX1BP1 enriched in CAF‐derived EVs promotes CRC tumor growth and liver metastasis in vivo, highlighting its critical role in tumor progression.

### 2.3 circTAX1BP1 in CAF‐Derived EVs Binds VIRMA and Promotes VIRMA Lactylation, Elevating *m* ^6^ *A* Levels in CRC Cells

**Figure 3:**
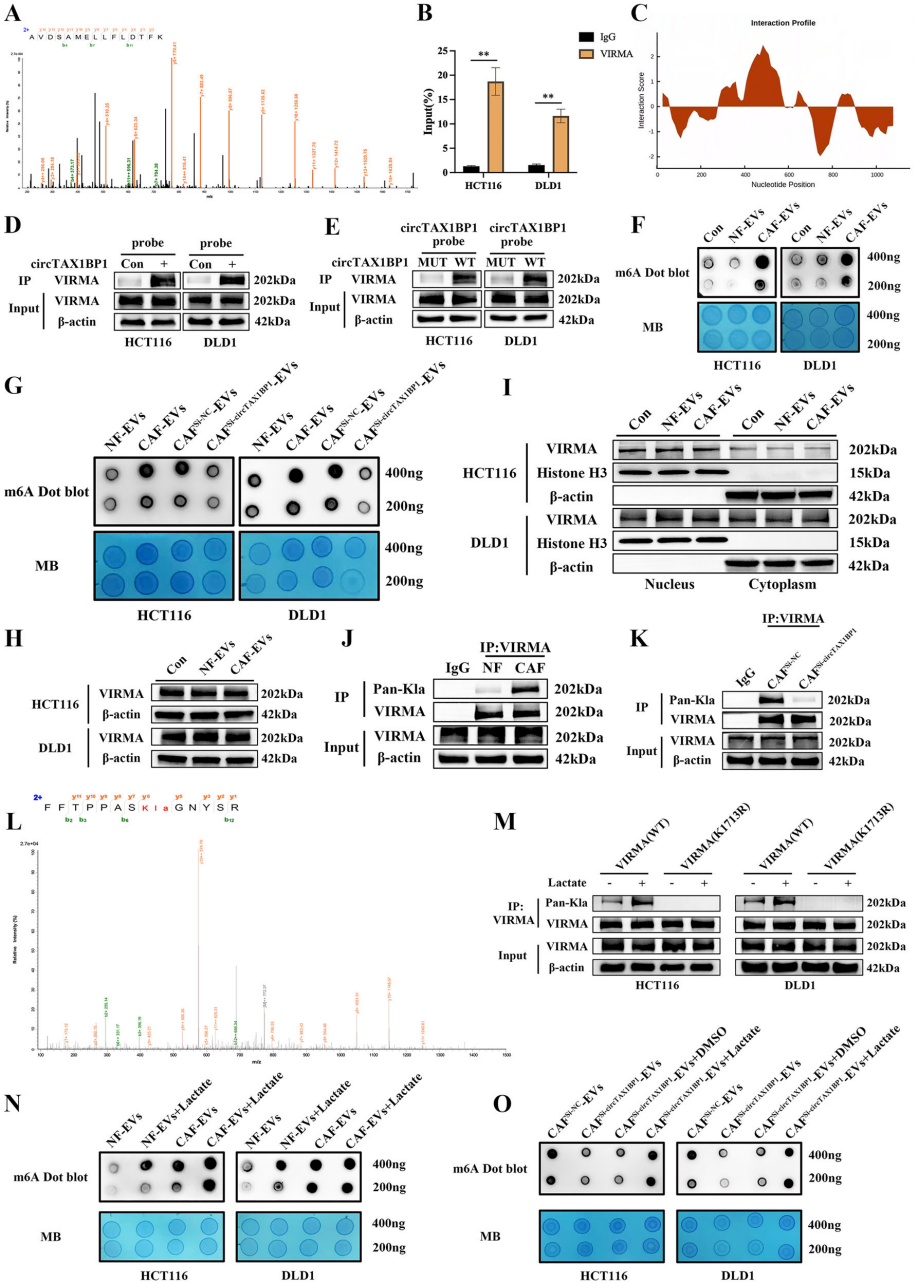
circTAX1BP1 in CAF‐derived EVs binds VIRMA and promotes VIRMA lactylation, elevating m^6^A levels in colorectal cancer (CRC). A) LC‐MS/MS analysis of circTAX1BP1 pulldown. B) Interaction of circTAX1BP1 with the m^6^A writer protein VIRMA was determined using RIP assays of HCT116 and DLD1 cells (*n* = 3). C) Interaction propensity prediction between circTAX1BP1 and VIRMA by CatRAPID. D) circTAX1BP1 and VIRMA interaction was verified using RNA pull‐down assays with HCT116 and DLD1 cells. circTAX1BP1 plasmid (5 µg) was transfected into 5 × 10^5^ cells using Lipofectamine 3000. E) A circTAX1BP1 Mut plasmid or a WT plasmid was transfected into HCT116 and DLD1 cells; subsequently, a circTAX1BP1 probe was used for RNA pull‐down assays. F,G) RNA m^6^A dot blot assays were used to assess the m^6^A levels of total mRNA. Methylene blue staining was used to determine the loading control. H) Relative VIRMA protein level was detected in HCT116 and DLD1 cells using western blotting. I) Western blot analyses were performed, and nuclear and cytoplasmic localizations of VIRMA were quantified for HCT116 and DLD1 cells. J,K) Cell lysates of HCT116 were immunoprecipitated with anti‐VIRMA or control IgG, followed by immunoblotting. L) Identification and quantification of VIRMA K1713 lactylation. LC–MS/MS analysis of modified FFTPPAS (Kla) GNYSR is shown. M) Flag‐VIRMA (WT) and Flag‐VIRMA (K1713R) cells were treated with lactate (25 mm) for 24 h, and whole‐cell extracts were collected for immunoprecipitation with anti‐VIRMA antibody, followed by immunoblotting. N,O) RNA m^6^A dot blot assays were used to assess the m^6^A levels of total mRNA. Methylene blue staining was used to determine the loading control. The statistical difference was assessed by a 2‐tailed Student's *t*‐test in (B). All data are presented as mean ± SD of experimental triplicates. **, *P* < 0.01.

To further explore the potential mechanism of circTAX1BP1 in CRC cells, RNA immunoprecipitation (RIP) analysis using an AGO2 antibody revealed no significant difference in enrichment between the AGO2 and IgG groups, suggesting that circTAX1BP1 does not function as a miRNA sponge (Figure S4A, Supporting Information). To identify proteins interacting with circTAX1BP1, we performed RNA pull‐down assays followed by mass spectrometry. We found that circTAX1BP1 specifically interacts with VIRMA (**Figure** **3**A; Figure S4B, Supporting Information). This interaction was further validated using RIP assays, confirming the binding of circTAX1BP1 to VIRMA in CRC cells (Figure 3B). Using the CatRAPID algorithm, we predicted the binding sites between circTAX1BP1 and VIRMA (Figure 3C), with detailed site information shown in Figure S4C–E (Supporting Information). RNA pull‐down assays demonstrated that the circTAX1BP1 probe pulled down significantly more VIRMA compared to the control probe (Figure 3D; Figure S4F, Supporting Information). Moreover, the binding affinity was enhanced in the wild‐type circTAX1BP1 group compared with the mutant group in CRC cells (Figure 3E; Figure S4G, Supporting Information). m^6^A dot blot assays revealed that CM from CAFs elevated m^6^A levels in CRC cells (Figure S5A, Supporting Information). Similar results were observed in CRC cells treated with CAF‐derived EVs (Figure 3F). Furthermore, the inhibition of circTAX1BP1 expression or EV secretion in CAFs reversed the EV‐ or CM‐induced increase in m^6^A levels in CRC cells (Figure 3G; Figure S5B, Supporting Information). Collectively, these findings demonstrate that CAFs secrete circTAX1BP1, which binds to VIRMA in CRC cells, thereby elevating m^6^A levels and promoting tumor progression.

Neither NF‐derived nor CAF‐derived EVs significantly affected VIRMA protein expression levels in CRC cells (Figure 3H; Figure S5C, Supporting Information). Similarly, no notable changes were observed in the nucleocytoplasmic distribution of VIRMA protein following treatment with NF‐ or CAF‐derived EVs (Figure 3I; Figure S5D,E, Supporting Information). Methyltransferase undergoes critical PTMs, such as lactylation and SUMOylation, which modulate its activity.^[^ ^20^, ^25^ ^]^ Given that metabolites can induce specific PTMs on proteins,^[^ ^28^ ^]^ we investigated whether circTAX1BP1 affects VIRMA PTMs in CRC cells. First, we immunoprecipitated total cell extracts from CRC cells using an anti‐VIRMA antibody, followed by immunoblotting with a pan‐Kla antibody to detect lactylation. VIRMA was lactylated in CRC cells, and its lactylation levels were significantly elevated in cancer cells incubated with CAF‐derived exosomes (Figure 3J; Figure S5F, Supporting Information). Notably, knockdown of circTAX1BP1 in CAFs attenuated VIRMA lactylation in CRC cells (Figure 3K; Figure S5G, Supporting Information). Liquid chromatography‐tandem mass spectrometry (LC‐MS/MS) analysis revealed the lactylation of lysine 1713 (K1713) on VIRMA (Figure 3L). To confirm whether VIRMA can be lactylated at K1713 in cells, we transiently transfected FLAG‐tagged wild‐type VIRMA (FLAG‐VIRMA) or a lactylation‐deficient mutant (FLAG‐VIRMA(K1713R)) into CRC cells and treated them with 25 mm lactate. Following CAF‐derived exosome treatment, we performed anti‐FLAG immunoprecipitation (IP). Western blotting demonstrated that the VIRMA(K1713R) mutant failed to undergo lactylation, even under lactate stimulation (Figure 3M). Collectively, these findings indicate that CAF‐secreted circTAX1BP1 directly interacts with VIRMA to elevate its lactylation at K1713, thereby modulating the methyltransferase activity of VIRMA in CRC cells.

Given that CAF‐secreted circTAX1BP1 regulates VIRMA lactylation, we investigated whether this modulation affects m^6^A levels in CRC cells. CRC cells were co‐incubated with exosomes derived from either NFs or CAFs, followed by lactate treatment. Notably, lactate significantly elevated intracellular m^6^A levels in CRC cells exposed to CAF‐derived exosomes (Figure 3N). Notably, lactate supplementation reversed the reduced m^6^A levels observed in CRC cells following knockdown of circTAX1BP1 in CAFs (Figure 3O). Collectively, these findings demonstrate that CAF‐derived circTAX1BP1 binds to VIRMA and promotes its lactylation at K1713, thereby increasing m^6^A levels in CRC cells.

### 2.4 CAF‐Derived EVs Deliver circTAX1BP1 to Recruit Lactyltransferase AARS2, Enhancing VIRMA Lactylation and Elevating m6A Levels in CRC Cells

**Figure 4:**
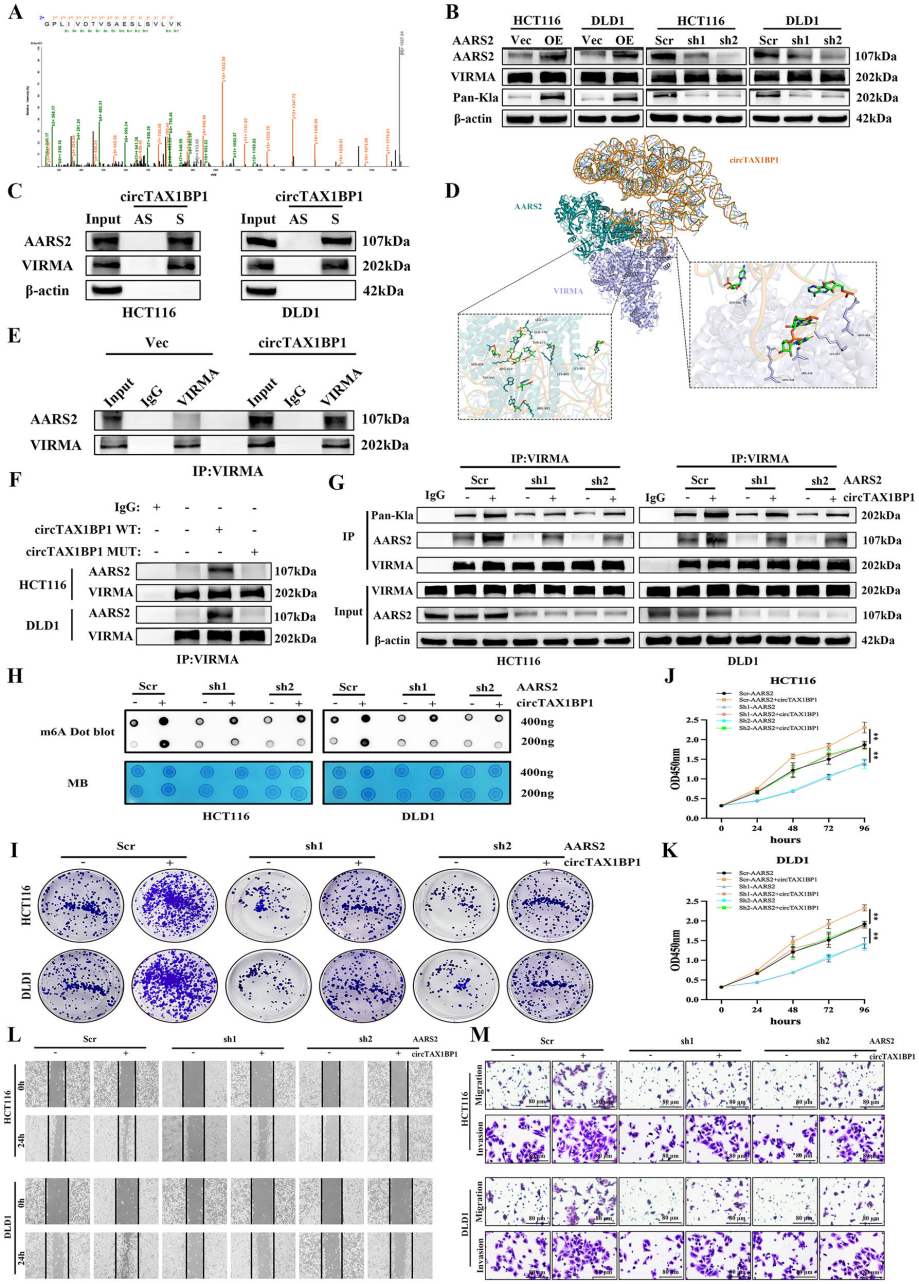
CAF‐derived EVs deliver circTAX1BP1 to recruit lactyltransferase AARS2, enhancing VIRMA lactylation and elevating m^6^A levels. A) LC‐MS/MS analysis of anti‐VIRMA IP. B) Effects of AARS2 overexpression or knockdown by two independent shRNAs on VIRMA lactylation expression. C) RNA pull‐down assays showing the association of the in vitro circularized circTAX1BP1 with VIRMA and AARS2. D) Structural model prediction of the circTAX1BP1‐VIRMA‐AARS2 ternary complex by AlphaFold3. E) circTAX1BP1 overexpression in CRC cells increased the association of AARS2 with VIRMA. F) The increased AARS2‐VIRMA association by circTAX1BP1 depends on the direct interaction between VIRMA and circTAX1BP1. G) Immunoblot assays to measure the expression of VIRMA lactylation and AARS2 proteins in HCT116 and DLD1 stable cell lines expressing circTAX1BP1 or AARS2 shRNA. H) RNA m^6^A dot blot assays were used to assess the m^6^A levels of total mRNA of different stable cells expressing circTAX1BP1 or AARS2 shRNA. I) Colony formation assays of different stable cells expressing circTAX1BP1 or AARS2 shRNA. J,K) CCK‐8 assays of different stable cells expressing circTAX1BP1 or AARS2 shRNA (*n* = 3). L) Wound healing assays of different stable cells expressing circTAX1BP1 or AARS2 shRNA. M) Cell migration and invasion assays of different stable cells expressing circTAX1BP1 or AARS2 shRNA. Scale bar, 80 µm. The statistical difference was assessed by one‐way ANOVA followed by Dunnett tests in (J,K). All data are presented as mean ± SD of experimental triplicates. **, *P* < 0.01.

To identify the enzyme responsible for VIRMA lactylation, we performed IP using an anti‐VIRMA antibody to capture proteins interacting with VIRMA from lysates of CRC cells co‐incubated with CAF‐derived exosomes. The eluted samples were analyzed using LC‐MS/MS (**Figure** **4**A). We identified AARS2, a mitochondrial enzyme encoded by the nuclear genome and primarily functioning in the mitochondria,^[^ ^29^ ^]^ as a potential lactyltransferase for VIRMA. Previous studies have reported AARS2 as a key lactyltransferase,^[^ ^30^ ^]^ which we experimentally validated in CRC cells by overexpressing or knocking down AARS2 (Figure 4B; Figure S6A–D, Supporting Information). We hypothesized that circTAX1BP1 recruits AARS2 and VIRMA to facilitate VIRMA lactylation, thereby increasing m^6^A levels. To test this hypothesis, we incubated biotinylated circTAX1BP1 probes with cell extracts. Both VIRMA and AARS2 were captured by the circTAX1BP1 probe (Figure 4C), indicating the formation of a circTAX1BP1–VIRMA–AARS2 complex. This finding aligns with our prior proteomic analysis identifying AARS2 as a potential interacting partner of circTAX1BP1 (Figure S4B, Supporting Information). Computational modelling using AlphaFold3 predicted a stable interaction among circTAX1BP1, VIRMA, and AARS2, supporting the formation of a ternary complex (Figure 4D). Furthermore, circTAX1BP1 overexpression significantly increased the interaction between AARS2 and VIRMA (Figure 4E; Figure S6E, Supporting Information). This enhancement required direct binding of VIRMA and circTAX1BP1, as forced expression of a VIRMA‐binding‐deficient circTAX1BP1 mutant failed to strengthen AARS2–VIRMA interaction (Figure 4F; Figure S6F,G, Supporting Information). Notably, circTAX1BP1‐induced VIRMA lactylation (Figure 4G; Figure S6H,I, Supporting Information) and m^6^A modification (Figure 4H) were attenuated following AARS2 knockdown using two independent shRNAs, confirming that AARS2 is necessary for VIRMA lactylation. Consistently, AARS2 depletion significantly diminished the proliferative (Figure 4I–K; Figure S7A, Supporting Information) and metastatic (Figure 4L,M; Figure S7B–D, Supporting Information) effects of circTAX1BP1 in CRC cells. Collectively, these findings demonstrate that circTAX1BP1 recruits AARS2 and VIRMA to form a functional complex, leading to VIRMA lactylation and increased m^6^A levels, thereby promoting CRC cell proliferation and metastasis.

### 2.5 CAF‐Derived EV‐Encapsulated circTAX1BP1 Promotes CRC Progression by Lactylating VIRMA to Modulate SP1 m6A Modification

**Figure 5:**
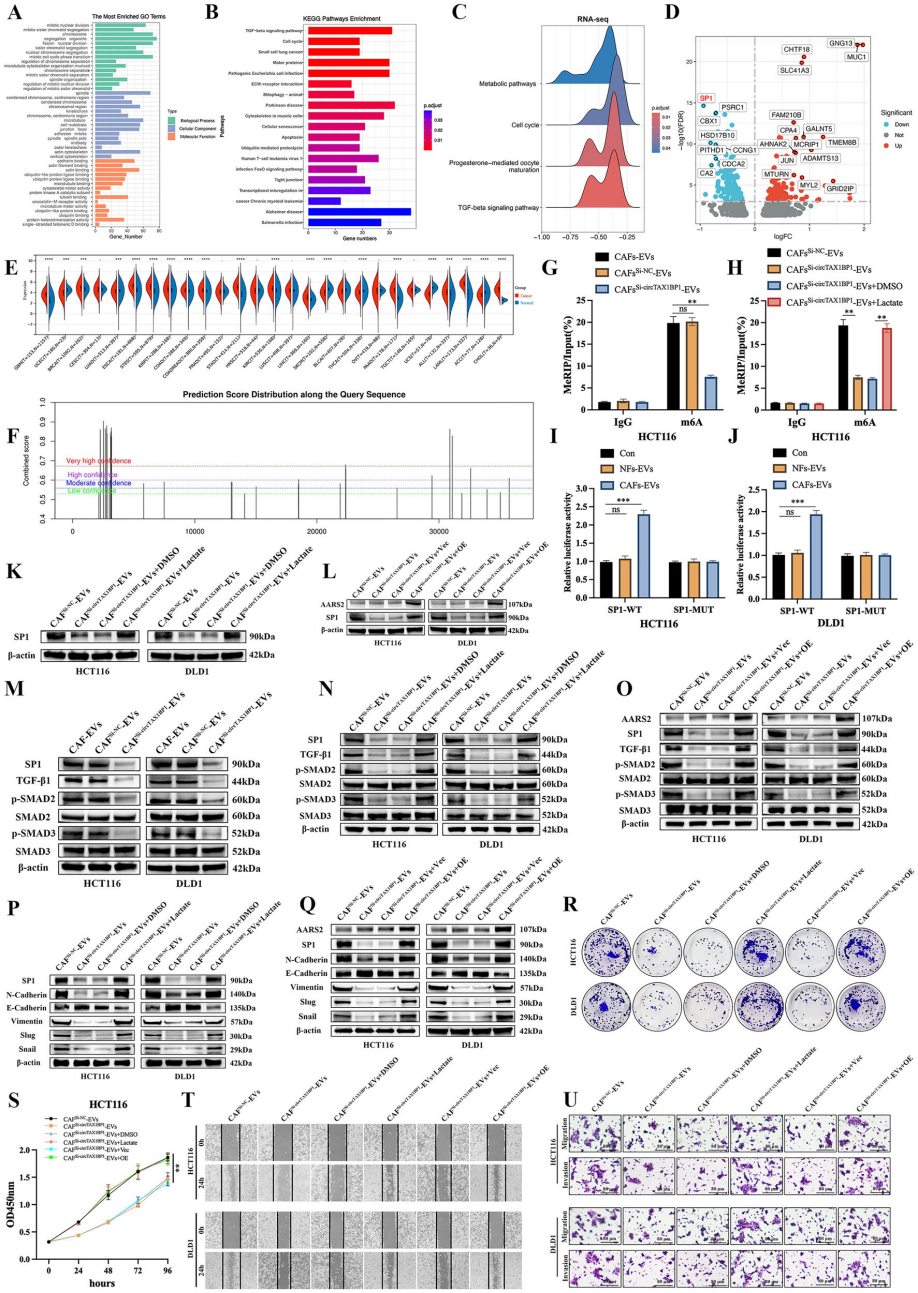
CAF‐derived EV‐packaged circTAX1BP1 promotes colorectal cancer (CRC) progression by lactating VIRMA to modulate SP1 m^6^A modification. A) Gene ontology of biological processes at the mRNA level. B,C) KEGG and GSEA enrichment analysis of the number of pathways at the mRNA levels. D) Volcano plots of upregulated and downregulated genes in circTAX1BP1 siRNA compared to those in siRNA con. E) SP1 expression in tumor and normal samples based on the TCGA and GTEx dataset. F) m^6^A modification of SP1 in the SRAMP prediction servers. G,H) MeRIP‐qPCR determinations for m^6^A enrichment on SP1 mRNA in HCT116 cells (*n* = 3). I,J) Relative activities of the WT and Mut luciferase reporters (*n* = 3). K) Relative protein levels of SP1 were determined using western blotting. L) Relative protein levels of SP1 and AARS2 were determined using western blotting. M–O) Relative protein levels of AARS2, SP1, TGF‐β1, p‐SMAD2, SMAD2, p‐SMAD3, and SMAD3 were determined using western blotting. P,Q) Relative protein levels of AARS2, SP1, N‐Cadherin, E‐Cadherin, Vimentin, Slug, and Snail were determined using western blotting. R) Proliferation of HCT116 and DLD1 cells was assessed using colony formation assays. S) Proliferation of HCT116 cells was assessed using CCK‐8 assays (*n* = 3). T) Migration of HCT116 and DLD1 cells was assessed using wound healing assays. U) Migration and invasion of HCT116 and DLD1 cells were assessed using transwell assays. Scale bar, 80 µm. The statistical difference was assessed through nonparametric Mann–Whitney *U*‐test in (E); and one‐way ANOVA followed by Dunnett tests in (G,I,J,S); and 2‐tailed Student's *t*‐test in (H). All data are presented as mean ± SD of experimental triplicates. ns, *P* > 0.05; **, *P* < 0.01; ***, *P* < 0.001.

To elucidate the molecular mechanisms underlying circTAX1BP1‐mediated effects, we performed RNA‐seq on CRC cells treated with CAF‐derived exosomes transfected with either circTAX1BP1 siRNA or a control siRNA. Gene Ontology analysis of the transcriptomic data revealed that circTAX1BP1 significantly affected biological processes related to cell proliferation, migration, and invasion, including “mitotic sister chromatid segregation”, “cell‐substrate junction”, “focal adhesion”, and “cadherin binding” (**Figure** **5**A). Kyoto Encyclopedia of Genes and Genomes pathway enrichment analysis further indicated that circTAX1BP1 knockdown in CAFs altered the TGF‐β signalling pathway (Figure 5B). In addition, gene set enrichment analysis confirmed the significant enrichment of the TGF‐β signalling pathway, prompting us to focus on this pathway for further investigation (Figure 5C; Figure S8A, Supporting Information). Afterward, we analyzed the expression of TGF‐β pathway‐enriched genes. In CRC cells, mRNA‐seq data revealed that circTAX1BP1 knockdown significantly decreased SP1 mRNA levels (Figure 5D). Pan‐cancer analysis demonstrated that SP1 expression is elevated in various cancer types (Figure 5E; Figure S8B, Supporting Information). Kaplan–Meier (KM) plot analysis revealed that high SP1 expression was associated with poorer OS in CRC (Figure S8C,D, Supporting Information). Moreover, analysis of the Clinical Proteomic Tumor Analysis Consortium and Gene Expression Omnibus databases indicated that SP1 expression was higher in colorectal LM tissues than in NATs or primary tumor tissues (Figure S8E–K, Supporting Information). Collectively, these findings suggest SP1 as a critical downstream target of circTAX1BP1‐mediated TGF‐β signalling, contributing to CRC progression.

Using the SRAMP online tool, we predicted that SP1 was highly likely to undergo m^6^A modification (Figure 5F). MeRIP‐qPCR analysis confirmed that SP1 m^6^A levels were significantly reduced in CRC cells treated with CAF‐derived exosomes transfected with circTAX1BP1 siRNA, compared to controls (Figure 5G; Figure S8L, Supporting Information). Notably, the addition of lactate reversed this reduction in SP1 m^6^A levels (Figure 5H; Figure S8M, Supporting Information). To identify the specific m^6^A modification sites on SP1, we mutated the predicted m^6^A sites in the SP1 5' UTR to generate a mutant SP1 construct (Figure S8N, Supporting Information). Luciferase reporter assays revealed that treatment of CAF‐derived exosomes increased luciferase activity in wild‐type SP1‐expressing CRC cells, whereas no significant change was observed in mutant SP1‐expressing cells (Figure 5I,J). Consistently, circTAX1BP1 knockdown in CAFs decreased SP1 protein levels in CRC cells (Figure S9A,B, Supporting Information). However, this reduction was rescued by lactate supplementation or AARS2 overexpression (Figure 5K,L; Figure S9C–E, Supporting Information). Collectively, these findings demonstrate that circTAX1BP1, in addition to lactylated VIRMA, regulates SP1 expression through m^6^A modification, thereby promoting CRC progression.

Furthermore, SP1 regulates the TGF‐β signalling pathway in cancer cells.^[^ ^31^ ^]^ Our data demonstrate that the low expression of circTAX1BP1 diminishes the upregulation of TGF‐β, p‐Smad2, p‐Smad3, and SP1 induced by CAF‐derived EVs (Figure 5M; Figure S9F,G, Supporting Information). Furthermore, in CRC cells, AARS2 overexpression or lactate supplementation reversed the effects of CAF‐derived EVs transfected with circTAX1BP1 siRNA, restoring TGF‐β, p‐Smad2, p‐Smad3, and SP1 expression (Figure 5N,O; Figure S9H–K, Supporting Information). The TGF‐β/Smad pathway plays a critical role in EMT.^[^ ^32^, ^33^ ^]^ Western blotting analysis confirmed that CAF‐derived EVs from circTAX1BP1‐knockdown CAFs reduced the expression of EMT‐associated markers and confirmed the involvement of TGF‐β/Smad signalling (Figure 5P,Q; Figure S10A–D, Supporting Information). Colony formation assays revealed that circTAX1BP1 knockdown in CAF‐derived EVs suppressed CRC cell proliferation. However, this inhibition was rescued by AARS2 overexpression or lactate supplementation (Figure 5R; Figure S10E, Supporting Information). CAF‐derived EVs transfected with circTAX1BP1 siRNA suppressed CRC cell proliferation (Figure 5S; Figure S10F, Supporting Information), migration, and invasion (Figure 5T,U; Figure S10G–I, Supporting Information). Notably, these effects were reversed by AARS2 overexpression or lactate supplementation. Collectively, our findings indicate that circTAX1BP1 in CAF‐derived EVs promotes CRC progression by regulating SP1 m^6^A modification through lactylated VIRMA, thereby activating the TGF‐β/Smad pathway and facilitating CRC progression.

### 2.6 TGF‐β Secreted by CRC Cells Target ITGA11+ myCAFs and Activates the TGF‐β Signalling Pathway

**Figure 6:**
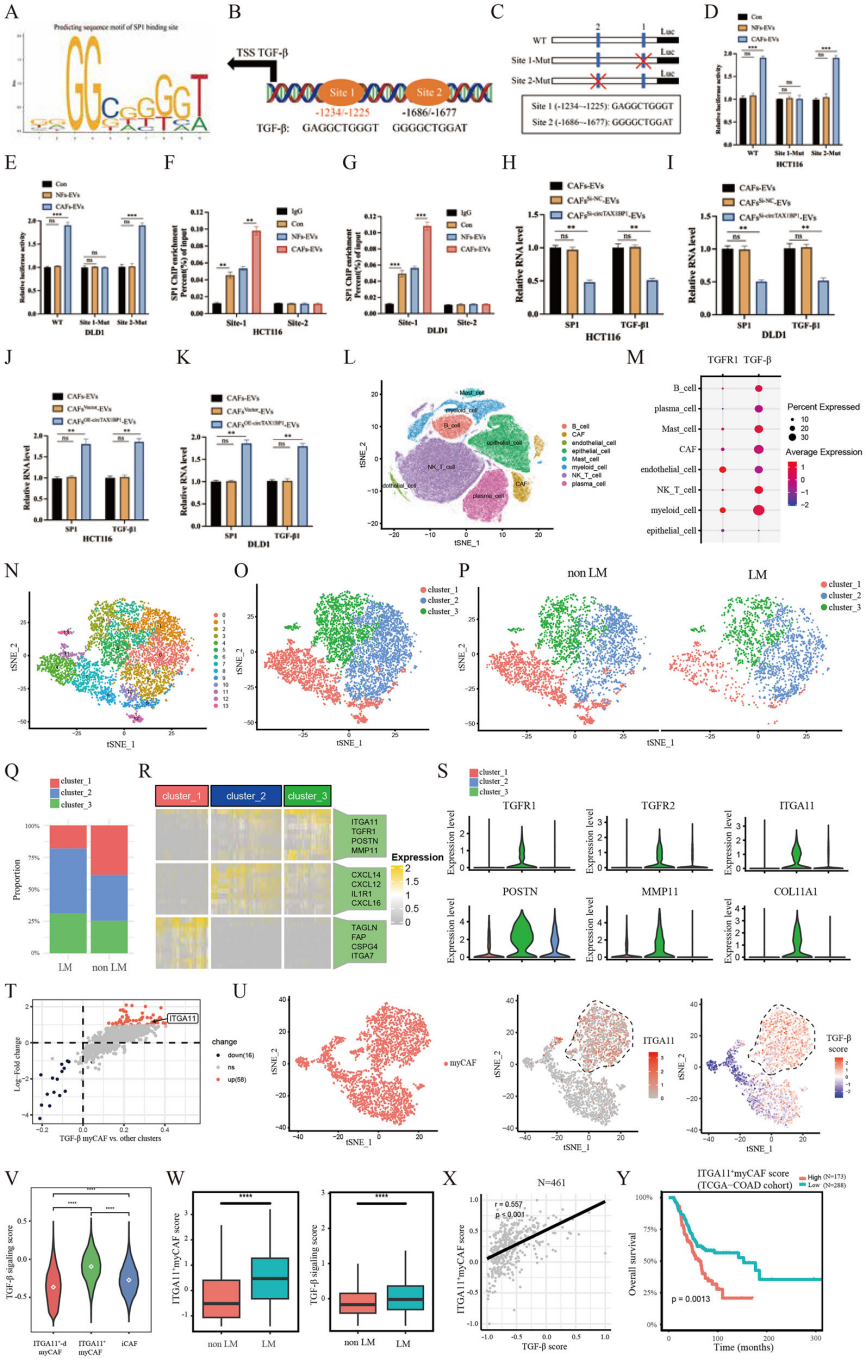
TGF‐β secreted by colorectal cancer (CRC) cells targets ITGA11^+^ myCAFs and activates the TGF‐β signalling pathway. A) Enriched motifs of SP1 binding sites predicted using JASPAR. B) Schematic model of SP1 binding sequences in the TGF‐β promoter region predicted using JASPAR and PROMO. C) Schematic plot of pGL3‐Basic vector containing wild‐type and two mutated motifs of the TGF‐β promoter. D,E) Relative activities of the WT and Mut luciferase reporters (*n* = 3). F,G) ChIP‐qPCR analysis of SP1‐enriched chromatin in HCT116 and DLD1 cells (*n* = 3). H–K) Relative TGF‐β1 and SP1 expressions were detected in HCT116 and DLD1 cells using qRT‐PCR (*n* = 3). L) t‐SNE plot showing the integration of single cells from metastatic and non‐metastatic CRC samples, partitioned into eight distinct cell clusters. M) TGFR1 and TGF‐β expression profiling across eight cell clusters reveals elevated expression in CAFs. N) Re‐clustering of CAFs partitioned this cellular compartment into 14 cell subclusters. O) t‐SNE plot showing clustering of 14 CAF populations into Clusters 1 (TGF‐β‐low myCAF), 2 (iCAF), and 3 (TGF‐β myCAF) based on expression profiles. P) Dimensionality reduction clustering of the three CAF subtypes in CRC with and without LM. Q) Proportions of the three CAF subtypes in CRC with and without LM, showing an elevated Cluster 3 proportion in CRC with LM. R) Heatmap of highly expressed genes in the three CAF subtypes. S) Violin plot shows the expression of the TGFR1, TGFR2, ITGA11, and collagen‐related genes in three CAF subtypes. T) Log2 fold‐change (*y*‐axis) and percentage difference (*x*‐axis) of gene expression (dots) between Cluster 3 and Clusters 1 and 2. U) t‐SNE plots of the myCAF cluster (left) showing heterogeneous in TGF‐β score (middle) and expression of ITGA11 (right). V) TGF‐β signalling pathway expression in the three CAF subtypes. W) In single‐cell samples, higher levels of ITGA11^+^ myCAF scores and TGF‐β signalling were observed in CRC with LM than in CRC without LM. X) Correlation between TGF‐β scores and ITGA11^+^ myCAF scores in TCGA‐COAD samples (*n* = 461). Y) Survival analysis of high (*n* = 173)‐ and low (*n *= 288)‐expression ITGA11^+^ myCAF score groups in TCGA‐COAD samples. The median expression level of ITGA11^+^ myCAF scores was taken as the cutoff value. *p*‐values were calculated by the log‐rank (Mantel–Cox). Spearman correlation analysis was used in (X). The statistical difference was assessed through one‐way ANOVA followed by Dunnett tests in (D,E,H–K,V); and 2‐tailed Student's *t‐*test in (F,G); and *χ* ^2^ test in (Q); and nonparametric Mann–Whitney *U*‐test in (W). Data are presented as mean ± SD of experimental triplicates. ns, *P* > 0.05; **, *P* < 0.01; ***, *P* < 0.001; ****, *P *< 0.0001.

SP1, acting as a transcription factor, enhances TGF‐β transcription, leading to an increase in its protein expression levels,^[^ ^34^ ^]^ consistent with our RNA‐seq results (Figure 5A,B). Therefore, we compared the promoter sequence of TGF‐β with the SP1‐binding motifs in the JASPAR database and predicted two potential SP1‐binding sites within the TGF‐β promoter (**Figure** **6**A,C). Subsequently, we used a dual‐luciferase reporter gene assay to validate these two potential binding sites. As expected, treatment of CRC cells with CAF‐derived exosomes significantly increased the activity of the TGF‐β promoter. However, this effect was eliminated after mutation of site 1 but not site 2 (Figure 6D,E), indicating that site 1 is required for SP1‐mediated regulation of TGF‐β. Furthermore, chromatin immunoprecipitation (ChIP) analysis revealed that treatment of CRC cells with exosomes mediating circTAX1BP1 (CRC^circTAX1BP1‐Evs^) led to a significant increase in SP1 enrichment at site 1 of the TGF‐β promoter, whereas no such increase was observed at site 2 (Figure 6F,G). Subsequently, we investigated the effect of SP1 on the changes in TGF‐β expression. After treatment of CRC cells with exosomes mediating circTAX1BP1 knockdown (CRC^si‐circTAX1BP1‐Evs^), SP1 and TGF‐β expression levels and CM concentration of TGF‐β in HCT116 and DLD1 cells were markedly decreased (Figure 6H,I; Figure S11A, Supporting Information). In contrast, the results were reversed when CRC cells were treated with exosomes mediating circTAX1BP1 overexpression (CRC^oe‐circTAX1BP1‐Evs^) (Figure 6J,K; Figure S11B, Supporting Information). These findings collectively suggest that exosomal circTAX1BP1 derived from CAFs mediates the transcriptional regulation of SP1 on TGF‐β and promotes TGF‐β extracellular secretion in CRC cells.

In consideration of heterogeneity in TME, we further investigated which component was the potential target of TGF‐β. We first integrated scRNA‐seq data from seven public datasets encompassing LM and non‐liver metastatic CRC samples. Following quality control, dimensionality reduction, and clustering (Figure S12A, Table S4, Supporting Information), all cells were classified into eight major cell types based on established lineage‐specific markers (Figure 6L). We initially examined the expression of TGF‐β pathway ligands and receptors, observing that fibroblasts exhibited the predominant expression of these components, indicating they likely constitute the most TGF‐β signalling‐sensitive and responsive cell population in the TME (Figure 6M; Figure S12B,C, Supporting Information). To further characterize TGF‐β signalling activity across different cell types, we performed gene set variation analysis (GSVA), which revealed that CAFs displayed the highest enrichment score for the TGF‐β signalling pathway among all identified cell populations (Figure S12D, Supporting Information).

We performed subclustering of CAFs based on transcriptomic signatures, revealing 14 transcriptomically distinct subclusters that coalesced into myCAFs and iCAFs (Figure 6N; Figure S12E,F, Supporting Information), consistent with previous studies.^[^ ^35^ ^]^ We found that the TGF‐β cluster was predominant in myCAFs groups, allowing us to distinguish TGF‐β‐high myCAFs from other myCAFs (Figure S12G,H, Supporting Information). Further, we redivided them into three clusters. Clusters 1, 2, and 3 were designated as TGF‐β‐low myCAFs, iCAFs, and TGF‐β myCAFs, respectively. These three major groups were obtained through unsupervised clustering of characteristic gene expression patterns (Figure 6O), among which the proportion of clusters 2 (iCAF) and clusters 3 (TGF‐β myCAF) were increased and Cluster 1(TGF‐β low myCAF) was decreased in CRLM compared to non‐liver metastasis (Figure 6P,Q), indicating a partial crucial myCAF subgroup contribute in CRLM. TGF‐β myCAF represented a myCAFs subpopulation characterized by concomitant expression of ITGA11, TGFR1/2, and ECM‐related gene (Figure 6R,S). Subsequently, we sought to identify surface markers that distinguish TGF‐β myCAFs subsets. Differentiated cluster‐signature RNA analysis indicated that ITGA11 was one of the most differentially expressed genes in TGF‐β myCAF (Figure 6T), which was reported to be specifically expressed in CAFs and upregulated by TGF‐β.^[^ ^36^ ^]^ Besides, ITGA11 is highly expressed in CAFs especially in ITGA11^+^ myCAFs compared to all other cell types (Figure S12I–K, Supporting Information). Moreover, we found TGF‐β signalling and ITGA11 expression co‐located in TGF‐β myCAF (Figure 6U), and we found ITGA11 expression in CAF was evaluated by TGF‐β and abolished by TGFR1 inhibitor (Figure S12L,M, Supporting Information). Considering subsequent FACS analysis, we termed TGF‐β myCAF and TGF‐β‐low myCAF as ITGA11^+^ myCAF and ITGA11^+^‐d myCAF. Notably, ITGA11^+^ myCAF exhibited the highest TGF‐β pathway activation scores (Figure 6V; Figure S12N, Supporting Information). Higher ITGA11^+^ myCAF and TGF‐β scores were demonstrated in CRLM compared to non‐metastatic samples (Figure 6W); this was indicative of not only elevated ligand/receptor expression but also enhanced pathway signalling capacity, likely attributable to increased receptor availability. Next, we applied the TGF‐β pathway score and ITGA11^+^ myCAF signature scores to the TCGA cohort; the results showed a significant positive correlation that underscores the association between TGF‐β pathway score and ITGA11^+^ myCAF (Figure 6X). As expected, patients with high ITGA11^+^ myCAF expression had reduced OS in the TCGA cohort (Figure 6Y; Figure S12O, Supporting Information). Collectively, these single‐cell sequencing data reinforce the critical roles of TGF‐β‐driven ITGA11^+^ myCAF and TGF‐β signalling in promoting CRLM.

### 2.7 TGF‐β Drives ITGA11+ myCAFs Upregulate EV‐Packed circTAX1BPP1 and ECM to Form a Feedback Loop

**Figure 7:**
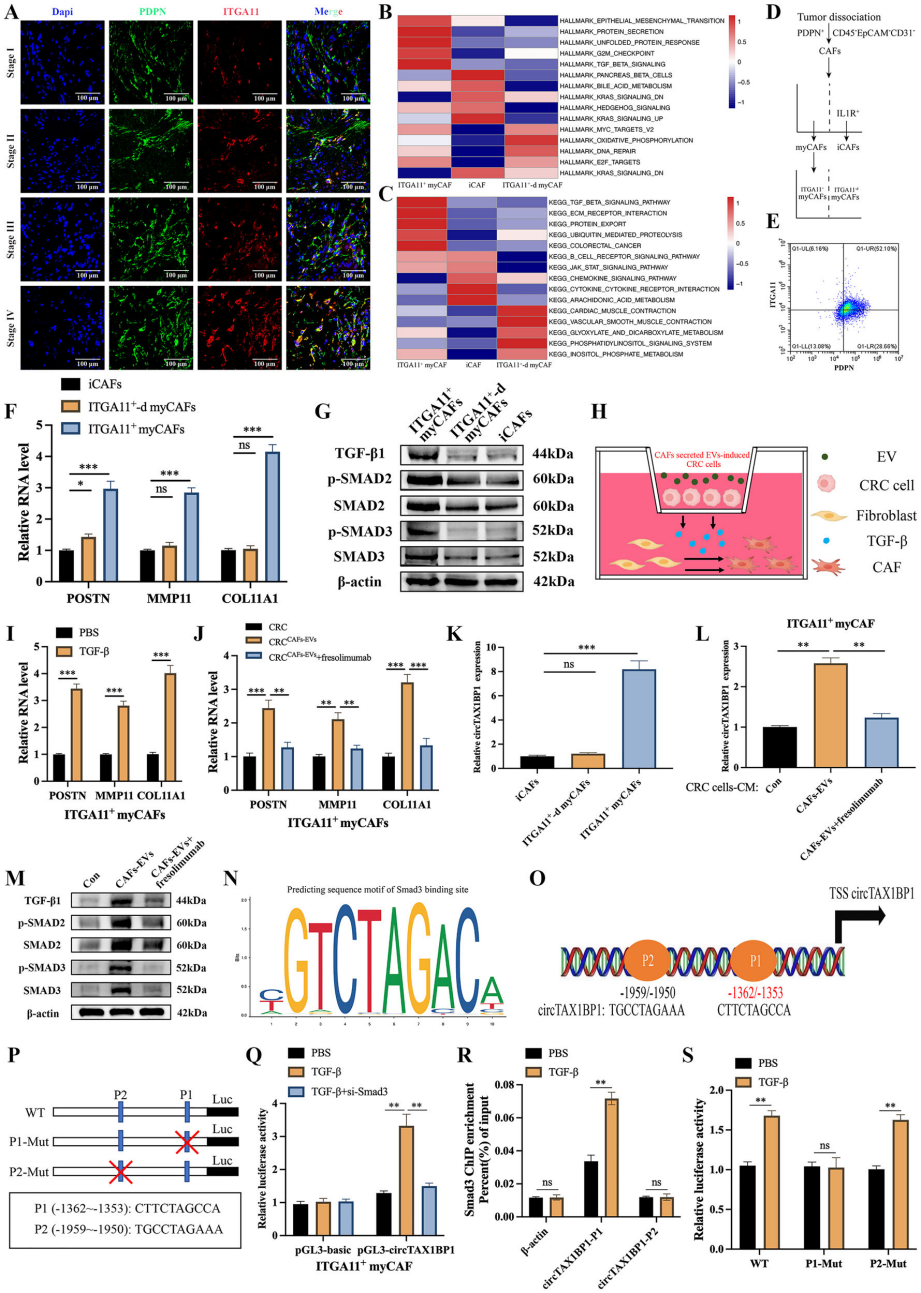
TGF‐β drives ITGA11^+^ myCAFs to upregulate EV‐packaged circTAX1BPP1 and ECM to form a feedback loop. A) CRC tissues were stained with PDPN and ITGA11 in immunofluorescence assays. Scale bar, 100 µm. B,C) Hallmark (B) and KEGG (C) pathway enrichment analysis of highly expressed pathways across three CAF subtypes. D,E) Schematic and representative flow plot of flow cytometric and flow‐sorting strategy of ITGA11^+^ myCAF. F) qRT‐PCR analysis of relative mRNA expression in iCAFs, ITGA11^+^‐d myCAFs, or ITGA11^+^ myCAFs (*n* = 3). G) Relative protein levels of TGF‐β1, p‐SMAD2, SMAD2, p‐SMAD3, and SMAD3 were determined using western blotting. H) Schematic presentation of the established co‐culture model of fibroblasts and EV‐induced CRC cells. I) qRT‐PCR analysis of relative mRNA expression in ITGA11^+^ myCAFs cultured with PBS or TGF‐β treatment (*n* = 3). J) qRT‐PCR analysis of relative mRNA expression in ITGA11^+^ myCAFs cultured with indicated CM from CRC cells with or without fresolimumab treatment (*n* = 3). K) qRT‐PCR analysis of circTAX1BP1 expression in iCAFs, ITGA11^+^‐d myCAFs, or ITGA11^+^ myCAFs (*n* = 3). L) qRT‐PCR analysis of circTAX1BP1 expression in ITGA11^+^ myCAFs cultured with indicated CM from CRC cells with or without fresolimumab treatment (*n* = 3). M) Relative protein levels of TGF‐β1, p‐SMAD2, SMAD2, p‐SMAD3, and SMAD3 were determined using western blotting. N) Enriched motifs of smad3 binding sites predicted by JASPAR. O) Schematic model of smad3 binding sequences in the circTAX1BP1 promoter region predicted by JASPAR and PROMO. P) Schematic plot of pGL3‐Basic vector containing wild‐type and mutated two motifs of the circTAX1BP1 promoter. Q) Relative transcriptional activity of circTAX1BP1 in TGF‐β‐treated ITGA11^+^ myCAFs with or without smad3 silencing (*n* = 3). R) ChIP‐qPCR analysis of smad3‐enriched chromatin in ITGA11^+^ myCAFs (*n* = 3). S) Relative activities of the WT and Mut luciferase reporters (*n* = 3). The statistical difference was assessed through one‐way ANOVA followed by Dunnett tests in (F,J–L,Q); and 2‐tailed Student's *t*‐test in (I,R,S). All data are presented as mean ± SD of experimental triplicates. ns, *P* > 0.05; *, *P* < 0.05; **, *P* < 0.01; ***, *P* < 0.001.

Following the identification of the ITGA11^+^ myCAF subset, we found that the infiltration of ITGA11^+^ myCAFs increased with CRC pathological stage (**Figure** **7**A). In addition, GSVA revealed that the ITGA11^+^ myCAF expression group exhibited significant upregulation of ECM besides TGF‐β signalling (Figure 7B,C). Therefore, we sorted ITGA11^+^ myCAFs from primary CAFs (Figure 7D,E), which further validated that ITGA11^+^ myCAF had a high expression of ECM‐related gene (COL11A1, MMP11, POSTON) and TGF‐β signalling compared to iCAF or ITGA11^+^‐d myCAF (Figure 7F,G; Figure S13A, Supporting Information). In addition, ECM‐ related genes were upregulated after TGF‐β signalling or co‐culture with CRC^circTAX1BP1‐Evs^ (Figure 7H–J), which indicated that TGF‐β secreted by CRC cells may be a driving factor in ITGA11^+^ myCAF activation.

Accumulating evidence suggests that CAFs stimulated by cancer cells engage in reciprocal regulation of the invasive biological behaviors of cancer cells, thereby enhancing tumor metastasis.^[^ ^5^ ^]^ Consequently, we suspected that ITGA11^+^ myCAFs were the main source of EV‐packaged circTAXIBP1 in primary CAFs. As expected, intercellular circTAX1BP1 and EV‐packaged circTAX1BP1 were significantly higher in ITGA11^+^ myCAF compared with iCAF or ITGA11^+^‐d myCAF (Figure 7K; Figure S13B, Supporting Information). Our results revealed that following co‐culturing with CRC^circTAX1BP1‐Evs^, the expression of circTAX1BP1 and EV‐packaged circTAX1BP1 in ITGA11^+^ myCAF was significantly upregulated and abolished by TGF‐β inhibitor GC‐1008 (fresolimumab), consistent with TGF‐β signalling (Figure 7L,M; Figure S13C,D, Supporting Information). However, circTAX1BP1 level in iCAF and ITGA11^+^‐d myCAF was not affected by co‐culturing (Figure S13E, Supporting Information), indicating the presence of a potential positive feedback loop between EV‐packaged circTAX1BP1 from ITGA11^+^ myCAFs and TGF‐β from CRC cells. Given that cancer cells can induce specific transcription of circRNAs by modulating the activation of numerous transcription factors,^[^ ^37^ ^]^ we evaluated potential transcription factors that interact with the circTAX1BP1 promoter and may be involved in the upregulation of circTAX1BP1 in ITGA11^+^ myCAF. As demonstrated in Figure 7N–P, Smad3 was identified as a potential transcription factor binding to the circTAX1BP1 promoter, which was highly activated in ITGA11^+^ myCAFs. Knockdown of Smad3 markedly attenuated the promotion effect of TGF‐β on the transcriptional activity of circTAX1BP1 in ITGA11^+^ myCAFs, suggesting that Smad3 is necessary for the induction of circTAX1BP1 transcription by TGF‐β (Figure 7Q; Figure S13F, Supporting Information). Furthermore, ChIP analysis revealed that TGF‐β significantly increased the enrichment of Smad3 in the −1351 to −1362 bp region (designated as P1) of the circTAX1BP1 promoter but not at another predicted binding site located in the −1950 to −1959 bp region (designated as P2) (Figure 7R). Mutation of the P1 region in the circTAX1BP1 promoter significantly diminished the promotion effect of TGF‐β on the transcriptional activity of circTAX1BP1 in ITGA11^+^ myCAFs, whereas mutation of the P2 region had a minimal effect (Figure 7S). These findings indicate that TGF‐β mediates the direct interaction of Smad3 with the P1 region of the circTAX1BP1 promoter in ITGA11^+^ myCAFs, thereby activating its transcription. More importantly, single‐cell sequencing data supported that TAX1BP1 is highly expressed exclusively in clusters 3 (Figure S14A, Supporting Information), while lactylation level, VIRMA and SP1 were highly expressed in tumor cells (Figure S14B–E, Supporting Information). These results suggest that the SP1‐TGF‐β‐Smad3 positive feedback loop between CRC cells and ITGA11^+^ myCAFs, induced by circTAX1BP1, promotes CRC progression.

### 2.8 Clinical Relevance of circTAX1BP1‐Induced Positive Feedback Loop in CRC Liver Metastasis

**Figure 8:**
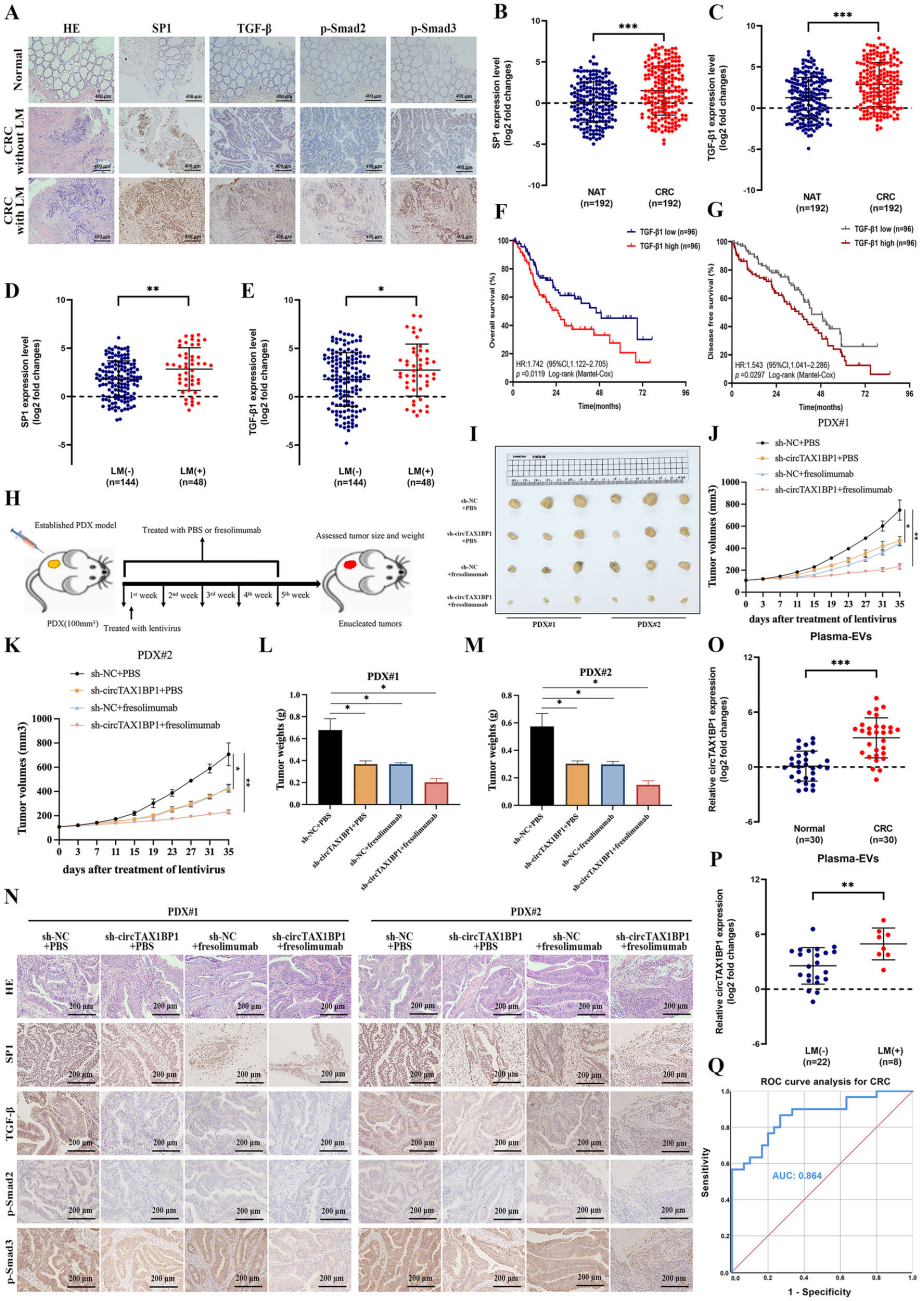
Clinical relevance of the circTAX1BP1‐induced positive feedback loop in colorectal cancer (CRC) liver metastasis. A) IHC results of SP1, TGF‐β1, p‐Smad2, and p‐Smad3 in CRC and normal tissues in samples from CRC patients with or without LM. Scale bar, 400 µm. B–E) qRT‐PCR analysis of SP1 and TGF‐β1 expression between CRC tissues and NATs (*n* = 192) or between LM‐negative (*n* = 144) and LM‐positive (*n* = 48) CRC tissues. F,G) Kaplan–Meier curves of the OS and DFS of patients with CRC with low vs. high TGF‐β1 expression levels. The cutoff value is the median. *p*‐values were calculated by the log‐rank (Mantel–Cox) test. H) Schematic illustration of the establishment of the PDX model. I) Images of PDX tumors harvested from the mice. J,K) Tumor volume summary for mice, measured every 4 days (*n* = 3). L,M) Mice were examined for tumor weights (*n* = 3). N) IHC staining for SP1, TGF‐β, p‐Smad2, and p‐Smad3 was measured in PDX tumors harvested from the mice. Scale bar, 200 µm. O,P) qRT‐PCR analysis of circTAX1BP1 expression in plasma EVs from patients with CRC (*n* = 30) and healthy controls (*n* = 30) or CRC patients with (*n* = 8) or without (*n* = 22) LM. Q) ROC curves for the efficiency of plasma EV‐mediated circTAX1BP1 in diagnosing CRC. The statistical difference was assessed through nonparametric Mann–Whitney *U*‐test in (B–E,O,P); and one‐way ANOVA followed by Dunnett tests in (J–M). All data are presented as mean ± SD of experimental triplicates. *, *P* < 0.05; **, *P* < 0.01; ***, *P* < 0.001.

Given that TGF‐β plays a pivotal regulatory role in the circTAX1BP1‐induced positive feedback loop, we assessed the clinical relevance of TGF‐β in patients with CRC and liver metastasis. Immunohistochemistry (IHC) analysis revealed significantly upregulated expressions of SP1, TGF‐β, p‐Smad2, and p‐Smad3 in patients with CRC and liver metastasis compared with those with CRC without liver metastasis (**Figure** **8**A; Figure S15A, Supporting Information). qRT‐PCR analysis demonstrated that TGF‐β and SP1 expression levels were higher in CRC tissues than in their paired adjacent non‐tumor tissues (*n* = 192) (Figure 8B,C). Based on circTAX1BP1 in 192 CRC tissues, a positive correlation between circTAX1BP1, SP1, and TGF‐β expression was observed in CRC tissues (Figure S15B–D, Supporting Information). Notably, TGF‐β and SP1 expressions were markedly elevated in LM CRC tissues compared to non‐LM CRC tissues (Figure 8D,E). Furthermore, TGF‐β and SP1 overexpression were positively correlated with shorter OS and DFS in patients with CRC (Figure 8F,G; Figure S15E,F, Supporting Information).

Furthermore, we evaluated the clinical application potential of circTAX1BP1 and fresolimumab in the treatment of CRC accompanied with liver metastasis (Figure 8H). The results demonstrated that in patient‐derived xenograft (PDX) models established using CRC with liver metastasis tissues, reducing the expression of circTAX1BP1 and administering fresolimumab were effective in inhibiting tumor growth (Figure 8I–M). Moreover, in the PDX models, circTAX1BP1 silencing and fresolimumab treatment significantly suppressed the expression levels of SP1, TGF‐β, p‐Smad2, and p‐Smad3 (Figure 8N; Figure S15G,H, Supporting Information). These findings suggest that circTAX1BP1 and fresolimumab hold potential clinical value in the treatment of patients with CRC accompanied with liver metastasis.

Given that EV‐mediated circRNAs have emerged as promising biomarkers for the early diagnosis of cancer, we assessed the clinical relevance of EV‐mediated circTAX1BP1 in patients with CRC and liver metastasis. To assess the levels of circTAX1BP1 in plasma EVs from patients with CRC, plasma EVs were collected from LM and non‐LM patients with CRC. Plasma EVs from patients with CRC were characterized using electron microscopy and particle size analysis (Figure S15I,J, Supporting Information). qRT‐PCR analysis revealed that circTAX1BP1 expression in plasma‐derived EVs was significantly upregulated in patients with CRC compared with healthy controls (Figure 8O). Furthermore, circTAX1BP1 expression in plasma EVs was higher in patients with CRC with liver metastasis than in those without liver metastasis (Figure 8P). Notably, plasma EV‐mediated circTAX1BP1 effectively discriminated patients with CRC from healthy controls, with an area under the curve of 0.864 (95% confidence intervalCI]: 0.773–0.956) (Figure 8Q). These findings suggest that EV‐packaged circTAX1BP1 is a potential therapeutic target and diagnostic biomarker for LM‐positive CRC.

## 3 Discussion

Liver metastasis is the most common metastatic site of CRC, and synchronous and metachronous liver metastasis account for approximately half of the cases with CRC.^[^ ^38^ ^]^ The distinct TME provides the necessary support for CRC cells to develop CRLM.^[^ ^3^ ^]^ Increasing evidence indicates that different cell components jointly contribute to the progression and metastasis of CRC through EVs via a unique mechanism.^[^ ^39^, ^40^ ^]^ In our study, using CRC patient cohorts, cell culture experiments, and a subcutaneous tumor model, we found that circTAX1BP1 is abundant in CAF‐derived EVs and confirmed that the level of EV‐packaged circTAX1BP1 is related to the pathological stage and prognosis of patients with CRC. Furthermore, we demonstrated that CAF‐derived EV‐packaged circTAX1BP1 was internalized by CRC cells and promoted the transcription and paracrine signalling of TGF‐β; this maintained the TGF‐β signalling activation of ITAG11^+^ myCAFs and enhanced EV‐packaged circTAX1BP1 secretion to promote CRLM. Notably, our data confirmed that EV‐packaged circTAX1BP1 can be applied as a potential serum biomarker for the diagnosis of CRLM, demonstrating the utility of EVs in the field of tumor diagnosis and treatment.^[^ ^41^, ^42^ ^]^

Our study revealed a novel finding that CAF‐derived EV‐packaged circTAX1BP1 can directly bind to VIRMA in CRC cells. CircRNA can function as a scaffold to regulate the ubiquitination, SUMOylation, acetylation, and palmitylation of target proteins.^[^ ^43^, ^44^, ^45^, ^46^ ^]^ The discovery of lactylation modification is of landmark significance.^[^ ^47^ ^]^ As an important PTM, it allows a better understanding of the relationship between tumor metabolism and epigenetics and accelerates tumor metastasis and drug resistance; however, the limited research in this field warrants further expansion.^[^ ^48^, ^49^ ^]^ Notably, CAF‐derived EV‐packaged circTAX1BP1 regulated the lactylation of VIRMA in CRC cells. To the best of our knowledge, this is the first report of lactylation modification of lysine 1713 of VIRMA, which was confirmed by the construction K1713R mutation. Furthermore, we confirmed that circTAX1BP1 promoted the lactylation of VIRMA by recruiting the lactyltransferase AARS2, which was demonstrated to mediate protein lysine lacylation.^[^ ^30^ ^]^ The maintenance of lactylation balance depends on lactate transferase (KAT8, P300, or TIP60) and delactylases (HADC1–3),^[^ ^50^, ^51^, ^52^ ^]^ and our study illustrated that circTAX1BP1 plays a role in this process, providing new insights into the field of RNA‐regulated protein lactylation modification.

As an m^6^A transferase, VIRMA, similar to METTL3/METTL14/WTAP, promotes the m^6^A modification of target RNA and increases or decreases its stability.^[^ ^53^ ^]^ Although the known target RNAs of VIRMA are limited, its cancer‐promoting function has been reported in various malignant tumors.^[^ ^54^, ^55^, ^56^ ^]^ In this study, using RNA‐seq and MeRIP, we confirmed that the downstream target mRNA of lactylation VIRMA is SP1. As an important pro‐cancer transcription factor, we used JASPAR to predict and verify the transcriptional activity of SP1 in activating TGF‐β, which revealed the important role of the VIRMA‐SP1‐TGF‐β axis in CRLM. Moreover, the VIRMA‐SP1‐TGF‐β axis was confirmed to be associated with poor prognosis and liver metastasis in CRC clinical samples, which enriched the theory in the field of VIRMA‐mediated epigenetic regulation in CRLM.

**Figure 9:**
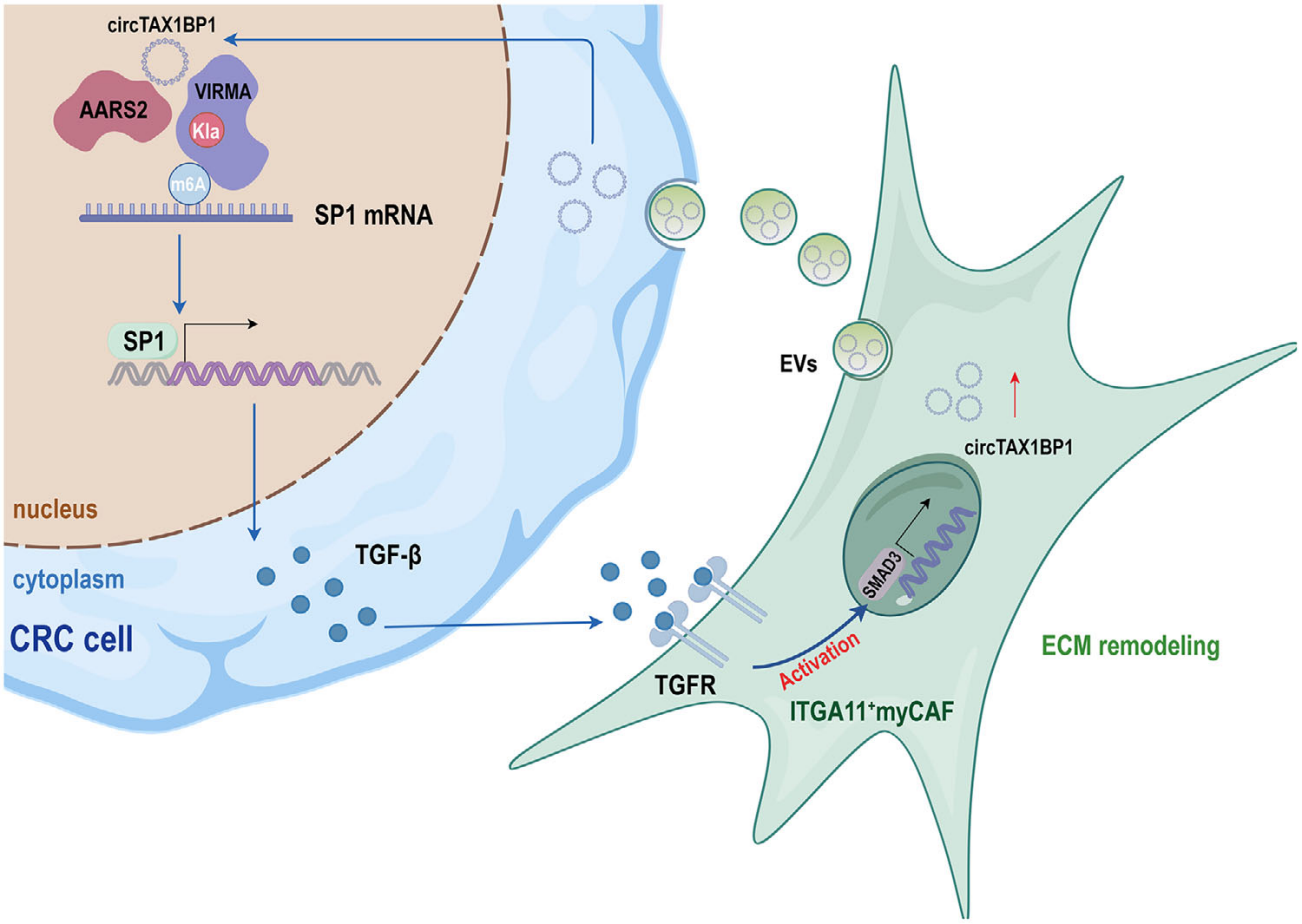
EV‐packaged circTAX1BP1 from ITGA11^+^ myCAFs regulates RNA m^6^A modification through lactylation of VIRMA.

Accumulating evidence shows that TGF‐β‐activated CAFs in CRC help to create the premetastatic microenvironment.^[^ ^57^, ^58^ ^]^ Patients with “mesenchymal” CRC have the worst prognosis and TME characterized by significant CAFs infiltration and elevated TGF‐β level.^[^ ^59^ ^]^ Kojima et al. discovered that CAFs derive an autoactivation mechanism through autocrine TGF‐β to maintain their characteristics,^[^ ^60^ ^]^ emphasizing that TGF‐β is a crucial factor in inducing CAF activation and tumor metastasis. Previous studies have proved that TGF‐β can activate fibroblasts to transform into myCAFs.^[^ ^61^ ^]^ The emergence of single‐cell sequencing technology provides a unique opportunity to explore more heterogeneity of CAFs in TME. Dominguez et al. discovered that LRRC15^+^ myCAFs in pancreatic cancer are activated by TGF‐β.^[^ ^62^ ^]^ Yann Kieffer found that LAMP5^+^ myCAF in breast cancer is mainly activated by TGF‐β,^[^ ^63^ ^]^ whereas the TME subtype that specifically accepts TGF‐β in CRC has not been fully clarified, which limits antitumor therapy via TGF‐β targeting. Our study has confirmed that TGF‐β mainly targets the unique subpopulation of ITGA11^+^ myCAF in CRLM. In addition, we demonstrated that the ITGA11^+^ myCAF subgroup produced a cascade signalling under TGF‐β/TGFR activation and highly expresses ECM‐related genes such as COL11A1 and MMP11, assisting tumor cells in initiating the metastatic program. Although Fang et al. revealed the importance of myCAFs in impaired NK cells to promote CRLM,^[^ ^64^ ^]^ our study identified a more accurate subgroup, ITGA11^+^ myCAFs, as an abundant and crucial subgroup that shows specific TGF‐β activation through integration and analysis of existing data. Our findings revealed that the infiltration of ITGA11^+^ myCAFs correlated to the CRLM. Notably, TGF‐β signalling promoted the transcription of intracellular circTAX1BP1 and the enrichment of circTAX1BP1 in EVs. EV‐packaged circTAX1BP1 mediates paracrine TGF‐β signalling in CRC cells, thereby forming a positive feedback loop. This positive feedback loop provides a continuous source of high levels of TGF‐β infiltration in TME and maintains the characteristics of highly enriched ITGA11^+^ myCAFs in TME. Therefore, ITGA11^+^ myCAFs play a dual role in indirectly inducing TGF‐β and ECM remodelling through EV‐packaged circTAX1BP1, providing a potential premetastatic microenvironment (**Figure** **9**).

Our study has some limitations that should be addressed in future research. First, the key molecules involved in the transport or uptake of EV‐packed circTAX1BP1 were not explored. Second, we found that serum EV‐packaged circTAX1BP1 can be used as a potential biomarker for the diagnosis of CRC; however, whether it can be used to monitor the recurrence of liver metastasis after surgery remains unknown, and more cohort studies are needed for confirmation.

## 4 Conclusion

Taken together, our study elucidates a novel mechanism by which ITGA11^+^ myCAF‐derived EV‐packaged circTAX1BP1 recruits AARS2 to promote the lactylation modification of VIRMA at K1713 in colorectal tumor cells, enhances VIMRA‐K1713la‐dependent m^6^A modification of SP1 mRNA, and increases the transcriptional activation and paracrine signalling of TGF‐β in tumor cells. TGF‐β maintains the stemness of ITGA11^+^ myCAFs and promotes the secretion of exosomal circTAX1BP1, forming a positive feedback loop. Our data provide reliable and comprehensive evidence to support the target combination CAF‐derived EV‐packaged circTAX1BP1 and TGF‐β as an optimal therapeutic strategy for CRLM.

## 5 Experimental Section

Please find the complete Experimental Section in File S1 (Supporting Information).

### CRC Cell Lines and Cell Culture

The uncontaminated colorectal cancer cell lines HCT116 (American Type Culture CollectionATCC], Manassas, VA, USA; Cat# CCL‐247, RRID: CVCL_0291) and DLD1 (ATCC; Cat# CCL‐221, RRID: CVCL_0248) were obtained from the ATCC and maintained in the Tumor Laboratory of the Department of Gastrointestinal Surgery, Sun Yat‐sen Memorial Hospital, Sun Yat‐sen University. Normal fibroblasts (NFs) and cancer‐associated fibroblasts (CAFs) were isolated from fresh human normal peritumoral tissues and primary tumor tissues, respectively. All cell lines were confirmed to be free of mycoplasma contamination by routine testing prior to experimental use. CRC cells were cultured in Dulbecco's Modified Eagle Medium (DMEM; Gibco, Carlsbad, CA, USA) supplemented with 10% fetal bovine serum (FBS; Invitrogen, Carlsbad, CA, USA). The isolated CAFs and NFs were cultured in Fibroblast medium (ScienCell Research Laboratories, Carlsbad, CA, USA) supplemented with 2.5% FBS and 1% growth factor. The culture conditions were set at 37 °C in a humidified atmosphere containing 5% CO_2_. All experiments were carried out during the logarithmic growth phase of the cells.

### Statistical Analysis

All statistical analyses were performed using GraphPad Prism 10.0 (GraphPad Software, Inc., CA, USA). The data were derived from at least three independent experiments. A *P*‐value of less than 0.05 was considered statistically significant. For measurement data, results are presented as the mean ± standard deviation (SD). The statistical significance was analyzed by 2‐tailed Student's *t‐*test, One‐way ANOVA followed by Dunnett's tests, *χ* ^2^ test, nonparametric Mann–Whitney U test as indicated. The overall survival (OS) and disease‐free survival (DFS) of patients were analyzed using the Kaplan–Meier method. Correlations between the groups were analyzed using Spearman's correlation. The following notation is used to indicate statistical significance: *P < 0.05; **P < 0.01; ***P < 0.001; ****P < 0.0001. Statistical details of each experiment were described in the relevant figure legends.

### Ethics Approval and Consent to Participate

This study was approved by the Ethics Committee of Sun Yat‐Sen Memorial Hospital (Approval No. SYSKY‐2025‐329‐01). All patients provided informed consent for the collection of tissue specimens. The animal studies were approved by the Laboratory Animal Center of Sun Yat‐Sen University and the Sun Yat‐Sen Memorial Hospital of Sun Yat‐Sen University (Approval No. SYSU‐IACUC‐2025‐000718; SYSU‐IACUC‐2025‐000719). Registry and the Registration No. of the study/trial: N/A.

## Conflict of Interest

The authors declare no conflict of interest.

## Author Contributions

J.T., J.Y., and D.H. contributed equally to this work. N.T., J.H.Y., and D.H. were responsible for performing experiments, acquisition of data, and writing the manuscript. Y.Q.X. and D.M.L. checked and analyzed the data. B.Y. and J.T.Z. collected the clinical samples and clinical data. F.Z., Y.C., and S.H.L. provided the technical support. S.N.Z., F.H.H., and G.Y.Z. were responsible for designing the experiments. The authors read and approved the final manuscript.

## Supporting information



Supporting Information

Supporting Information

Supporting Information

Supporting Information

Supporting Information

Supporting Information

Supporting Information

Supporting Information

Supporting Information

## Data Availability

The data that support the findings of this study are available from the corresponding author upon reasonable request.
