# Supplementary material for: Extracellular Vesicle‐Packaged circTAX1BP1 from Cancer‐Associated Fibroblasts Regulates RNA m6A Modification through Lactylation of VIRMA in Colorectal Cancer Cells
Source: Adv Sci (Weinh). 2025 Sep 29;12(47):e14008. doi: 10.1002/advs.202514008 (PMC12713077; doi:10.1002/advs.202514008)
Supplement: Supplementary file 1 — Supporting Information [file ADVS-12-e14008-s006.docx]

Methods and materials

1. Patients and human samples

In this study, tissue specimens were collected from colorectal cancer (CRC) patients at Sun Yat-sen Memorial Hospital, Sun Yat-sen University. The cohort comprised 144 patients with CRC who did not develop liver metastases and 48 patients with CRC who had liver metastases. Inclusion Criteria: (1) Definitive pathological diagnosis of colorectal cancer. (2) Absence of preoperative radiotherapy or chemotherapy. (3) No history of immune-related disorders. (4) Availability of complete clinical data. During the surgical procedures, tumor tissues were obtained. The tissues were then fixed in formalin, rapidly frozen in liquid nitrogen, and stored at -80°C. Colorectal cancer staging was performed according to the 8th edition of the American Joint Committee on Cancer (AJCC) TNM classification system. The primary tumor (T) was categorized as Tis (carcinoma in situ confined to mucosa), T1 (invasion into submucosa), T2 (invasion into muscularis propria), T3 (penetration through muscularis propria into pericolorectal tissues or non-peritonealized pericolic/perirectal tissues), T4a (perforation of visceral peritoneum), or T4b (direct invasion into adjacent organs/structures). Regional lymph node involvement (N) was classified as N0 (no metastasis), N1 (1-3 metastatic nodes including N1a for 1 node, N1b for 2-3 nodes, and N1c for tumor deposits without nodal metastasis), or N2 (≥4 metastatic nodes including N2a for 4-6 nodes and N2b for ≥7 nodes). Distant metastasis (M) was defined as M0 (absent) or M1 (present, with M1a for single-organ metastasis and M1b for multi-organ/peritoneal metastasis). Clinical stage grouping followed AJCC criteria: Stage 0 (TisN0M0); Stage I (T1-2N0M0); Stage II (T3-4N0M0); Stage III (any TN1-2M0); and Stage IV (any T any N M1). Staging was supported by multimodal diagnostics including colonoscopy with biopsy for initial diagnosis, contrast-enhanced CT/MRI for local and distant metastasis assessment, and pathological examination of surgical specimens for definitive TNM determination. Two independent pathologists confirmed the tumor nature of all the collected tissue specimens. Clinical tumor characteristics were gathered from clinical reports. Information on sample-related data, such as gender and age, was obtained through face-to-face interviews with the patients. This study was approved by the Medical Ethics Committee of Sun Yat-sen Memorial Hospital, Sun Yat-sen University (Approval No: SYSKY-2025-329-01).

1. RNA isolation and RNAseq

RNA was extracted from frozen human tissues using TRIzol reagent (Invitrogen, Carlsbad, CA, USA) strictly in accordance with the manufacturer's protocol. For RNA-seq analysis, 15 samples were sequenced on the Illumina HiSeq platform (Aksomics, Shanghai, China). On average, each sample yielded approximately 14.51 Gb of bases. Total RNA samples were subjected to oligo dT enrichment to remove rRNA. The libraries were then prepared using the KAPA Strand RNA-seq Library Prep Kit (Illumina, Shanghai, China). During the library construction process, the dUTP method was employed in combination with a high-fidelity PCR polymerase to synthesize double-stranded cDNA, thereby ensuring the strand-specificity of the final RNA-seq libraries. The constructed libraries were evaluated using the Agilent 2100 Bioanalyzer and quantified by real-time quantitative PCR (qRT-PCR). The pooled libraries of different samples were sequenced on the Illumina NovaSeq 6000 sequencing instrument. Image processing and base calling were performed using the Solexa Pipeline version 1.8 (an offline base caller software, version 1.8). STAR software was used to align the reads to the reference genome (GRCh37). CIRCexplorer2 was employed to detect spliced reads. The read counts and differential expression were analyzed using the edgeR package in R. Correlation analysis based on gene expression levels, as well as other data mining analyses such as clustering of differentially expressed genes, were conducted using Python, R, and Shell scripts.

1. Quantitative real-time PCR (qRT-PCR)

Total RNA was extracted from CRC tissues and cell lines using TRIzol reagent (Invitrogen). HiScript II Q Select RT SuperMix (Vazyme, Nanjing, China) was employed for cDNA synthesis to analyze circular RNAs (circRNAs) and messenger RNAs (mRNAs). Subsequently, PCR amplification was performed using ChamQ SYBR qPCR Master Mix (Vazyme). The analysis was carried out on the LightCycler 96 system (Roche, Mannheim, Germany). GAPDH or U6 served as internal controls for the detection of circRNAs and mRNAs. The relative expression levels were determined using the 2⁻^ΔCt^ or 2⁻^ΔΔCt^ method. All experiments were repeated three times. The primers used are listed in Supplementary Table 5.

1. Bioinformatics analysis

The sequence of circTAX1BP1 was obtained from CircBase (http://www.circbase.org/). The catRAPID online platform (http://service.tartaglialab.com/page/catrapid_group/) was utilized to identify potential protein-binding sites on circTAX1BP1. AlphaFold3 (https://alphafoldserver.com/) was employed to predict the molecular docking structures between circTAX1BP1 and the VIRMA and AARS2 proteins. Subsequently, the predicted structures were visualized using PyMOL. UALCAN online website (https://ualcan.path.uab.edu/index.html/), leveraging data from the TCGA (https://www.cancer.gov/ccg/research/genome-sequencing/tcga/) and CPTAC (https://proteomic-s.cancer.gov/programs/cptac/) databases, was used to analyze the gene and protein expression levels of SP1 across various tumor types. Kaplan-Meier Plotter (https://kmplot.com/analysis/inde-x.php?p=home/) was employed to conduct survival analysis. The SRAMP online website (http://www.cuilab.cn/m6asiteapp/old/) was utilized to predict potential m6A modification sites on the SP1 gene. The JASPAR online platform (https://jaspar.elixir.no/) was used to predict the binding sites of transcription factors on the promoter region of the target gene.

1. RNase R treatment

Total RNA (2 μg) was incubated at 37°C for 30 minutes, either in the presence or absence of RNase R (5 U*μg^-1^, Epicentre Technologies, Madison, WI, USA). Subsequently, the RNA samples were purified using the RNeasy MinElute Cleanup Kit (Qiagen, Germantown, MD, USA). Following purification, qRT-PCR analysis was performed.

1. Actinomycin D treatment

Cells were seeded in 6-well plates and cultured until they reached 70%-80% confluence. Actinomycin D (Sigma, Saint Louis, MO, USA) was added to the culture medium at a final concentration of 2 μg/ml. After the actinomycin D treatment, RNA was extracted at 0, 4, 8, 12, and 24 h respectively. The expression levels of circRNA and mRNA were then detected using qRT-PCR.

1. Immunofluorescence staining

For paraffin-embedded tissue sections, after dewaxing and hydration, heat-induced antigen retrieval was performed using Tris-EDTA buffer (pH 9.0). The sections were then incubated with PAN-CK antibody (ab7753; Abcam, Shanghai, China), α-SMA antibody (ab124964; Abcam), PDPN antibody (ab10288; Abcam) and ITGA11 antibody (AP10393a; Abcepta, Guangzhou, China) separately. Both antibodies were diluted at a ratio of 1:200 in 5% bovine serum albumin (BSA) and the incubation was carried out at room temperature for 1 hour. Subsequently, the sections were blocked with 5% BSA. The following secondary antibodies were used: Alexa Fluor anti-rabbit A488 (1:200, 4412S; Cell Signaling Technology, Boston, MA, USA) or anti-mouse A488 (1:200, ab150113; Abcam) for α-SMA or PDPN staining and anti-mouse A555 (1:400, 4409S; Cell Signaling Technology) or anti-rabbit A555 (1:400, 4413S; Cell Signaling Technology) for PAN-CK or ITGA11 staining. The PAN-CK or ITGA11 staining exhibited a pseudored color, while the α-SMA or PDPN staining displayed a pseudogreen color. The cell nuclei were counterstained with 4',6-diamidino-2-phenylindole (DAPI). Immunofluorescence images were captured using an OLYMPUS IX83-FV3000 laser scanning confocal microscope.

1. Fluorescence in situ hybridization

The Fluorescence in situ hybridization (FISH) kit was purchased from RiboBio (Guangzhou, China). All experiments were conducted in accordance with the manufacturer's instructions. Briefly, for cell preparation, the cells were fixed in 4% paraformaldehyde and permeabilized with 0.1% Triton X-100. In the case of tissue preparation, deparaffinized and rehydrated tissue sections were subjected to digestion with proteinase K. Following a 30-minute pre-hybridization step, the cell or tissue samples were incubated with specific probes (from the FISH kit; RiboBio) at 37°C. The Cy3-labeled probe for circTAX1BP1, with the sequence 5′-TACTTCAGTAAGACCTTTATAATTGTCCTGT-3′, was designed and synthesized by GenePharma (Shanghai, China). Image acquisition was performed using an Olympus IX83-FV3000 laser scanning confocal microscope.

1. Western blot

Total proteins were extracted using RIPA lysis buffer (CwBiotech, Jiangsu, China). The protein concentration was determined using a BCA protein assay kit (CwBiotech). The protein extracts were separated by 6% or 10% sodium dodecyl sulfate-polyacrylamide gel electrophoresis (SDS-PAGE) and then transferred onto a nitrocellulose membrane (Millipore, Billerica, MA, USA). After blocking for 1 hour, the membrane was incubated with primary antibodies overnight at 4°C. The next day, the membrane was incubated with secondary antibodies conjugated to horseradish peroxidase (HRP) (1:2000, 7074S; Cell Signaling Technology) at room temperature for 1 hour. Immunoreactive bands were detected using an ECL kit (Thermo, Waltham, MA, USA). The following primary antibodies were used: anti-α-SMA antibody (1:1000, ab124964; Abcam), anti-FAP antibody (1:1000, ab314456; Abcam), anti-PDGFR-α antibody (1:1000, ab203491; Abcam), anti-CD63 antibody (1:1000, ab134045; Abcam), anti-ALIX antibody (1:1000, ab275377; Abcam), anti-TSG101 antibody (1:1000, ab125011; Abcam), anti-GM130 antibody (1:1000, ab52649; Abcam), anti-VIRMA antibody (1:1000, ab271136; Abcam), anti-Histone H3 antibody (1:1000, ab1791; Abcam), anti-L-Lactyl Lysine antibody (1:1000, 1401RM; PTM BIO, Hangzhou, China), anti-AARS2 antibody (1:1000, 22696-1-AP; Proteintech, Wuhan, China), anti-SP1 antibody (1:1000, 9389S; Cell Signaling Technology), anti-TGF-β1 antibody (1:1000, ab215715; Abcam), anti-Smad2 antibody (1:2000, ab40855; Abcam), anti-p-Smad2 antibody (1:1000, ab280888; Abcam), anti-Smad3 antibody (1:1000, ab84177; Abcam), anti-p-Smad3 antibody (1:2000, ab52903; Abcam), anti-N-cadherin antibody (1:1000, 13116S; Cell Signaling Technology), anti-E-cadherin antibody (1:1000, 3195S; Cell Signaling Technology), anti-Vimentin antibody (1:1000, 5741S; Cell Signaling Technology), anti-Snail antibody (1:1000, 3879S; Cell Signaling Technology), anti-Slug antibody (1:1000, 9585S; Cell Signaling Technology), and anti-ITGA11 antibody (1:1000, ab198826; Abcam). The band densities were normalized to either β-actin (1:4000, 20536-1-AP; Proteintech) or GAPDH (1:5000, 10494-1-AP; Proteintech). Quantification of the bands was performed using a Biolight BLT GelView 6000 Pro machine.

1. Exosome isolation

Briefly, CAFs or NFs were cultured in DMEM/F12 medium supplemented with 10% exosome-depleted fetal bovine serum (FBS) for 48 hours. The culture supernatants were then collected. To isolate exosomes, the supernatants were initially centrifuged at 2000 ×g for 10 minutes and subsequently at 10,000 ×g for 30 minutes to eliminate cellular debris and apoptotic bodies (Beckman Avanti Centrifuge J-26XP, Beckman Coulter, Brea, CA, USA, https://www.beckmancoulter.com). Following this, the supernatants were harvested and subjected to ultracentrifugation at 110,000 ×g for 70 minutes using a Beckman Optima L-80 XP ultracentrifuge equipped with a 70Ti rotor. The resulting exosome pellets were then washed with sterilized PBS and further purified by centrifugation at 110,000 ×g for 1 hour. All centrifugation procedures were carried out at 4°C. Subsequently, the exosomes were resuspended in PBS and filtered through a 0.22 μm filter (Millipore, Darmstadt, Germany, http://www.emdmillipore.com). The total protein concentration in each exosome preparation was quantified using the Bradford assay (Pierce, Rockford, IL, USA). The exosomes were then stored at -80°C until further use.

1. Exosome characterization

The morphological features of the isolated exosomes were characterized using a Hitachi HT7700 transmission electron microscope (TEM; Hitachi, Tokyo, Japan). Briefly, the isolated exosomes were fixed with 2% paraformaldehyde and then applied onto glow-discharged copper grids supported by filter paper. The copper grids were subsequently allowed to dry at room temperature for 15 minutes. Following this, the samples were stained with 2% uranyl acetate and left to dry for an additional 10 minutes. Subsequently, the samples were examined under the TEM at an accelerating voltage of 100 keV. The size distribution of the isolated exosomes was analyzed using a qNano device (Horizon Science, New Zealand) in accordance with the manufacturer's instructions. Western blot analysis was performed to detect exosomal protein markers using anti-CD63, anti-ALIX, anti-TSG101, and anti-GM130 antibodies.

1. Labeling of exosomes

In accordance with the manufacturer's instructions, CAFs were pre-labeled with CM-DiI (Invitrogen). Subsequently, the labeled CAFs were washed three times with PBS to remove any excess dye. In certain experimental conditions, the CAFs were pre-treated with 10 μM GW4869 (Sigma) for 12 hours prior to further procedures. The CAFs were then cultured on confocal dishes and co-incubated with colorectal cancer cells for a duration of 4 hours. Following the co-incubation, the cells were washed three times with PBS. The cells were then fixed with 4% paraformaldehyde (PFA) for 10 minutes and permeabilized with ice-cold methanol for 15 minutes. To visualize the cell nuclei, DAPI staining was performed. Finally, the cells were imaged using a confocal microscope (OLYMPUS IX83-FV3000, Tokyo, Japan).

1. Plasmids, small interfering (si)RNA, and cell transfection

The full-length cDNAs of circTAX1BP1 and AARS2 were amplified and subsequently cloned into the pcDNA3.1 vector, which was obtained from Generay (Shanghai, China). To transiently silence the expression of circTAX1BP1 or AARS2 in CAFs or colorectal cancer cells, we utilized small interfering RNAs (siRNAs) specifically designed and synthesized by GenePharma (targeting human circTAX1BP1 or AARS2, as detailed in Supplementary Table 6). The vectors, along with the siRNAs where applicable, were transfected into the cells using Lipofectamine 3000 transfection reagent (Invitrogen), following the manufacturer's instructions. 48 hours post-transfection, the transfection efficiency in each group of cells was evaluated using qRT-PCR.

1. CCK-8 and colony formation detection

To assess cell proliferation, transfected cells were seeded into 96-well plates at a density of 1×10³ cells per well and cultured for varying durations (1, 2, 3, 4, or 5 days). Subsequently, 20 μL of Cell Counting Kit-8 (CCK-8) solution (APExBIO, Houston, Texas, USA) was added to each well, followed by incubation at 37°C for 4 hours. The optical density values at 450 nm were measured using a microplate reader (ELx800; BioTek Instruments, Winooski, VT, USA).

For the colony formation assay, 3×10³ cells were seeded into 6-well plates and incubated in complete culture medium at 37°C. After 2 weeks of incubation, the colonies were fixed with 4% paraformaldehyde and stained with 0.5% crystal violet. Colonies containing at least 50 cells were scored and observed under an IX71 inverted phase-contrast fluorescence microscope (Olympus, Tokyo, Japan). The data are presented as the mean ± standard deviation of five randomly selected fields.

1. Migration and invasion assay

Cell migration was assessed using Transwell chambers (8 μm, 24-well inserts; Corning, Lowell, MA, USA). Briefly, the lower chambers were filled with 600 μL of culture medium containing 10% FBS, while the upper chambers were loaded with 200 μL of serum-free medium containing 1×10⁴ cells. The chambers were then incubated at 37°C for 48 hours. Non-migrated cells on the upper surface of the membrane were carefully removed using a cotton swab. Subsequently, the migrated cells on the lower surface of the membrane were fixed with 4% paraformaldehyde and transferred to a membrane stained with 0.5% crystal violet. The number of migrated cells was counted under an IX71 microscope (Olympus, Tokyo, Japan). For the cell invasion assay, the inserts were pre-coated with diluted Matrigel (BD Biosciences, San Diego, CA, USA). The remaining steps of the invasion assay were identical to those described for the migration assay.

1. Wound healing assay

Cells were seeded at a density of 4 × 10^4^ cells/cm² and allowed to grow until they reached approximately 90% confluence. To initiate the wound healing assay, a scratch was made across the cell monolayer using a sterile 10 μl pipette tip. The cell debris and detached cells were removed by washing the cultures three times with PBS. Subsequently, fresh serum-free medium was added to the cultures. Images of the wounded areas were captured at various time points (0, 12, 24, and 48 hours) under a microscope to monitor cell migration and wound closure.

1. Animal studies

All experimental procedures were approved by the Institutional Animal Care and Use Committee (IACUC) of Sun Yat-sen University (Approval No: SYSU-IACUC-2025-000719). Four-week-old male BALB/c nude mice were purchased from GemPharmatech LLC (Nanjing, China) and randomly divided into four groups (n=6 per group): the PBS group, the CAF-EVs group, the CAF-EVs siRNA con group, and the CAF-EVs circTAX1BP1 siRNA group. For the subcutaneous tumor model, 5 × 10⁶ HCT116 cells were suspended in 100 μL of PBS and injected subcutaneously into the dorsal flank of each nude mouse. Subsequently, the four groups were subjected to different treatments, including the injection of 10 μg of EVs or PBS at the tumor site every three days. Tumor volume (TV) was measured weekly using the formula: TV (mm³) = length × (width)² × 0.5. The mice were euthanized four weeks after inoculation, and the tumor tissues were excised and stored at -80°C for further immunohistochemical (IHC) analysis. For the metastasis model, 5 × 10⁶ HCT116 cells in 100 μL of PBS were injected into the tail vein of the mice to observe metastatic spread. The mice were euthanized 1.5 months later, and the livers were collected and photographed. The number of liver metastatic tumor nodules was counted for each mouse.

1. Immunohistochemistry (IHC)

For IHC analysis, primary antibodies against SP1 (1:1000, 9389S; Cell Signaling Technology), TGF-β1 (1:500, ab215715; Abcam), p-Smad2 (1:1000, ab280888; Abcam), p-Smad3 (1:100, ab52903; Abcam) and Ki-67 (1:200, 9027S; Cell Signaling Technology) were utilized. Tissue specimens were first dewaxed and rehydrated. Antigen retrieval was performed by treating the sections with boiling citrate buffer for 6 minutes. Subsequently, the slides were blocked with a blocking reagent (Millipore) for 10 minutes. The primary antibodies were then applied, and the sections were incubated overnight at 4°C. After washing with Tris-buffered saline containing 0.1% Tween-20 (TBST), the sections were incubated with secondary antibodies (Millipore) for 45 minutes. Staining was carried out using an HRP/DAB kit (Millipore). IHC images were captured under a microscope at 10× and 20× magnifications. IHC staining was scored by the percentage of positive area and intensity as follows: 0, no staining; 1, <10% positive, moderate, or strong intensity; 2, 10–50% positive, moderate, or strong intensity; 3, >50% positive, moderate intensity; and 4, >50% positive, strong intensity.

1. H&E staining

Freshly procured mouse subcutaneous tumors, mouse liver tissues, and human colorectal cancer tissue specimens were immediately fixed in 4% paraformaldehyde (PFA) solution with a pH of 7.4. Following fixation, the tissues were subjected to a systematic and gradual dehydration process. Once dehydrated, the tissues were carefully embedded in paraffin wax to create paraffin-embedded blocks. From these blocks, thin sections with a uniform thickness of 3 μm were meticulously cut. These sections were then stained using the hematoxylin and eosin (H&E) staining protocol. After staining, the H&E-stained sections were placed under a light microscope for comprehensive histological examination, enabling the visualization and analysis of tissue morphology and cellular architecture.

1. RNA Immunoprecipitation (RIP) assay

The RNA immunoprecipitation (RIP) experiment was conducted in strict accordance with the instructions provided by the manufacturer of the Magna RIP RNA-Binding Protein Immunoprecipitation Kit (Millipore). Magnetic beads were coated with 10 μg of antibodies, namely anti-IgG (ab172730; Abcam), anti-AGO2 (67934-1-IG; Proteintech), or anti-VIRMA (25712-1-AP; Proteintech) antibodies, by incubating them at room temperature for 30 minutes. This step ensured the surface of the magnetic beads was adequately functionalized with the respective antibodies. Cell lysates were prepared from 2 × 10⁷ cells. These lysates were then incubated overnight with the antibody-coated magnetic beads. This prolonged incubation allowed for efficient binding of RNA-protein complexes to the antibodies on the magnetic beads. Subsequently, the magnetic bead-protein-RNA complexes were washed thoroughly with wash buffer to remove any unbound or non-specifically bound components. Following the washing step, the complexes were incubated in a proteinase K digestion buffer at 55°C for 30 minutes. This digestion step facilitated the release of RNA from the protein-RNA complexes. The purified RNAs obtained after the proteinase K treatment were subjected to qRT-PCR analysis.

1. RNA Pull-down assay

RNA pull-down experiments were carried out using the Pierce™ Magnetic RNA-Protein Pull-Down Kit (Millipore). Biotin-labeled control oligomers (UGCUUUGCACGGUAACGCCUGUUUU-bio, referred to as the control probe) or oligomers complementary to the sequence of circTAX1BP1 (TACTTCAGTAAGACCTTTATAATTGTCCTGT-bio, also known as the circTAX1BP1 probe) were synthesized by GenePharma (Shanghai, China). The oligomers were incubated with lysates obtained from colorectal cancer cells at 25°C for 2 hours. This incubation step allowed for the formation of RNA/protein complexes. Subsequently, streptavidin-coupled Dynabeads were added to the reaction mixtures to capture the RNA/protein complexes at 25°C for 1 hour. The pulled-down complexes were then analyzed by Western blotting and qRT-PCR. Finally, the samples were subjected to SDS-PAGE followed by mass spectrometry (MS) analysis.

1. m6A Dot blot assay

The concentration of mRNA extracted from total cellular RNA was determined using a NanoDrop 2000 spectrophotometer. The mRNA samples were then diluted to various concentrations with RNase-free water. For dot blotting, the serially diluted mRNA samples were subjected to denaturation at 95°C for 3 minutes to disrupt secondary structures, followed by rapid chilling on ice to maintain the denatured state. The nucleic acids were transferred onto a Hybond-N+ membrane (Beyotime, Shanghai, China) using a dot blotter apparatus. After transfer, the membrane was exposed to 2400 mJ/cm² of ultraviolet (UV) light to cross-link the nucleic acids to the membrane, ensuring their stable attachment. Following washing steps to remove unbound materials, the membrane was incubated with an m6A antibody (1:5000, ab284130; Abcam) in a 5% BSA solution at 4°C overnight. This incubation allowed for the specific binding of the m6A antibody to m6A-modified mRNA present on the membrane. On the following day, the membrane was incubated with HRP-conjugated secondary antibody (1:2000, 7074S; Cell Signaling Technology) for an additional 1 hour at room temperature. The bound antibodies were then visualized using ECL reagents (Thermo Fisher Scientific, Waltham, MA, USA). As a loading control to ensure equal amounts of RNA were applied across the membrane, the membrane was stained with methylene blue in a 0.3 M sodium acetate solution. This staining provided a visual indication of the total RNA loaded in each dot, allowing for normalization of the m6A signal.

1. Cytoplasmic and nuclear protein extraction

Nuclear and cytoplasmic proteins were extracted using a commercial kit (Beyotime) in accordance with the manufacturer's instructions. Briefly, cells were harvested and suspended in Solution A containing phenylmethylsulfonyl fluoride (PMSF). The cell suspension was then incubated on ice for 10 minutes to allow for initial cell lysis. Subsequently, 10 μL of Solution B was added to the cell lysate, and the mixture was kept on ice for an additional 1 minute to facilitate further disruption of the cell membrane and release of cytoplasmic contents. Following this, the lysate was centrifuged at 15,000 rpm. The supernatant, which contained the cytoplasmic proteins, was carefully collected, and set aside for further analysis. To extract nuclear proteins, the remaining pellet was resuspended in 50 μL of nuclear protein extraction reagent supplemented with PMSF. The resuspended pellet was vortexed briefly to ensure thorough mixing. Then, the sample was centrifuged at 12,000 rpm. The resulting supernatant, now enriched in nuclear proteins, was collected.

1. Co-immunoprecipitation

For immunoprecipitation, whole-cell extracts were lysed in NP-40 lysis buffer (Beyotime) supplemented with a protease inhibitor cocktail (Millipore) and PMSF (Beyotime). When necessary, to disrupt protein-protein interactions, the cell lysates were treated with 1% sodium dodecyl sulfate (SDS) and incubated at 90°C for 5 minutes. The cell lysates were then centrifuged at 12,000g for 15 minutes. The resulting supernatants were collected and incubated with protein A/G magnetic beads (Invitrogen) conjugated to specific antibodies or anti-FLAG M2 magnetic beads (Sigma). After overnight incubation of the magnetic beads with the lysates, the beads were washed five times using IP washing buffer. Subsequently, the bound proteins were eluted with SDS-PAGE sample buffer for further analysis.

1. Methylated RNA immunoprecipitation (meRIP)-qPCR

RNA was extracted from CRC cells using Trizol Reagent (Invitrogen). Subsequently, mRNA was purified using the Dynabeads mRNA Purification Kit (Invitrogen). A portion of the total RNA was reserved as an input control. The samples were incubated overnight at 4°C in an immunoprecipitation (IP) buffer containing an m6A antibody (Synaptic Systems, Göttingen, Germany). Following this incubation, the samples containing m6A-modified RNA underwent immunoprecipitation and subsequent elution steps. To quantify the enrichment of m6A in each sample, the levels of m6A-modified RNA were determined by qRT-PCR, with normalization against the input RNA.

1. Dual-Luciferase reporter assays

To assess luciferase activity, a Dual-Luciferase Reporter Assay Kit (Promega, Madison, WI, USA) was employed. The reporter plasmids were constructed using the GeneChem pGL3 expression vector, which contained both firefly and Renilla luciferase genes. In the mutant SP1 reporter plasmid, an adenine (A) base was substituted with a guanine (G) base. Detailed descriptions of other mutant reporter plasmids are provided in the Results section. Luciferase activity was measured using the Duo-Lite Luciferase Assay System (Vazyme) in accordance with the manufacturer's instructions.

1. Chromatin immunoprecipitation (ChIP)

The ChIP experiments were conducted using the EZMagna ChIP A/G Kit (Millipore). A total of 1 × 10⁸ cells were fixed in 1% formaldehyde at room temperature for 10 minutes. Subsequently, the nuclei were isolated using a nuclear lysis buffer supplemented with protease inhibitors. The chromatin DNA was sonicated and sheared into fragments ranging from 100 to 200 bp. The sheared chromatin was then subjected to overnight immunoprecipitation at 4°C using either an anti-SP1 antibody (9389S; Cell Signaling Technology) or an anti-SMAD3 antibody (9523S; Cell Signaling Technology). For negative controls (NCs), normal rabbit IgG (ab172730; Abcam) or an antibody against β-actin (66009-1-Ig; Proteintech) was employed. An anti-RNA polymerase II (RNA pol II) antibody (Millipore) served as the positive control. The primers used for ChIP-qPCR are listed in Supplementary Table 7.

1. ELISA analysis of supernatants from CRC cells after exosome treatment

The TGF-β concentration in cell culture medium was measured by ELISA using the Quantikine human ELISA kit (Fine Biotech, Wuhan, China) according to the manufacturer’s instructions.

1. Establishment of mice carrying patient-derived xenografts (PDXs)

In the PDX mouse model, 4-week-old non-obese diabetic/severe combined immunodeficient/inter-leukin-2 receptor gamma-chain null (NSG) mice were used. Fresh colorectal cancer tissues were obtained from two surgical patients at Sun Yat-sen Memorial Hospital, Sun Yat-sen University, and were subcutaneously implanted into these mice. Once the tumors reached a volume of approximately 200 mm³, the mice were randomly divided into four groups. Subsequently, the mice were administered treatments with si-circTAX1BP1 and/or a TGF-β neutralizing antibody (fresolimumab). This experimental protocol was approved by the Institutional Animal Care and Use Committee (IACUC) of Sun Yat-sen University (Approval No: SYSU-IACUC-2025-000718).

1. Data sources

We downloaded the high throughput RNA sequencing data of 461 colorectal cancer tissues from TCGA ^[1]^ website (TCGA-COAD, https://portal.gdc.cancer.gov/). The transcripts per million (TPM) value was used. Clinical information for the TCGA-COAD cohort was downloaded for analysis using the TCGAbiolinks package. Microarray data sets were obtained from the GEO database (Geo accession numbers: GSE131418), https://www.ncbi.nlm.nih.gov/geo/). Clinical information for the GEO datasets was downloaded from the GEO database or from the Supplementary Materials of the original literature.

1. Bulk RNAseq analysis of colorectal cancer

For TCGA data, genes were filtered if expressed in less than twenty percent of samples. Read counts were TMM normalized using the edgeR package ^[2]^. Normalized RNAseq abundances were log2 transformed. Differential gene expression analysis was performed using the voom algorithm (default settings, limma package ^[3]^) due to the highly variable library sizes. LmFit algorithm was used to fit the data using a linear model (trend = TRUE, limma package). Differential expression between different groups was calculated using the eBayes algorithm (default settings, limma package). Heatmaps of gene expression were generated using the ComplexHeatmap package ^[4]^. For the GEO datasets, the microarray datasets were integrated for further analysis and a batch normalization algorithm (sva package ^[5]^) was employed to remove batch effects.

1. Pathway enrichment analyses

Enrichment pathway analysis was performed on the 50 hallmark pathways and the canonical pathways derived from the Reactome pathway database using the Msigdb database(https://www.gsea-msigdb.org/gsea/msigdb). The human gene information was obtained from ‘org.Hs.eg.db’ package. The gene symbol used for enrichment analysis was transformed into Entrez ID using the bitr algorithm (OrgDb = org.Hs.eg.db, ClusterProfiler package ^[6]^).

The enricher algorithm (default setting, ClusterProfiler package) was used for enrichment. If *p* < 0.05, the pathway was considered to be significantly enriched. Gene Set Variation Analysis was applied to each sample using gsva algorithm (default settings, GSVA package ^[7]^) to calculate the enrichment score of a particular gene set. Differential pathway enrichment analysis between different groups was assessed using the ‘Limma’ package. The ‘ggplot2’ package ^[8]^, ‘ggpubr’ package was used for visualization.

1. Single-cell transcriptome data analysis of colorectal cancer

Single-cell transcriptome data GSE161277, GSE166555, GSE164522, GSE225857, GSE200997, GSE178318, and GSE132257 was obtained from the GEO database. Using the Seurat package version 4.0.4 ^[9]^, quality control was performed on each sample using standard single-cell processing procedures. We filtered low quality cells using the following filter criteria: if a gene was expressed in less than 3 cells, the gene was deleted. Genes that were shared by three or more cells were retained. Cells with fewer than 500 features and 6,000 or more features and with mitochondrial RNA percentages of >20 and a novelty score (log10GenesPerUMI) smaller than 0.8 were filtered out. Individual Seurat objects were normalized using the “SCTransform” algorithm along with regression of mitochondrial RNA as a variable. The “SelectIntegrationFeatures” algorithm was run to select features as the highly variable features. The “doubletFinder_v3” algorithm (DoubletFinder package ^[10]^) was used to identify doublets from each dataset with the “pN” value was set to 0.25 and the “nExP” was set to the expected doublet rate according to the Chromium Single-Cell 3′ v2 reagents kit user guide (10X Genomics). Doublet cells were removed from the data set after being identified. Then the datasets were merged into an object using the “merge” algorithm. Through cell filtering and mering, we obtained a total of 181950 cells. The “RunPCA” algorithm was used to perform principal component analysis for the merge dataset, and the first 30 principal components were identified for analysis. The “RunHarmony” algorithm (Harmony package ^[11]^) was used to remove batch effects between samples. The “RunTSNE” algorithm (reduction = ‘harmony’, dims = 1:15) was used for dimensionality reduction. Clustering analysis was performed by the “FindNeighbors” and “FindClusters” algorithm (resolution=1). CopyKAT is used to identify tumor cells. For the subclustering of CAF populations, we first subsetted CAF cells from the integrated single-cell dataset, performed FindNeighbors and FindClusters (resolution = 1), followed by dimensionality reduction using the RunTSNE algorithm (reduction = 'harmony', dims = 1:15). We used the “RNA” assay data for all further downstream analyses such as gene-level visualization, or differential expression analysis. Differential geneexpression analysis was conducted using the “FindAllMarkers” or “FindMarkers” algorithm and genes expressed in at least 25% of the cells were selected. The “DoHeatmap,” “FeaturePlot,” “DimPlot,” “DotPlot,” and “VlnPlot” algorithm were used for visualization. All cell lineages were identified by marker gene expression according to relevant literature and the CellMarker database (http://xteam.xbio.top/CellMarker/). The “Addmodulescore” algorithm was used to calculate the average expression levels of each gene set for each cell type at the single-cell level.

1. Survival analysis

The Kaplan–Meier plotter was used to plot the prognostic survival curve for different groups (“survfit” algorithm, survminer package ^[12]^), and the log-rank test was used to assess statistically significant survival differences between groups.

1. Flow cytometry and sorting of primary CAF subsets

The infiltration of ITGA11⁺ myCAFs in fresh CRC tissues obtained from patients who had undergone surgical resection at Sun Yat-sen Memorial Hospital was assessed using flow cytometry. Briefly, the tissues were processed using a tumor dissociation kit (Miltenyi Biotec, Germany) to generate single-cell suspensions. These suspensions were then stained with the live/dead "fixable viability dye" eFluor780 (eBioscience, San Diego, CA, USA) and the specified antibodies for 20 minutes at room temperature. Following this, the stained suspensions were washed three times with PBS. Subsequently, the treated single-cell suspensions were analyzed using a FACScan flow cytometer (Becton Dickinson, Brea, CA, USA). The data obtained were processed using FlowJo 10.7 software (TreeStar, Ashland, OR, USA). To exclude specific cell types, antibody signals against the following marker proteins were utilized: CD45 (304006; Biolegend, San Diego, CA, USA) for hematopoietic cells, CD31 (557508; BDBiosciences, Franklin Lakes, NJ, USA) for endothelial cells, and EPCAM (324204; Biolegend) for epithelial cells. The proportion of total cells exhibiting antibody signals for both ITGA11 and PDPN was calculated to determine the abundance of ITGA11^+^ myCAFs within the CRC tissues.

For the sorting of primary CAF subsets, the preparation and staining of single-cell suspensions were carried out as described above. The ITGA11^+^ myCAFs, ITGA11^+^-d myCAFs (myCAFs depleted of this subpopulation), and inflammatory CAFs (iCAFs) were isolated from the single-cell suspensions using a BD Influx flow cytometer. The following antibodies were employed for the isolation: anti-ITGA11 (AP10393a; Abcepta) conjugated with BV421, anti-IL-1R1 (FAB269P; R&D Systems, Minneapolis, MN, USA) conjugated with PE, and anti-PDPN (ab236529; Abcam) conjugated with APC. The isolated CAF subsets were further verified by immunofluorescence and flow cytometry. The isolated CAF subsets were cultured in Fibroblast medium (ScienCell Research Laboratories, Carlsbad, CA, USA) supplemented with 2.5% fetal bovine serum (FBS) and 1% growth factor. The cultures were maintained in a humidified incubator with 5% CO₂ at 37°C.

**References**

[1] Comprehensive genomic characterization of squamous cell lung cancers [J]. Nature, 2012, 489(7417): 519-25. doi:10.1038/nature11404

[2] Robinson M D, McCarthy D J, Smyth G K. edgeR: a Bioconductor package for differential expression analysis of digital gene expression data [J]. Bioinformatics (Oxford, England), 2010, 26(1): 139-40. doi:10.1093/bioinformatics/btp616

[3] Ritchie M E, Phipson B, Wu D, et al. limma powers differential expression analyses for RNA-sequencing and microarray studies [J]. Nucleic acids research, 2015, 43(7): e47. doi:10.1093/nar/gkv007

[4] Gu Z, Eils R, Schlesner M. Complex heatmaps reveal patterns and correlations in multidimensional genomic data [J]. Bioinformatics (Oxford, England), 2016, 32(18): 2847-9. doi:10.1093/bioinformatics/btw313

[5] Leek J T, Johnson W E, Parker H S, et al. The sva package for removing batch effects and other unwanted variation in high-throughput experiments [J]. Bioinformatics (Oxford, England), 2012, 28(6): 882-3. doi:10.1093/bioinformatics/bts034

[6] Wu T, Hu E, Xu S, et al. clusterProfiler 4.0: A universal enrichment tool for interpreting omics data [J]. Innovation (Cambridge (Mass)), 2021, 2(3): 100141. doi: 10.1016/j.xinn.2021.100141

[7] Hänzelmann S, Castelo R, Guinney J. GSVA: gene set variation analysis for microarray and RNA-seq data [J]. BMC bioinformatics, 2013, 14: 7. doi:10.1186/1471-2105-14-7

[8] Wickham H. ggplot2 - Elegant Graphics for Data Analysis; proceedings of the Use R!, F, 2009 [C].

[9] Hao Y, Hao S, Andersen-Nissen E, et al. Integrated analysis of multimodal single-cell data [J]. Cell, 2021, 184(13): 3573-87. e29. DOI: 10.1016/j.cell.2021.04.048

[10] McGinnis C S, Murrow L M, Gartner Z J. DoubletFinder: Doublet Detection in Single-Cell RNA Sequencing Data Using Artificial Nearest Neighbors [J]. Cell systems, 2019, 8(4): 329-37. e4. DOI: 10.1016/j.cels.2019.03.003

[11] Korsunsky I, Millard N, Fan J, et al. Fast, sensitive and accurate integration of single-cell data with Harmony [J]. Nature methods, 2019, 16(12): 1289-96. doi:10.1038/s41592-019-0619-0

[12] Kassambara A, Kosinski M, Biecek P. Drawing Survival Curves using 'ggplot2' [R package survminer version 0.4.8], F, 2020 [C].
