# Supplementary material for: Extracellular Vesicle‐Packaged circTAX1BP1 from Cancer‐Associated Fibroblasts Regulates RNA m6A Modification through Lactylation of VIRMA in Colorectal Cancer Cells
Source: Adv Sci (Weinh). 2025 Sep 29;12(47):e14008. doi: 10.1002/advs.202514008 (PMC12713077; doi:10.1002/advs.202514008)
Supplement: Supplementary file 2 — Supporting Information [file ADVS-12-e14008-s003.docx]

**
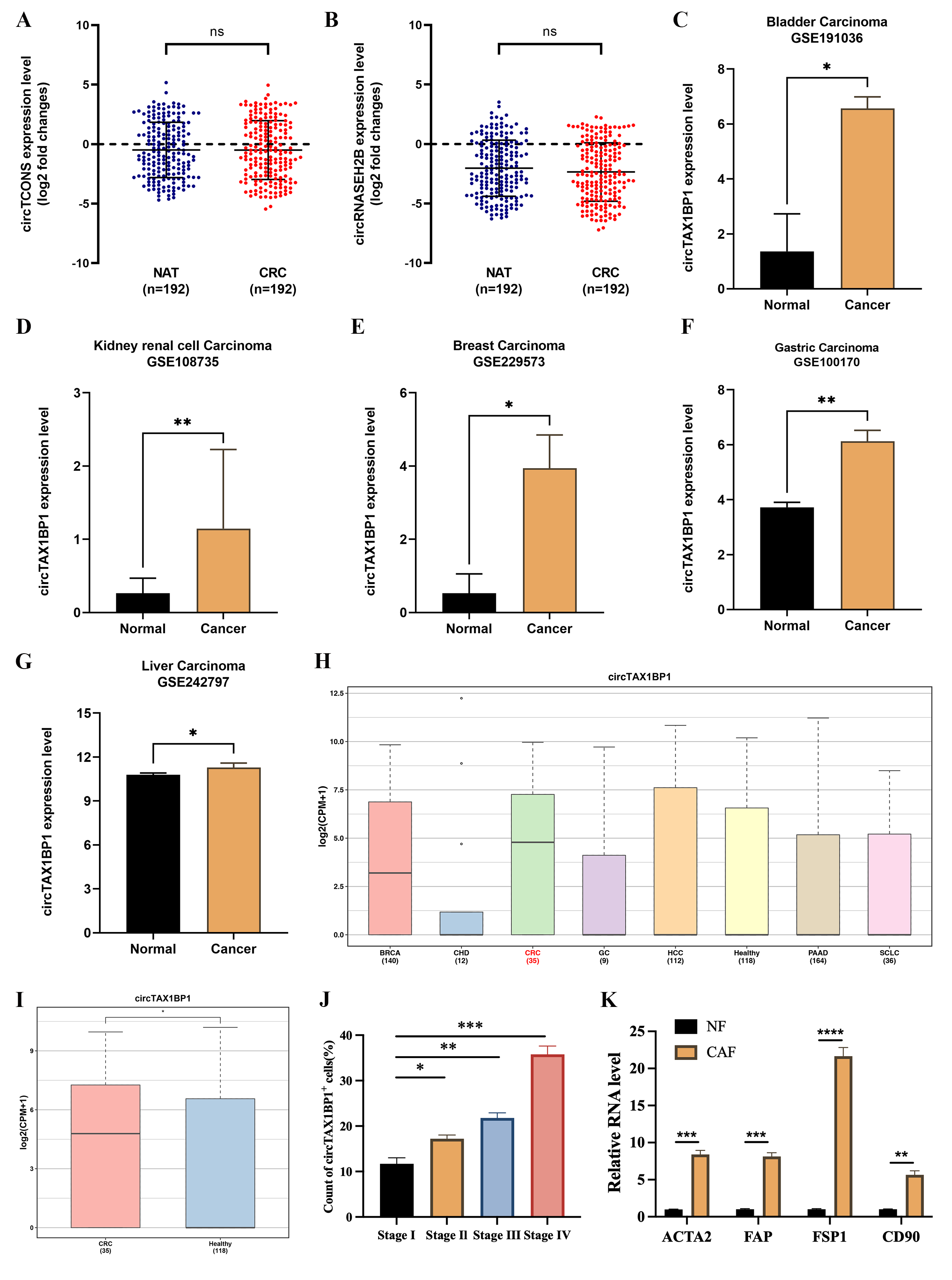
**

**Supplementary Figure 1.** **Expression analysis of** **circTAX1BP1 and related markers in colorectal cancer (CRC) cells and associated cell types.** A–B. qRT-PCR analysis of circTCONS and circRNASEH2B expression in a 192-case cohort of freshly collected human CRC samples and NATs. C–G. Data from the GEO database for different types of cancers were analysed. H. Boxplot displaying the expression abundance of circTAX1BP1 in blood exosomes from the exoRBase database for patients with different cancers and healthy individuals. I. Boxplot depicting the expression levels of circTAX1BP1 in blood exosomes from the exoRBase database between patients with CRC and healthy controls. J. Detection of circTAX1BP1-positive cells in stromal tissues of patients with CRC at different disease stages (n = 3). K. qRT-PCR analysis of mRNA expression levels of ACTA2, FAP, FSP1, and CD90 in isolated CAFs and NFs (n = 3). The statistical difference was assessed through nonparametric Mann–Whitney U test in (A-G, I, and K); and one-way ANOVA followed by Dunnett tests in (J). All data are presented as mean ± SD of experimental triplicates. ns, *P* > 0.05; *, *P* < 0.05; **, *P* < 0.01; ***, *P* < 0.001; ****, *P* < 0.0001.


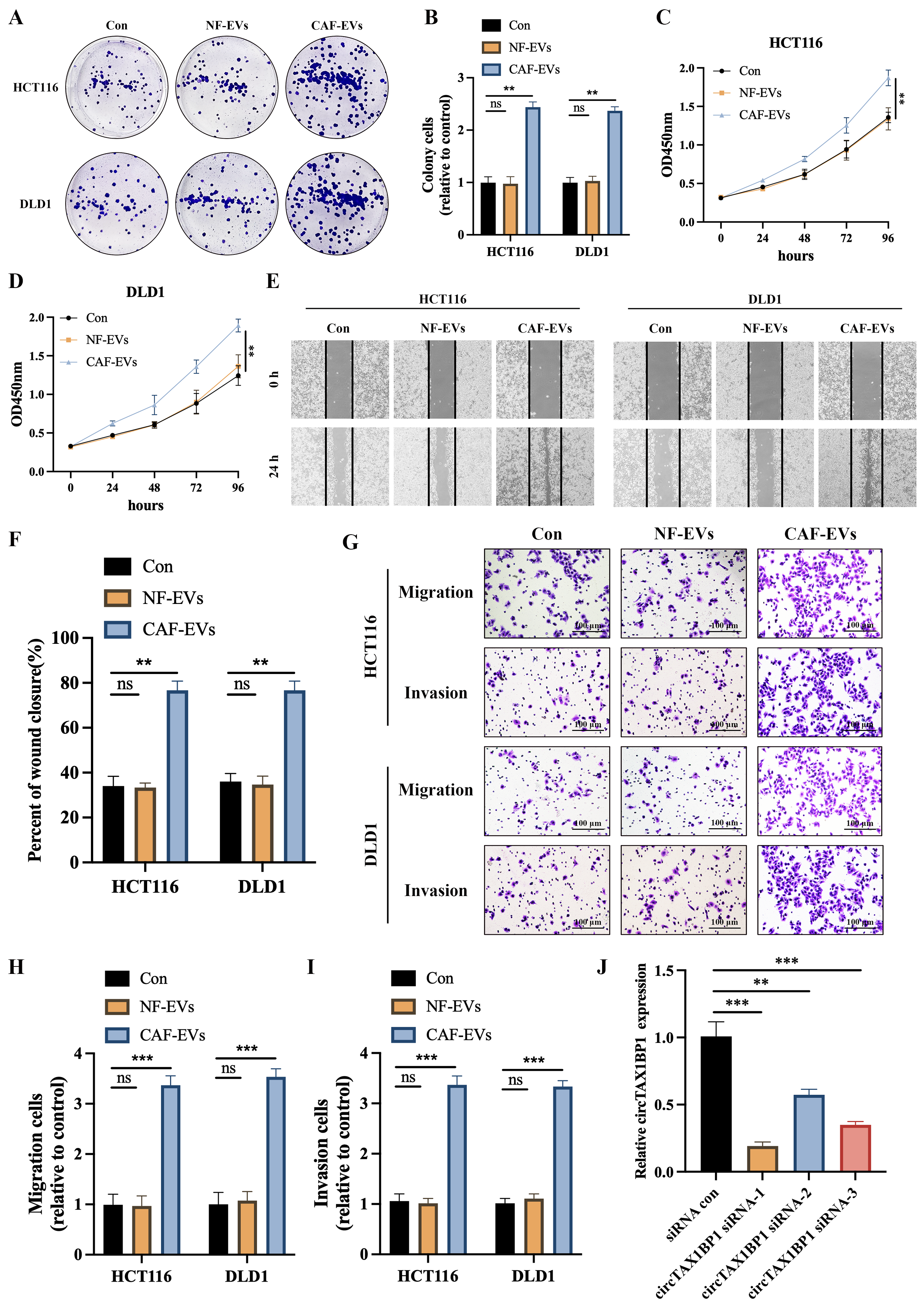


**Supplementary Figure 2. Assessment of cellular functions and effects of circTX1BP1 in colorectal cancer (CRC) cells.** A–B. Colony formation assays were performed for HCT116 and DLD1 cells (n = 3). C–D. Proliferation of HCT116 and DLD1 cells was measured via CCK-8 assays (n = 3). E–F. Representative images of wound healing assay using HCT116 and DLD1 cells showing cell motility, and a histogram analysis of cell migration distance was performed (n = 3). G–I. Representative images of Transwell assays, and migration and invasion of HCT116 and DLD1 cells were assessed (n = 3). Scale bar, 100 µm. J. CAFs were exposed to circTX1BP1 siRNA for 24 h (n = 3). The statistical difference was assessed through one-way ANOVA followed by Dunnett tests in (B-D, F, and H-J). All data are presented as mean ± SD of experimental triplicates. ns, *P* > 0.05; **, *P* < 0.01; ***, *P* < 0.001.


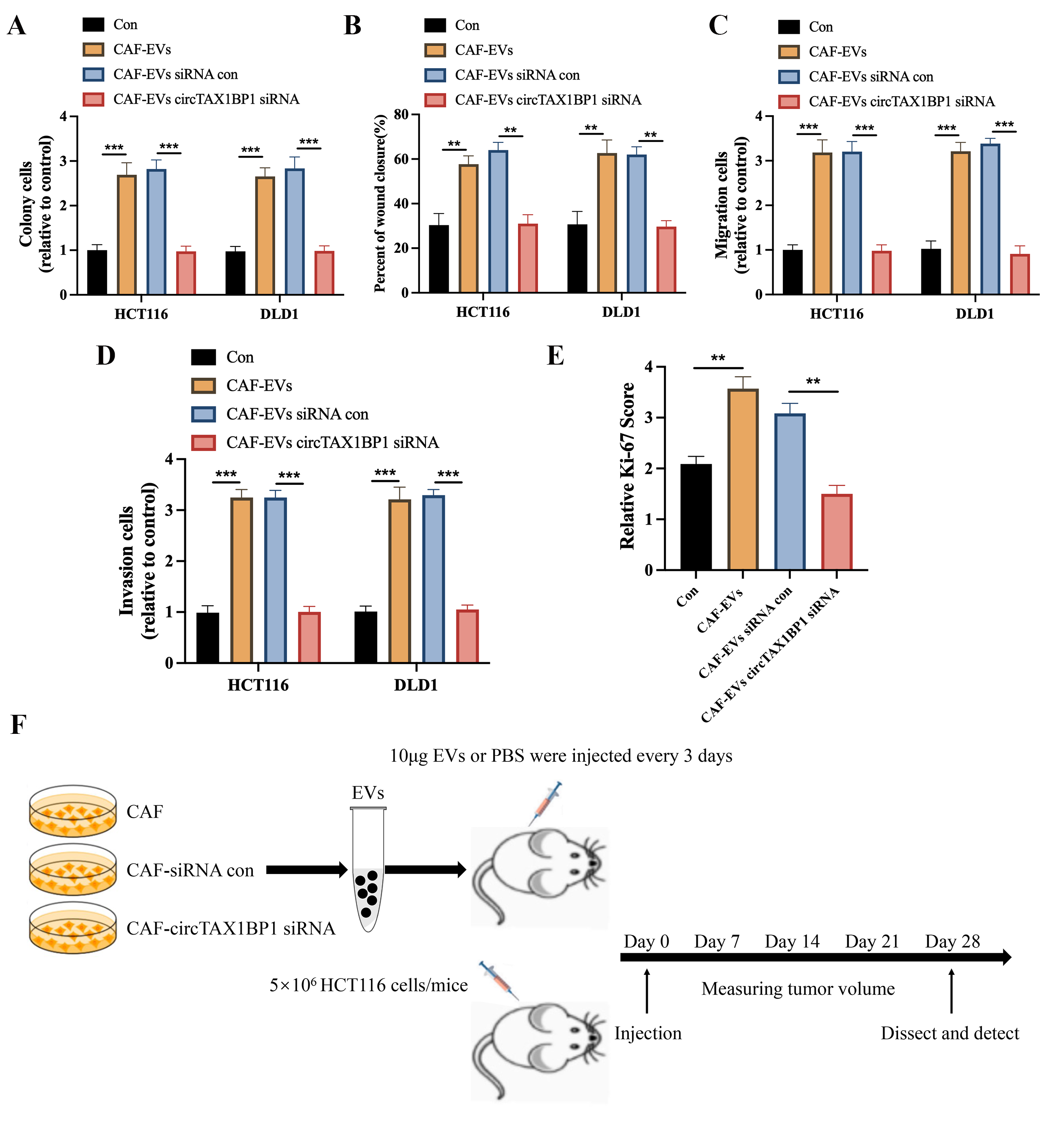


**Supplementary Figure 3. Exosomal circTAX1BP1 derived from CAFs enhances proliferation, migration, and invasion of colorectal cancer (CRC) cells *in* *vitro* and *in vivo*.** A. Colony formation by HCT116 and DLD1 cells was assessed (n = 3). B. Histogram analysis of cell migration distance is shown (n = 3). C–D. Migration and invasion were determined for HCT116 and DLD1 cells (n = 3). E. Graph shows the relative signal intensity scores of Ki-67 (n = 3). F. Schematic illustration of tail vein injection for liver metastasis in a nude mice model. The statistical difference was assessed through 2-tailed Student’s t test in (A-E). All data are presented as mean ± SD of experimental triplicates. **, *P* < 0.01; ***, *P* < 0.001.


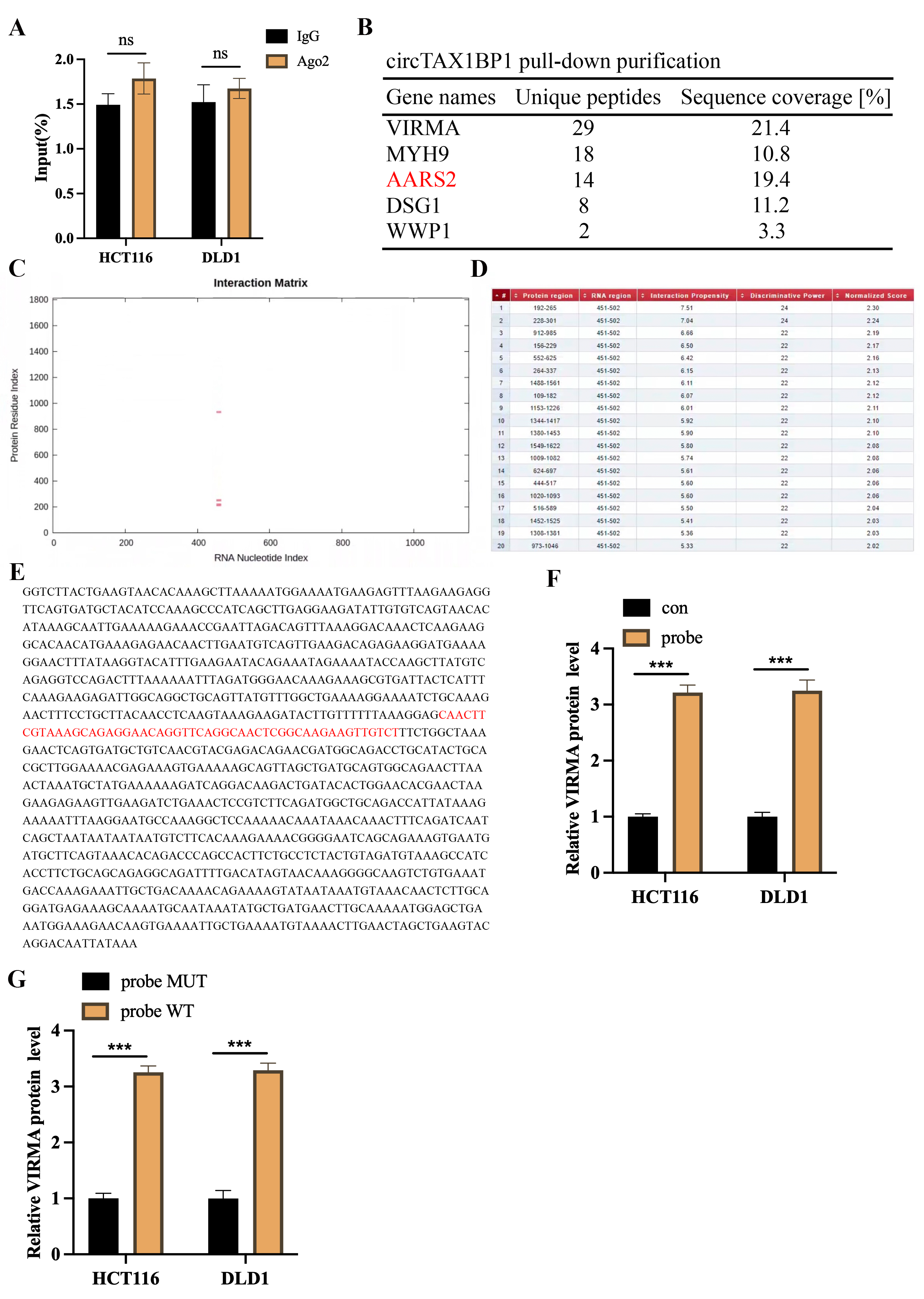


**Supplementary Figure 4. Prediction of circTAX1BP1–VIRMA interaction and analysis of VIRMA expression in colorectal cancer (CRC) cells.** A. Using extracts from HCT116 and DLD1 cells, RIP experiments were performed with an antibody against AGO2 (n = 3). B. Vital proteins identified via mass spectrometry analysis. We marked the protein of interest in red font. C–D. Binding sites between circTAX1BP1 and VIRMA predicted using the catRAPID website. E. The sequence of mutated circTAX1BP1. F–G. The relative protein expression of VIRMA was measured using western blot in CRC cells (n = 3). The statistical difference was assessed through 2-tailed Student’s t test in (A, F-G). All data are presented as mean ± SD of experimental triplicates. ns, *P* > 0.05; ***, *P* < 0.001.


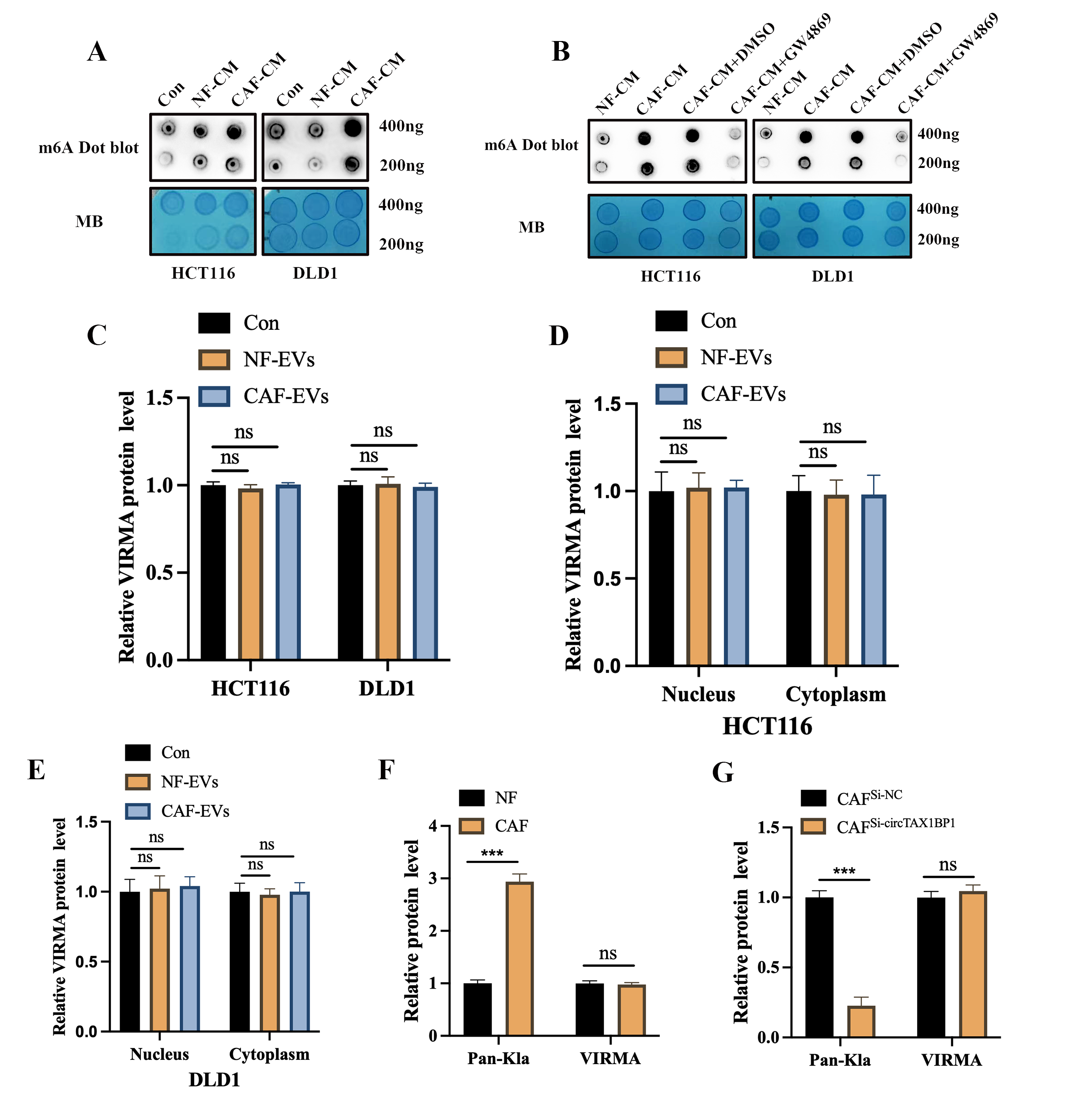


**Supplementary Figure 5. Assessment of m^6^A modification levels and protein expression of VIRMA and Pan-Kla in colorectal cancer (CRC) cells.** A–B. RNA m^6^A dot blot assays were used to detect the m^6^A contents of total mRNA. Methylene blue staining was used to determine the loading control. C–E. The relative protein expression of VIRMA was measured using western blotting in CRC cells (n = 3). F–G. The relative protein expressions of Pan-Kla and VIRMA were measured using western blotting in CRC cells (n = 3). The statistical difference was assessed through one-way ANOVA followed by Dunnett tests in (C-E); and 2-tailed Student’s t test in (F-G). All data are presented as mean ± SD of experimental triplicates. ns, *P* > 0.05; ***, *P* < 0.001.

**
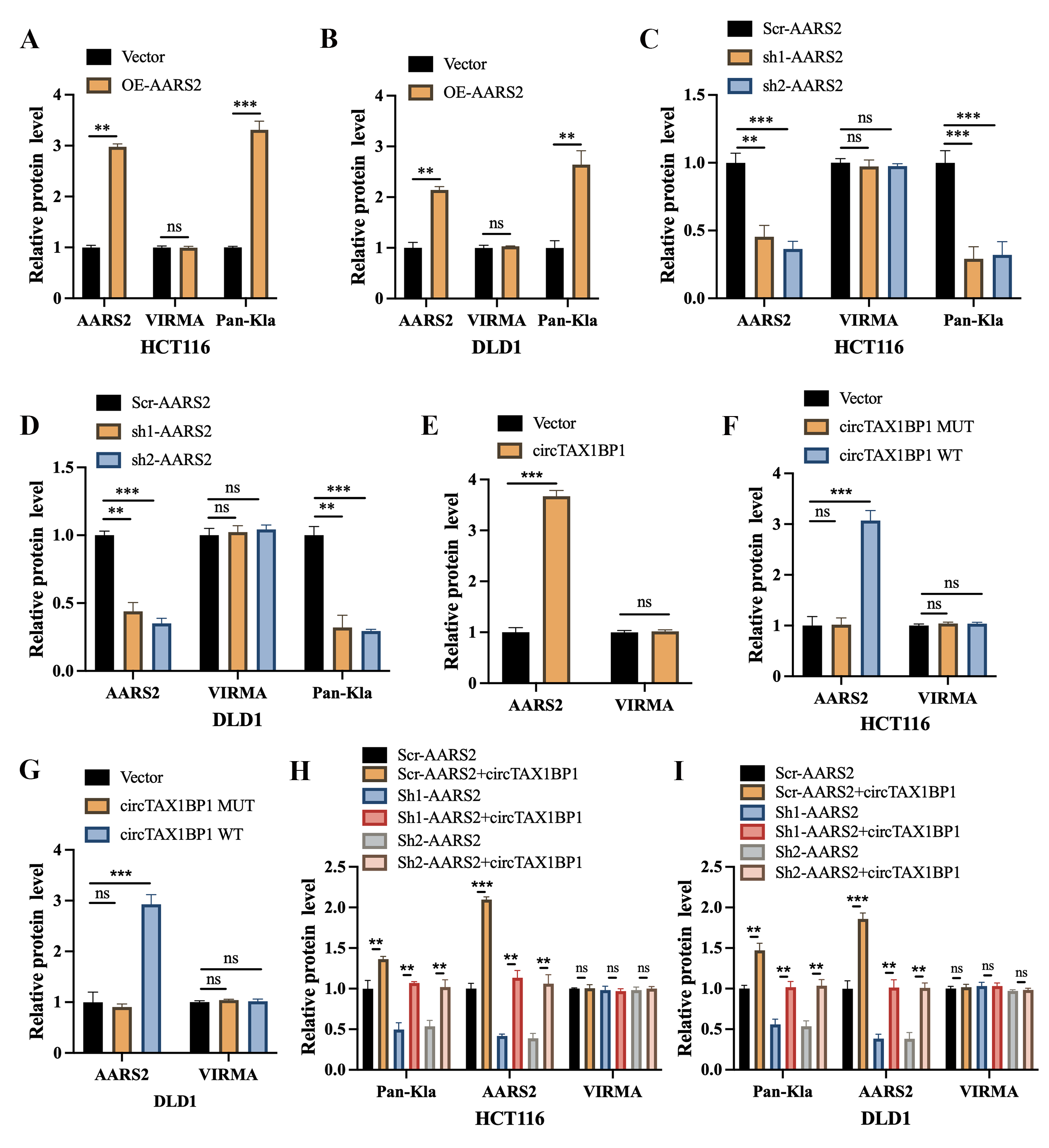
**

**Supplementary Figure 6. Western blot analysis of AARS2, VIRMA, and Pan-Kla protein expression in colorectal cancer (CRC) cells.** A–D. Relative protein expressions of AARS2, VIRMA, and Pan-Kla were measured using western blotting in CRC cells (n = 3). E–G. Relative protein expressions of AARS2 and VIRMA were measured using western blotting in CRC cells (n = 3). H–I. Relative protein expression of Pan-Kla, AARS2, and VIRMA were measured using western blotting in CRC cells (n = 3). The statistical difference was assessed through 2-tailed Student’s t test in (A-B, E, and H-I); and one-way ANOVA followed by Dunnett tests in (C-D, F-G). All data are presented as mean ± SD of experimental triplicates. ns, *P* > 0.05; **, *P* < 0.01; ***, *P* < 0.001.

**
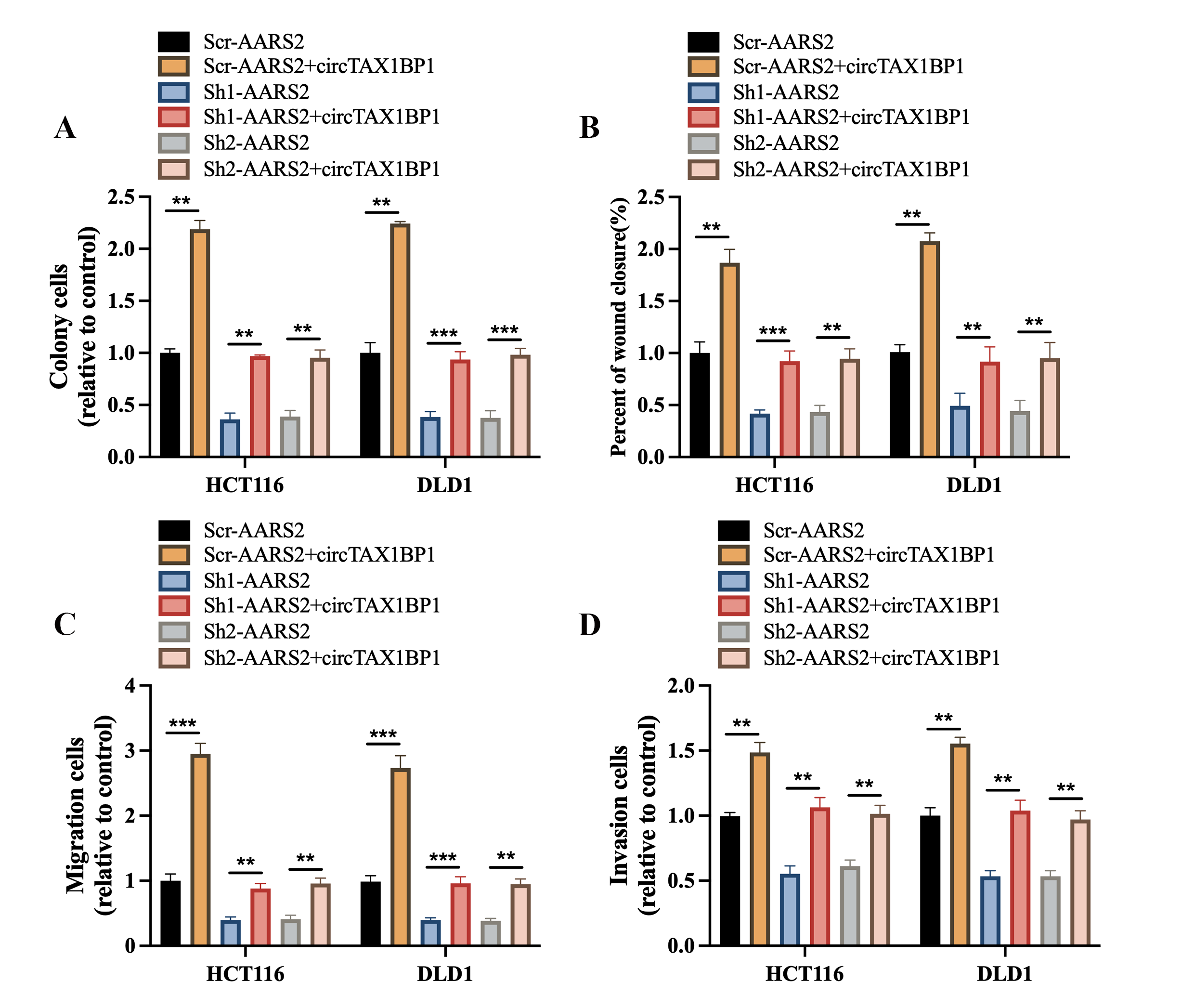
**

**Supplementary Figure 7. Exosomal circTAX1BP1 derived from CAFs enhances proliferation, migration, and invasion of colorectal cancer (CRC) cells.** A. Colony formation by HCT116 and DLD1 cells was assessed (n = 3). B. Histogram analysis of cell migration distance is shown (n = 3). C–D. Migration and invasion were determined for HCT116 and DLD1 cells (n = 3). The statistical difference was assessed through 2-tailed Student’s t test in (A-D). All data are presented as mean ± SD of experimental triplicates. **, *P* < 0.01; ***, *P* < 0.001.

**
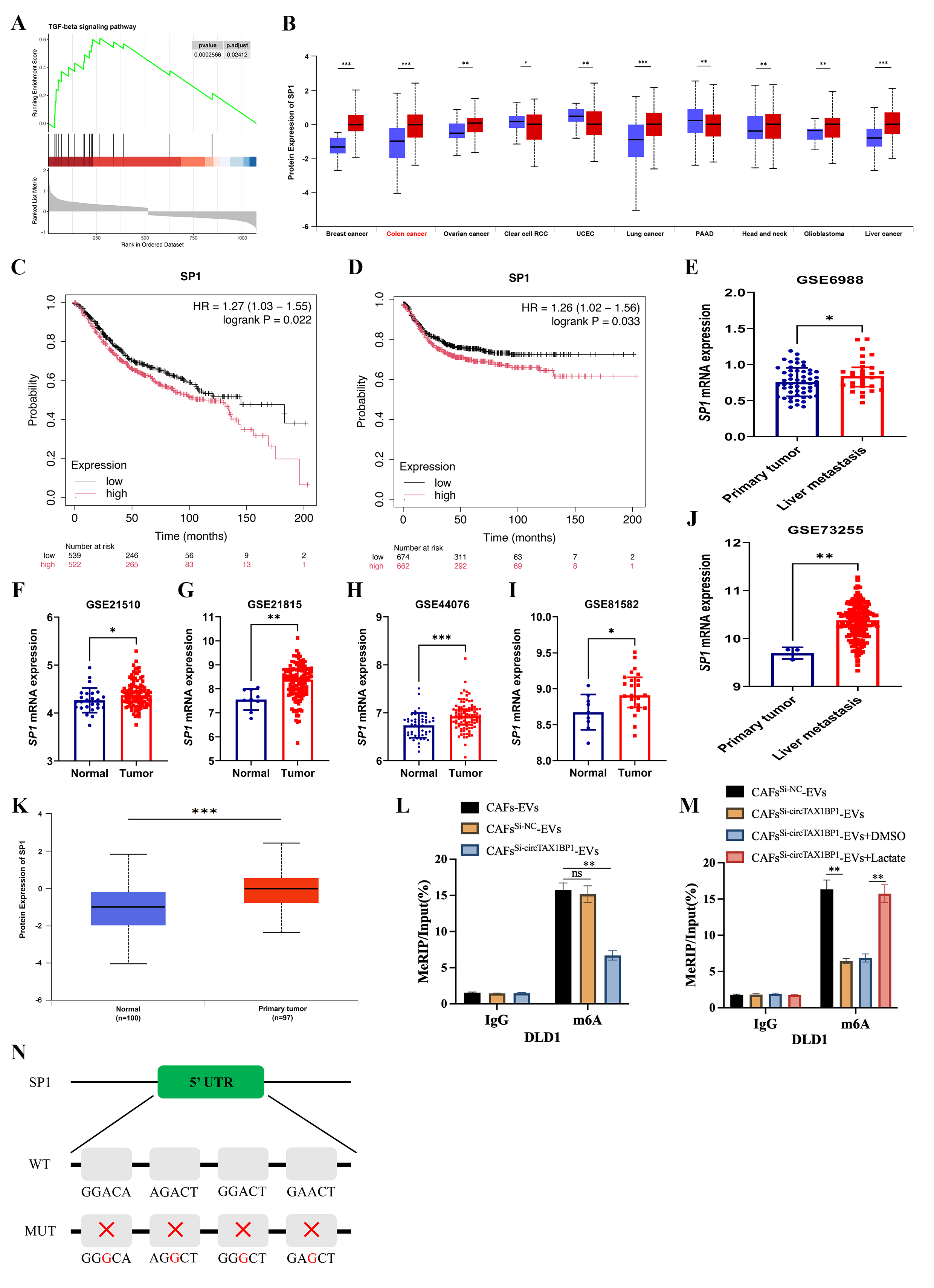
**

**Supplementary Figure 8. Comprehensive analysis of SP1 expression, prognostic value, and m^6^A modulation in colorectal cancer (CRC).** A. GSEA enrichment plot of the TGF-β signalling pathway recognised via RNA-Seq. B. Protein expression of SP1 in tumour and normal samples based on the CPTAC dataset. C–D. Validation of the prognostic value of SP1 in CRC using the KM Plotter tool. The median expression level of SP1 was taken as the cutoff value. *p*-values was calculated by the log-rank (Mantel-Cox) test. E–K. Expression of SP1 for NATs or CRC patients based on the GEO and CPTAC dataset. L–M. MeRIP-qPCR determinations for m^6^A enrichment on SP1 mRNA in DLD1 cells (n = 3). N. The WT or m^6^A consensus sequence mutant SP1 5’UTR was fused with a firefly luciferase reporter. The m^6^A mutant sequence was constructed by replacing A with G. The statistical difference was assessed through nonparametric Mann–Whitney U test in (B, E-K); and one-way ANOVA followed by Dunnett tests in (L); and 2-tailed Student’s t test in (M). All data are presented as mean ± SD of experimental triplicates. ns, *P* > 0.05; *, *P* < 0.05; **, *P* < 0.01; ***, *P* < 0.001.

**
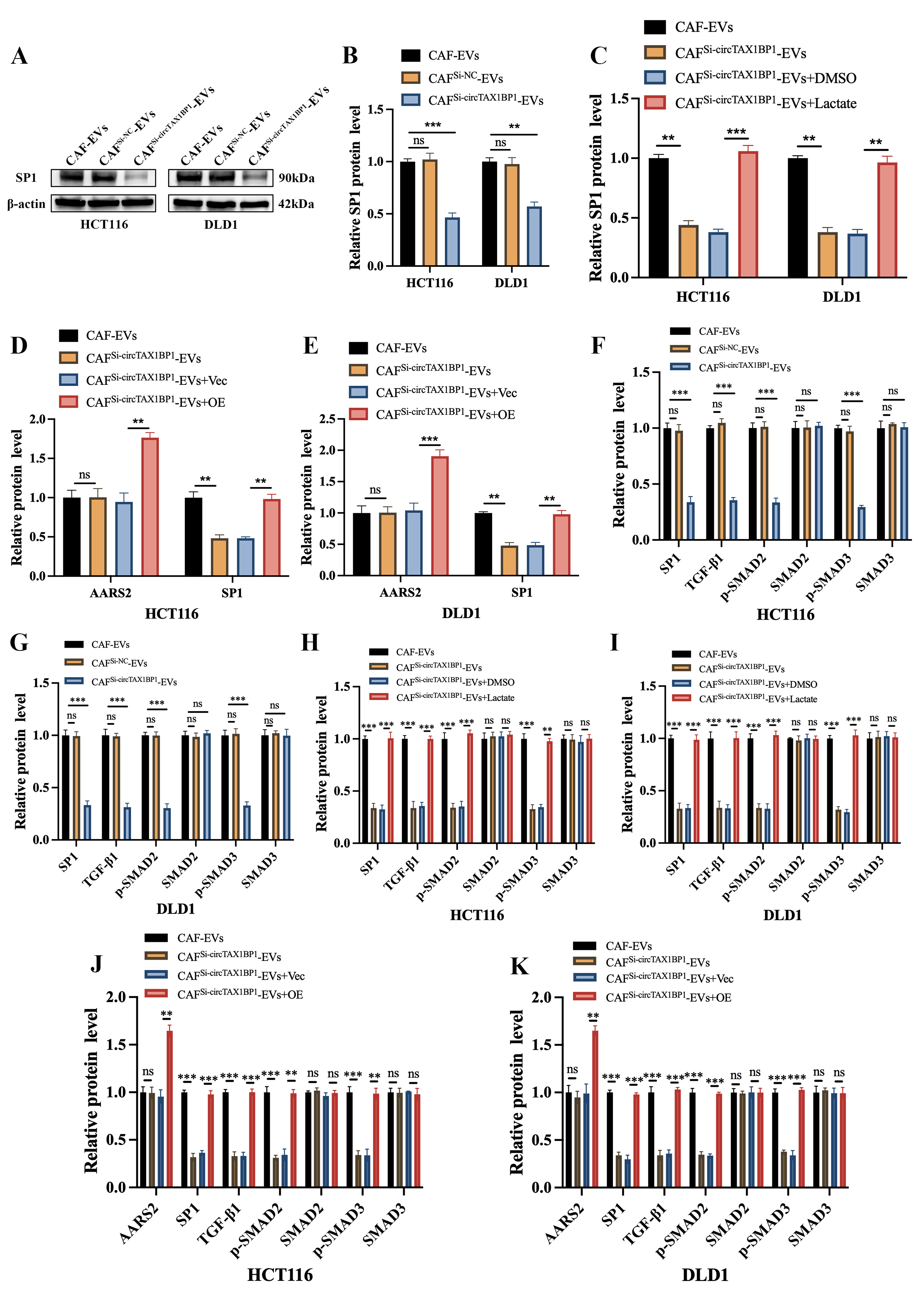
**

**Supplementary Figure 9. Western blot analysis of SP1, AARS2, and TGF-β/SMAD signalling components in colorectal cancer (CRC) cells.** A–C. Relative protein levels of SP1 were determined using western blotting (n = 3). D–E. Relative protein expression of AARS2 and SP1 were measured using western blotting in CRC cells (n = 3). F–I. Relative protein expression of SP1, TGF-β1, p-SMAD2, SMAD2, p-SMAD3, and SMAD3 were measured using western blotting in CRC cells (n = 3). J–K. Relative protein expression of AARS2, SP1, TGF-β1, p-SMAD2, SMAD2, p-SMAD3, and SMAD3 were measured using western blotting in CRC cells (n = 3). The statistical difference was assessed through one-way ANOVA followed by Dunnett tests in (B, F-G); and 2-tailed Student’s t test in (C-E, H-K). All data are presented as mean ± SD of experimental triplicates. ns, *P* > 0.05; **, *P* < 0.01; ***, *P* < 0.001.

**
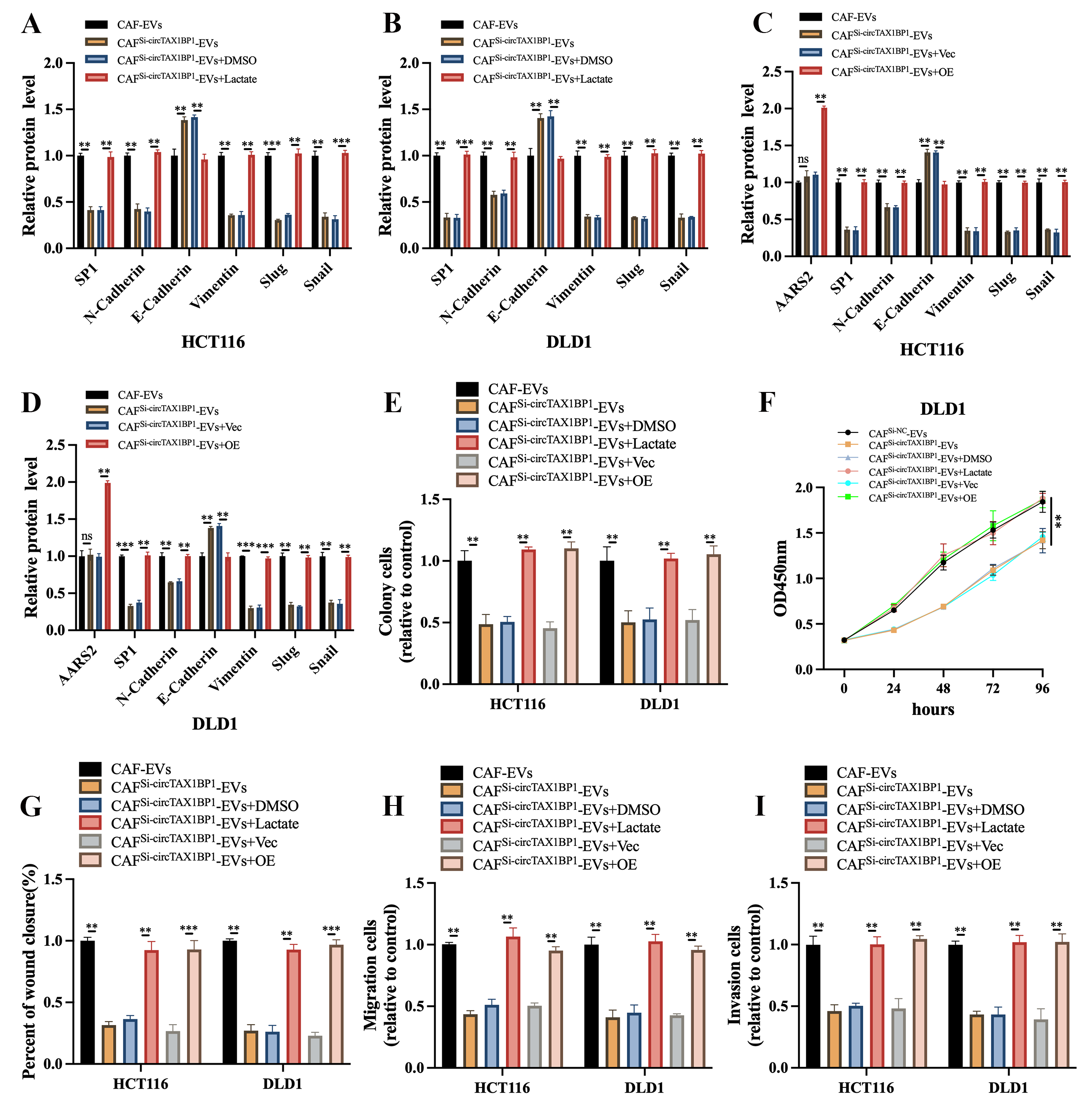
**

**Supplementary Figure 10. Western blot analysis of EMT markers, AARS2 and SP1, and functional assays in colorectal cancer (CRC) cells.** A–B. Relative protein expression of SP1, N-Cadherin, E-Cadherin, Vimentin, Slug, and Snail were measured using western blotting in CRC cells (n = 3). C–D. Relative protein expression of AARS2, SP1, N-Cadherin, E-Cadherin, Vimentin, Slug, and Snail were measured using western blotting in CRC cells (n = 3). E. Colony formation by HCT116 and DLD1 cells was assessed (n = 3). F. Proliferation of DLD1 cells was assessed using CCK-8 assays (n = 3). G. Histogram analysis of cell migration distance is shown (n = 3). H–I. Migration and invasion were determined for HCT116 and DLD1 cells (n = 3). The statistical difference was assessed through 2-tailed Student’s t test in (A-E, G-I); and one-way ANOVA followed by Dunnett tests in (F). All data are presented as mean ± SD of experimental triplicates. ns, *P* > 0.05; **, *P* < 0.01; ***, *P* < 0.001.

**
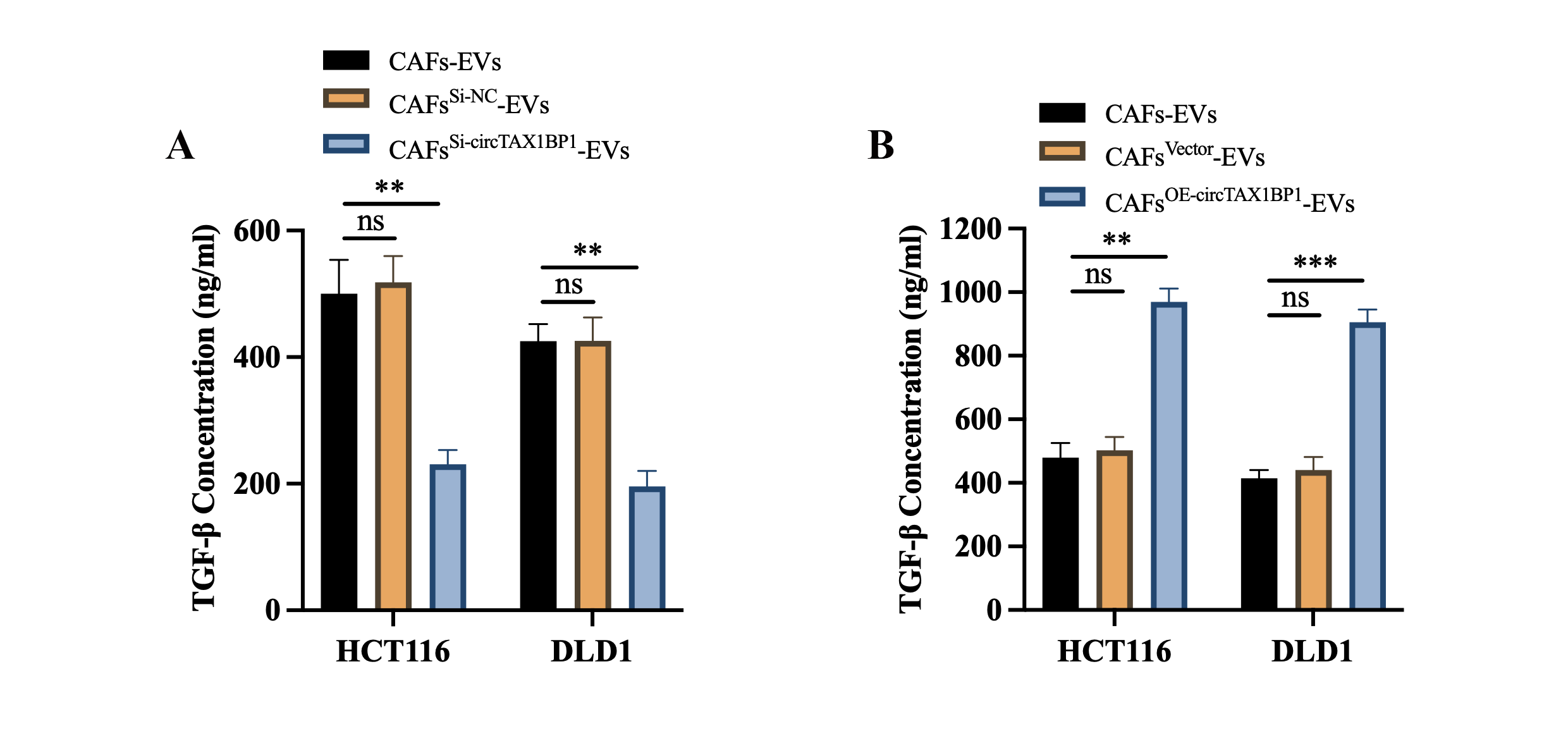
**

**Supplementary Figure 11.** **Measurement of TGF-β levels in supernatants of colorectal cancer (CRC) cells treated with exosomes from CAFs with circTAX1BP1 silencing or overexpression.** A–B. TGF-β levels in the supernatant of CRC cells treated with exosomes from CAFs silencing or overexpressing circTAX1BP1 were measured by ELISA (n = 3). The statistical difference was assessed through one-way ANOVA followed by Dunnett tests in (A-B). All data are presented as mean ± SD of experimental triplicates. ns, *P* > 0.05; **, *P* < 0.01; ***, *P* < 0.001.


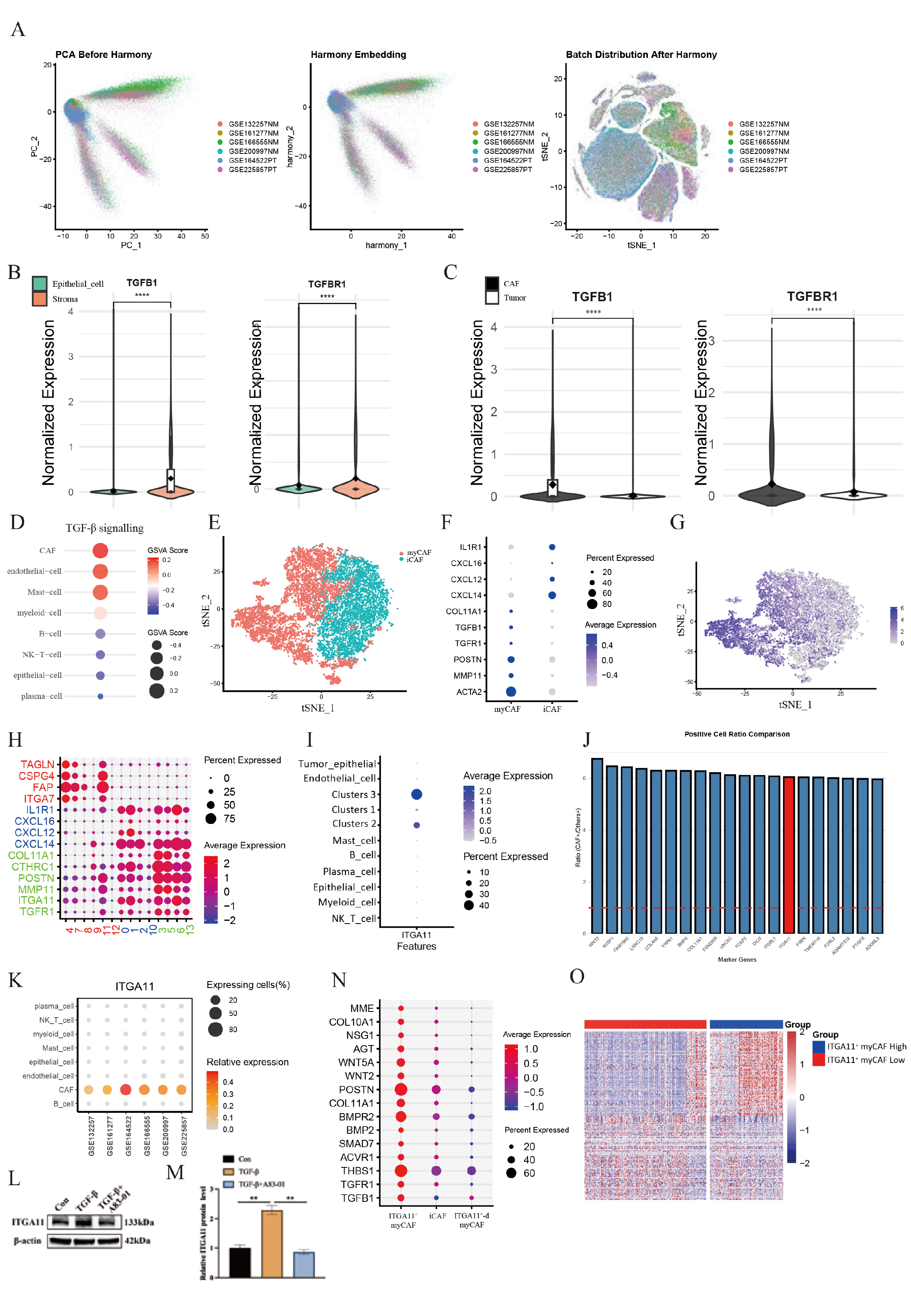


**Supplementary Figure 12. Comprehensive analysis of TGF-β signalling pathway activity in stromal and epithelial cells, with a focus on CAF subtypes and their association with gene expression patterns in TCGA-COAD samples.** A. PCA, Harmony embedding, and t-SNE plots for each dataset before and after applying Harmony. B. Stromal cells versus epithelial cells show markedly elevated expression of the TGF-β signalling pathway-associated genes. C. CAF versus tumour cells show markedly elevated expression of the TGF-β signalling pathway-associated genes. D. TGF-β signalling pathway activity across eight cell clusters, demonstrating predominant activation in CAFs. E. t-SNE plots of the fibroblast cluster showed the iCAF and myCAF cluster. F. The signature expression gene in myCAF and iCAF. G. t-SNE plots show TGF-β signalling score distribute in iCAF and myCAF. H. Fourteen CAF subclusters were re-classified into three cell subtypes, wherein Cluster 1 highly expresses TAGLN, CSPG4, and FAP; Cluster 2 highly expresses IL1R, CXCL16, and CXCL12; and Cluster 3 highly expresses ITGA11, POSTN, and TGFR1. I. Expression profiling of ITGA11 across different cell clusters reveals mainly elevated expression in clusters 3. J. Ratio of the number of cells positive for indicated markers in ITGA11^+^ myCAF with the number of all other cells in the dataset positive for the same marker. K. ITGA11 expression is predominantly elevated in CAFs across multiple datasets. L-M. Relative protein levels of ITGA11 were determined using western blotting (n = 3). N. Expression of TGF-β signalling pathway upstream and downstream genes across three CAF subtypes, predominantly highly expressed in ITGA11^+^ myCAF. O. In TCGA-COAD samples, stratification based on ITGA11^+^ myCAF scores yielded two cohorts, with the high-expression cohort predominantly exhibiting elevated expression of TGF-β signalling pathway and collagen-related genes. The statistical difference was assessed through nonparametric Mann–Whitney U test in (B-C); The statistical difference was assessed through one-way ANOVA followed by Dunnett tests in (M). All data are presented as mean ± SD of experimental triplicates. **, *P* < 0.01; ****, *P* < 0.0001.


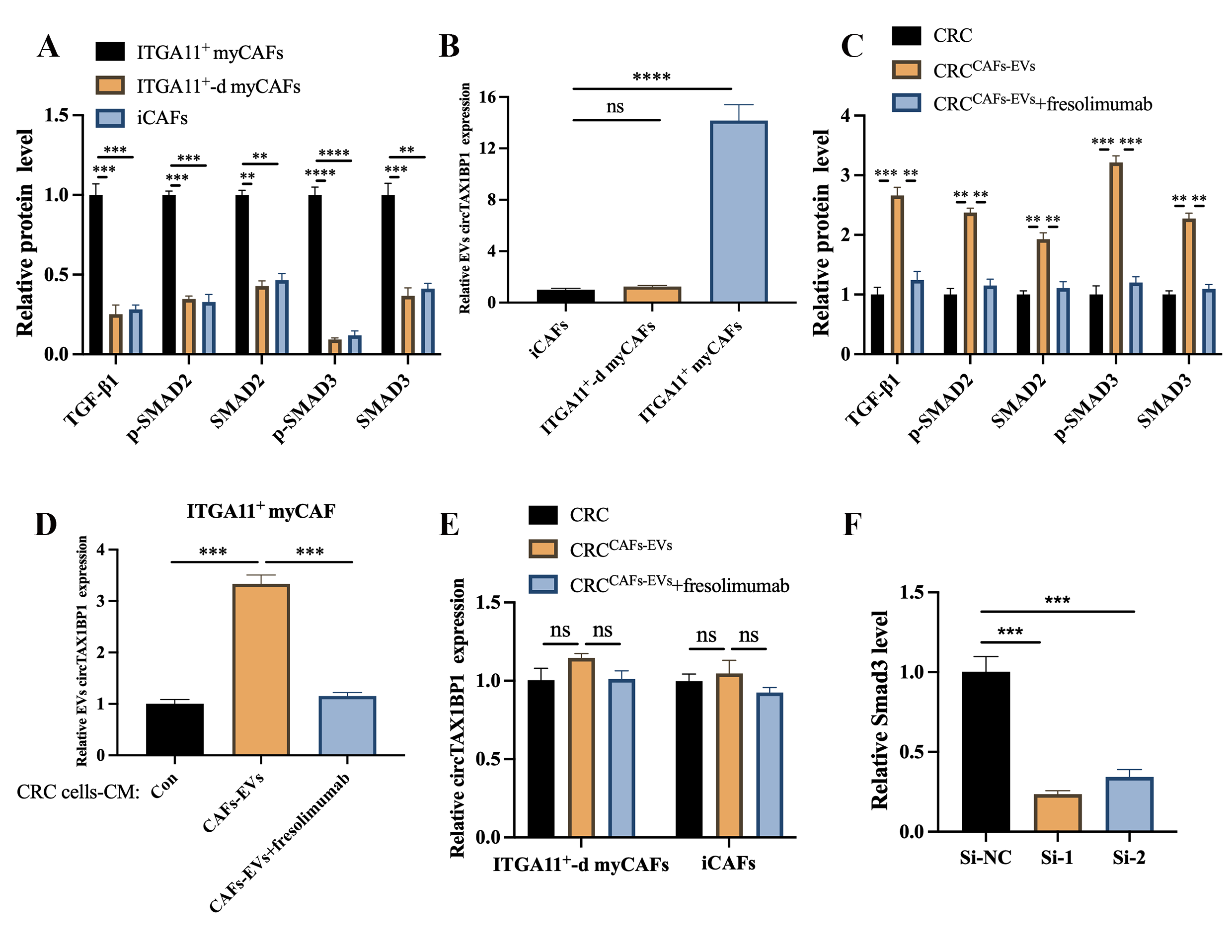


**Supplementary Figure 13. Investigation of TGF-β signaling and circTAX1BP1 expression in CAF subtypes, including responses to colorectal cancer (CRC)-conditioned media (CM) and smad3 knockdown.** A. Relative protein expression of TGF-β1, p-SMAD2, SMAD2, p-SMAD3, and SMAD3 were measured using western blotting in CAF cells (n = 3). B. qRT-PCR analysis of EVs circTAX1BP1 expression in iCAFs, ITGA11^+^-d myCAFs, or ITGA11^+^ myCAFs (n = 3). C. Relative protein expression of TGF-β1, p-SMAD2, SMAD2, p-SMAD3, and SMAD3 were measured using western blotting in ITGA11^+^ myCAFs (n = 3). D. qRT-PCR analysis of EVs circTAX1BP1 expression in ITGA11^+^ myCAFs cultured with indicated CM from CRC cells with or without fresolimumab treatment (n = 3). E. qRT-PCR analysis of circTAX1BP1 expression in ITGA11^+^-d myCAFs or iCAFs cultured with indicated CM from CRC cells with or without fresolimumab treatment (n = 3). F. ITGA11^+^ myCAFs were exposed to smad3 siRNA for 24 h (n = 3). The statistical difference was assessed through one-way ANOVA followed by Dunnett tests in (A-F). All data are presented as mean ± SD of experimental triplicates. ns, *P* > 0.05; **, *P* < 0.01; ***, *P* < 0.001; ****, *P* < 0.0001.


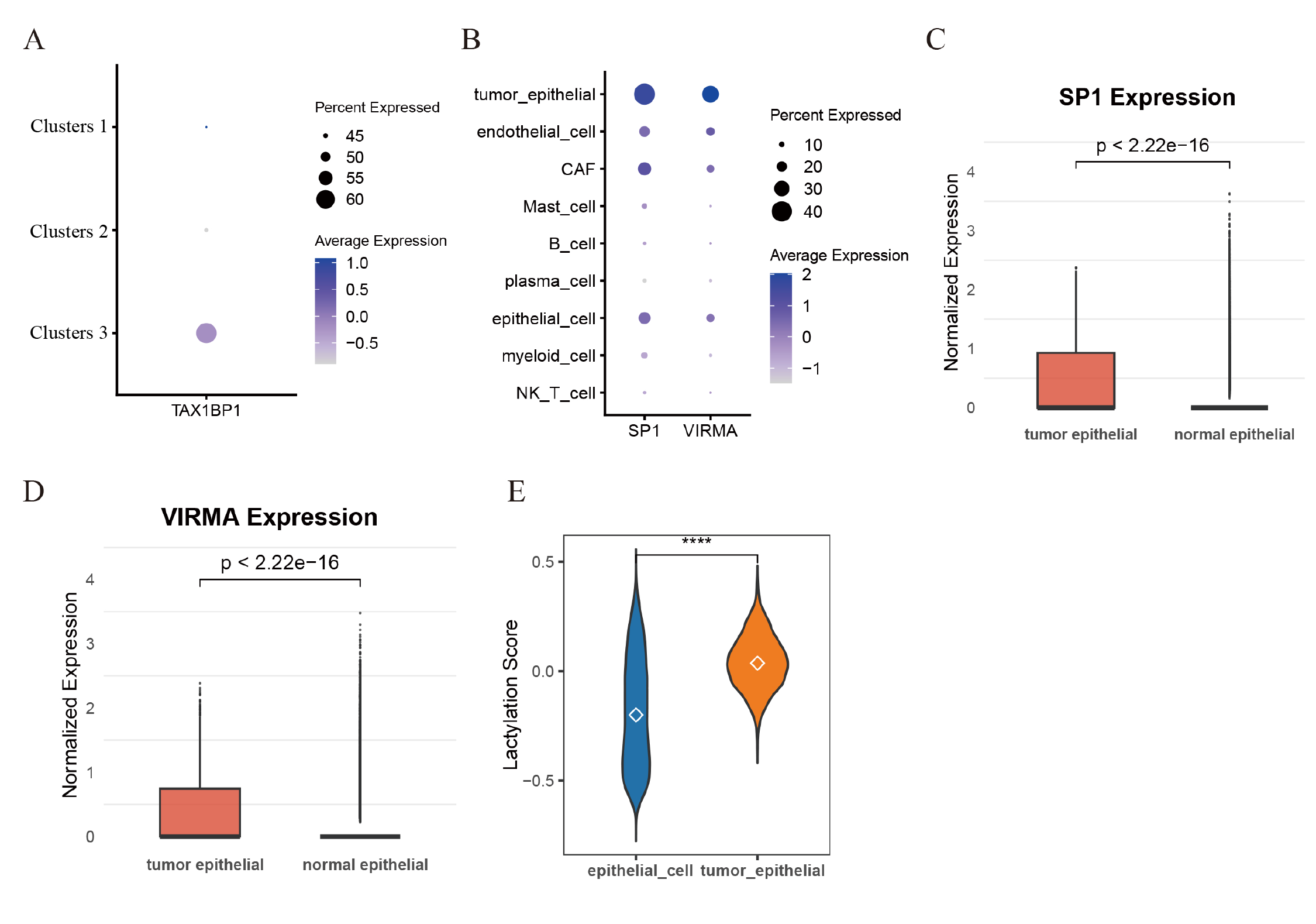


**Supplementary Figure 14. scRNA data support lactaylation VIRMA regulate TGF-β drive ITGA11^+^ myCAFs upregulate EV-packed circTAX1BP1 to form a feedback loop.** A. Expression of TAX1BP1 in the 3 CAF subtypes, with the highest expression level observed in clusters 3. B-C. Tumor epithelial cells show significantly higher expression of SP1 and VIRMA compared to normal epithelial cells (*p*<0.05). D. Expression profiling of SP1 and VIRMA across different cell clusters reveals elevated expression in tumor epithelial cells. E. GSVA analysis lactaylation scores in tumor epithelial cells and epithelial cells. The statistical difference was assessed through nonparametric Mann–Whitney U test in (C-E). All data are presented as mean ± SD of experimental triplicates. ***, *P* < 0.001.


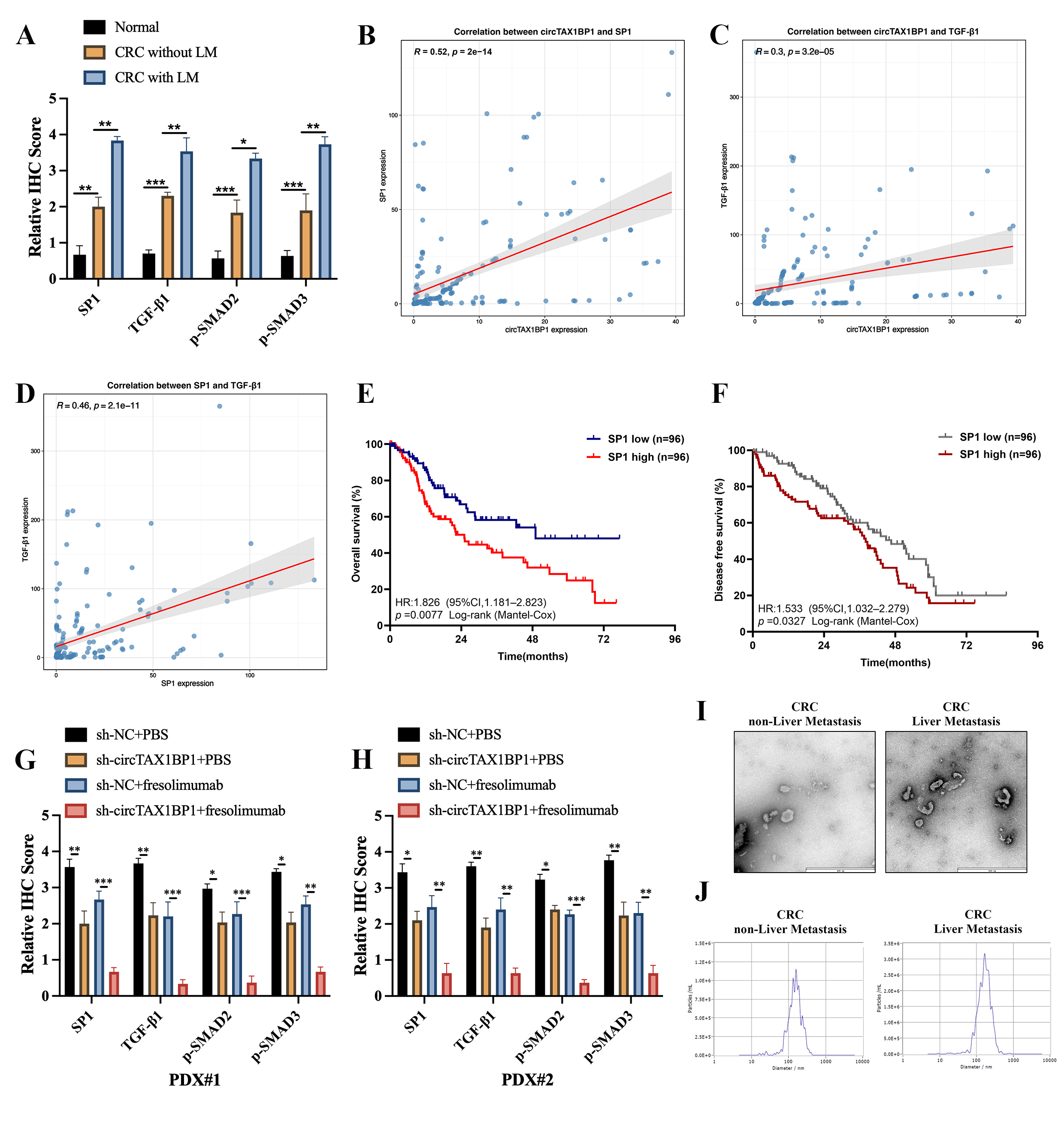


**Supplementary Figure 15. Comprehensive analysis of circTAX1BP1, SP1, TGF-β1, p-Smad2/3 in colorectal cancer (CRC), patient survival outcomes, and characterization of plasma exosomes.** A. Graph shows the relative signal intensity scores of SP1, TGF-β1, p-Smad2, and p-Smad3 (n = 3). B. The correlation between circTAX1BP1 and SP1 in CRC tissues was analyzed by Pearson correlation coefficients. C. The correlation between circTAX1BP1 and TGF-β1 in CRC tissues was analyzed by Pearson correlation coefficients. D. The correlation between SP1 and TGF-β1 in CRC tissues was analyzed by Pearson correlation coefficients. E–F. Kaplan–Meier curves of the OS and DFS of patients with CRC with low vs. high SP1 expression levels. The cutoff value is the median. *p*-values was calculated by the log-rank (Mantel-Cox) test. G–H. Graph shows the relative signal intensity scores of SP1, TGF-β1, p-Smad2, and p-Smad3 (n = 3). I. Representative images of plasma exosomes derived from patients with CRC analysed via transmission electron microscopy. Scale bar, 600 nm. J. Size distribution of the isolated plasma exosomes analysed via nanoparticle tracking. Spearman correlation analysis was used in (B-D). The statistical difference was assessed through one-way ANOVA followed by Dunnett tests in (A); and 2-tailed Student’s t test in (G-H). All data are presented as mean ± SD of experimental triplicates. *, *P* < 0.05; **, *P* < 0.01; ***, *P* < 0.001.
