# Supplementary material for: Extracellular Vesicle‐Packaged circTAX1BP1 from Cancer‐Associated Fibroblasts Regulates RNA m6A Modification through Lactylation of VIRMA in Colorectal Cancer Cells
Source: Adv Sci (Weinh). 2025 Sep 29;12(47):e14008. doi: 10.1002/advs.202514008 (PMC12713077; doi:10.1002/advs.202514008)
Supplement: Supplementary file 3 — Supporting Information [file ADVS-12-e14008-s002.docx]

**Table S1:** Correlation between circTAX1BP1 expression and clinicopathologic characteristics of colorectal cancer patients (n=192)

| **Characteristics** | **No. of cases** | **circTAX1BP1 expression** | | |
| --- | --- | --- | --- | --- |
|  |  | **Low** | **High** | ***P*-value^i^** |
| **Gender** |  |  |  | 0.4634 |
| Male | 113 | 59 | 54 |  |
| Female | 79 | 37 | 42 |  |
| **Age(years)** |  |  |  | 0.3102 |
| < 65 | 87 | 47 | 40 |  |
| ≥ 65 | 105 | 49 | 56 |  |
| **Tumor location** |  |  |  |  |
| left hemicolon | 136 | 72 | 64 | 0.204 |
| right hemicolon | 56 | 24 | 32 |  |
| **Tumor sizes(cm)** |  |  |  | **0.01327^*^** |
| <5 | 109 | 63 | 46 |  |
| ≥5 | 83 | 33 | 50 |  |
| **Differentiation** |  |  |  |  |
| well and moderately | 75 | 36 | 39 | 0.6572 |
| poorly | 117 | 60 | 57 |  |
| **T stage** |  |  |  | **0.01149^*^** |
| T1/T2 | 73 | 45 | 28 |  |
| T3/T4 | 119 | 51 | 68 |  |
| **N stage** |  |  |  | **<0.01^**^** |
| N0 | 70 | 45 | 25 |  |
| N1/N2 | 122 | 51 | 71 |  |
| **Liver metastasis** |  |  |  | **<0.001^***^** |
| Negative | 144 | 85 | 59 |  |
| Positive | 48 | 11 | 37 |  |

Differences among variable were assessed by the chi-square test,

^*^ *P* <0.05, ^**^ *P* <0.01, ^***^ *P* <0.001.
