# Supplementary material for: Extracellular Vesicle‐Packaged circTAX1BP1 from Cancer‐Associated Fibroblasts Regulates RNA m6A Modification through Lactylation of VIRMA in Colorectal Cancer Cells
Source: Adv Sci (Weinh). 2025 Sep 29;12(47):e14008. doi: 10.1002/advs.202514008 (PMC12713077; doi:10.1002/advs.202514008)
Supplement: Supplementary file 4 — Supporting Information [file ADVS-12-e14008-s007.docx]

**Table S2:** Univariate and multivariate analysis of Overall Survival (OS) for circTAX1BP1 expression in colorectal cancer patients (*n* = 192)

| **Variables** | **Univariate analysis** | | | **Multivariate analysis** | | |
| --- | --- | --- | --- | --- | --- | --- |
|  | **HR** | **95%CI** | ***P*-value^i^** | **HR** | **95%CI** | ***P*-value^i^** |
| Age (≥65 vs. <65) | 1.068 | 0.680-1.676 | 0.775 |  |  |  |
| Gender  (Female vs. Male) | 0.833 | 0.534-1.297 | 0.419 |  |  |  |
| Tumor location  (right vs. left hemicolon) | 1.380 | 0.883-2.156 | 0.157 |  |  |  |
| Tumor sizes  (≥5 vs. <5) | 1.500 | 0.966-2.327 | 0.071 |  |  |  |
| Differentiation  (poorly vs. well and moderately) | 1.261 | 0.792-2.005 | 0.328 |  |  |  |
| T stage  (T3/T4 vs. T1/T2) | 1.849 | 1.164-2.938 | **<0.01^**^** | 1.880 | 1.168-3.025 | **<0.01^**^** |
| N stage  (N1/N2 vs. N0) | 1.688 | 1.044-2.730 | **0.033^*^** | 1.397 | 0.845-2.309 | 0.192 |
| Liver metastasis  (positive vs. negative) | 2.063 | 1.243-3.426 | **<0.01^**^** | 1.914 | 1.103-3.319 | **0.021^*^** |
| circTAX1BP1 expression  (High vs. Low) | 2.402 | 1.536-3.756 | **<0.001^***^** | 1.921 | 1.188-3.107 | **<0.01^**^** |

Abbreviations: HR = hazard ratio; 95%CI =95% confidence interval.

Cox regression analysis, ^*^ *P* <0.05, ^**^ *P* <0.01, ^***^ *P* <0.001.
