# Supplementary material for: Extracellular Vesicle‐Packaged circTAX1BP1 from Cancer‐Associated Fibroblasts Regulates RNA m6A Modification through Lactylation of VIRMA in Colorectal Cancer Cells
Source: Adv Sci (Weinh). 2025 Sep 29;12(47):e14008. doi: 10.1002/advs.202514008 (PMC12713077; doi:10.1002/advs.202514008)
Supplement: Supplementary file 5 — Supporting Information [file ADVS-12-e14008-s009.docx]

**Table S3:** Univariate and multivariate analysis of Disease-Free Survival (DFS) for circTAX1BP1 expression in colorectal cancer patients (*n* = 192)

| **Variables** | **Univariate analysis** | | | **Multivariate analysis** | | |
| --- | --- | --- | --- | --- | --- | --- |
|  | **HR** | **95%CI** | ***P*-value^i^** | **HR** | **95%CI** | ***P*-value^i^** |
| Age (≥65 vs. <65) | 1.158 | 0.777-1.726 | 0.472 |  |  |  |
| Gender  (Female vs. Male) | 0.944 | 0.634-1.406 | 0.776 |  |  |  |
| Tumor location  (right vs. left hemicolon) | 1.193 | 0.793-1.793 | 0.397 |  |  |  |
| Tumor sizes  (≥5 vs. <5) | 1.180 | 0.794-1.755 | 0.413 |  |  |  |
| Differentiation  (poorly vs. well and moderately) | 1.205 | 0.793-1.831 | 0.381 |  |  |  |
| T stage  (T3/T4 vs. T1/T2) | 1.312 | 0.864-1.992 | 0.202 |  |  |  |
| N stage  (N1/N2 vs. N0) | 1.715 | 1.130-2.602 | **0.011^*^** | 1.645 | 1.073-2.522 | **0.023*** |
| Liver metastasis  (positive vs. negative) | 1.916 | 1.218-3.013 | **0.005^**^** | 1.676 | 1.032 -2.723 | **0.037*** |
| circTAX1BP1 expression  (High vs. Low) | 1.985 | 1.331-2.960 | **<0.001^***^** | 1.625 | 1.056-2.499 | **0.027*** |

Abbreviations: HR = hazard ratio; 95%CI =95% confidence interval.

Cox regression analysis, ^*^ *P* <0.05, ^**^ *P* <0.01, ^***^ *P* <0.001.
