# Supplementary material for: Extracellular Vesicle‐Packaged circTAX1BP1 from Cancer‐Associated Fibroblasts Regulates RNA m6A Modification through Lactylation of VIRMA in Colorectal Cancer Cells
Source: Adv Sci (Weinh). 2025 Sep 29;12(47):e14008. doi: 10.1002/advs.202514008 (PMC12713077; doi:10.1002/advs.202514008)
Supplement: Supplementary file 7 — Supporting Information [file ADVS-12-e14008-s008.docx]

**Table S5:** Primers used for circRNAs and gene qRT-PCR analysis

| **Gene name** | **Sense** | **Anti-sense** | **Product size** |
| --- | --- | --- | --- |
| Linear TAX1BP1 | AAGAAACAGCACAACTTCGAGA | TGGATGTAGCATCACTGAACCT | 171 |
| circTAX1BP1 | CTAGCTGAAGTACAGGAC | TCTTCCTCAAGCTGATGGGC | 125 |
| circTCONS | TGGGAAGACTTGGTTGTGCA | TTCTTGGAAGGCTGAGGCAG | 136 |
| circRNASEH2B | GCTTCCAGAACCTTCAGCCT | TGGCTCCTTCTCCTGAACAG | 127 |
| ACTA2 | GTGTTGCCCCTGAAGAGCAT | GCTGGGACATTGAAAGTCTCA | 109 |
| FAP | ATGAGCTTCCTCGTCCAATTCA | AGACCACCAGAGAGCATATTTTG | 215 |
| FSP1 | GATGAGCAACTTGGACAGCAA | CTGGGCTGCTTATCTGGGAAG | 123 |
| CD90 | ATCGCTCTCCTGCTAACAGTC | CTCGTACTGGATGGGTGAACT | 135 |
| POSTN | CTCATAGTCGTATCAGGGGTCG | ACACAGTCGTTTTCTGTCCAC | 138 |
| MMP11 | CCGCAACCGACAGAAGAGG | ATCGCTCCATACCTTTAGGGC | 145 |
| COL11A1 | ACCCTCGCATTGACCTTCC | TTTGTGCAAAATCCCGTTGTTT | 128 |
| SP1 | TGGCAGCAGTACCAATGGC | CCAGGTAGTCCTGTCAGAACTT | 126 |
| TGF-β1 | GGCCAGATCCTGTCCAAGC | GTGGGTTTCCACCATTAGCAC | 201 |
| SMAD3 | CCATCTCCTACTACGAGCTGAA | CACTGCTGCATTCCTGTTGAC | 149 |
| GAPDH | GGACCTGACCTGCCGTCTAG | GTAGCCCAGGATGCCCTTGA | 100 |
| U6 | CTCGCTTCGGCAGCACA | AACGCTTCACGAATTTGCGT | 94 |
