# Supplementary material for: Extracellular Vesicle‐Packaged circTAX1BP1 from Cancer‐Associated Fibroblasts Regulates RNA m6A Modification through Lactylation of VIRMA in Colorectal Cancer Cells
Source: Adv Sci (Weinh). 2025 Sep 29;12(47):e14008. doi: 10.1002/advs.202514008 (PMC12713077; doi:10.1002/advs.202514008)
Supplement: Supplementary file 8 — Supporting Information [file ADVS-12-e14008-s005.docx]

**Table S6:** The sequences of siRNAs

| **siRNA name** | **Sense strand** | **Anti-sense stand** |
| --- | --- | --- |
| si-circTAX1BP1-1 | AGGACAAUUAUAAAGGUCUTT | AGACCUUUAUAAUUGUCCUTT |
| si-circTAX1BP1-2 | ACAAUUAUAAAGGUCUUACTT | GUAAGACCUUUAUAAUUGUTT |
| si-circTAX1BP1-3 | GACAAUUAUAAAGGUCUUATT | UAAGACCUUUAUAAUUGUCTT |
| si-AARS2-1 | CCAUCAUACCUUCUUUGAAAUTT | AUUUCAAAGAAGGUAUGAUGGTT |
| si-AARS2-2 | CCUGGUCUUCAUGCAACACAATT | UUGUGUUGCAUGAAGACCAGGTT |
| si-SMAD3-1 | GGACGAGGUCUGCGUGAAUCC | AUUCACGCAGACCUCGUCCUU |
| si-SMAD3-2 | GUCAUACAGCUCAAAUGUGAU | CACAUUUGAGCUGUAUGACUU |
