# Supplementary material for: Extracellular Vesicle‐Packaged circTAX1BP1 from Cancer‐Associated Fibroblasts Regulates RNA m6A Modification through Lactylation of VIRMA in Colorectal Cancer Cells
Source: Adv Sci (Weinh). 2025 Sep 29;12(47):e14008. doi: 10.1002/advs.202514008 (PMC12713077; doi:10.1002/advs.202514008)
Supplement: Supplementary file 9 — Supporting Information [file ADVS-12-e14008-s004.docx]

**Table S7:** Primers used for ChIP analysis

| **Gene name** | **Sense** | **Anti-sense** | **Product size** |
| --- | --- | --- | --- |
| TGF-β1 Site1 | GCACTGGGGAGCTATGGAAG | GACTCCTGCTGATTCCCCAC | 204 |
| TGF-β1 Site2 | ATCCATGTTCATGTCTGCCGT | TAATCCACCTTCCACCTGGC | 201 |
| circTAX1BP1 P1 | GTGCTGCCCCAGATAGTGAG | ACAAGCATGAGAGACCGCAC | 205 |
| circTAX1BP1 P2 | TCCCAATCAAACACAGGACAAC | CCTCCCAGGTTCGAGTGATT | 201 |
